# Genomic evidence for the suitability of Göttingen Minipigs with a rare seizure phenotype as a model for human epilepsy

**DOI:** 10.1007/s10048-024-00750-2

**Published:** 2024-02-21

**Authors:** Pardis Najafi, Christian Reimer, Jonathan D. Gilthorpe, Kirsten R. Jacobsen, Maja Ramløse, Nora-Fabienne Paul, Henner Simianer, Jens Tetens, Clemens Falker-Gieske

**Affiliations:** 1 Department of Animal Sciences, Georg-August-University, Burckhardtweg 2, 37077 Göttingen, Germany; 2 Center for Integrated Breeding Research, Georg-August-University, Albrecht-Thaer-Weg 3, 37075 Göttingen, Germany; 3 Friedrich-Loeffler-Institute, Federal Research Institute for Animal Health, Höltystr. 10, 31535 Neustadt, Germany; 4 Department of Integrative Medical Biology, Umeå University, 901 87 Umeå, Sweden; 5 Ellegaard Göttingen Minipigs A/S, Sorø Landevej 302, 4261 Dalmose, Denmark

**Keywords:** Göttingen Minipigs, Epilepsy, Seizure, Voltage-gated calcium channel, Genomics, Transcriptomics

## Abstract

Epilepsy is a complex genetic disorder that affects about 2% of the global population. Although the frequency and severity of epileptic seizures can be reduced by a range of pharmacological interventions, there are no disease-modifying treatments for epilepsy. The development of new and more effective drugs is hindered by a lack of suitable animal models. Available rodent models may not recapitulate all key aspects of the disease. Spontaneous epileptic convulsions were observed in few Göttingen Minipigs (GMPs), which may provide a valuable alternative animal model for the characterisation of epilepsy-type diseases and for testing new treatments. We have characterised affected GMPs at the genome level and have taken advantage of primary fibroblast cultures to validate the functional impact of fixed genetic variants on the transcriptome level. We found numerous genes connected to calcium metabolism that have not been associated with epilepsy before, such as *ADORA2B*, *CAMK1D*, *ITPKB*, *MCOLN2*, *MYLK*, *NFATC3*, *PDGFD*, and *PHKB*. Our results have identified two transcription factor genes, *EGR3* and *HOXB6*, as potential key regulators of *CACNA1H*, which was previously linked to epilepsy-type disorders in humans. Our findings provide the first set of conclusive results to support the use of affected subsets of GMPs as an alternative and more reliable model system to study human epilepsy. Further neurological and pharmacological validation of the suitability of GMPs as an epilepsy model is therefore warranted.

**Supplementary Information:**

The online version contains supplementary material available at 10.1007/s10048-024-00750-2.

## Introduction

Epilepsy is one of the most common neurological diseases with a prevalence of almost 2% in the general human population [1]. It is characterised by two or more unprovoked seizures defined by unexpected and uncontrolled brain electrical activity. Epilepsy can result in an alteration of behaviour, movement, and sensation and typically has a significant impact on a person’s quality of life due to subsequent physical injury to the brain, emotional distress, and social isolation. While there are some treatments available, many people with epilepsy continue to experience seizures despite medical intervention, making it a serious and ongoing health challenge. Epilepsy can occur following structural abnormalities of the central nervous system, metabolic disturbances, and genetic mutations [2]. Genetics plays a prominent role in the occurrence of epilepsy, but genetic causes are still poorly defined.

Several epilepsy-associated genes have been identified, including those that encode ion channels and neurotransmitters [3]. Ion channels are important for the establishment of neuronal action potentials and in maintaining ionic homeostasis by gating ionic flux through the plasma membrane, and in cell volume regulation, which is important for the excitability of neurons [4]. Due to their function in the regulation of both neuronal excitation and inhibition, ion channels have a potential key role in epileptogenesis [5]. In particular, low-voltage activated (T-type) Ca^2+^ channels [6], which play a key role in amplifying the excitatory potentials of postsynaptic neurons [7], are of particular interest. T-type Ca^2+^ channels include three subunits of a CaV3-type pore-forming channel (CaV3.1, CaV3.2, and CaV3.3) and are encoded by the *CACNA1* gene family members (*CACNA1I*, *CACNA1G*, and *CACNA1H*, respectively). Mutations affecting the expression of CaV3.2 channels in the hippocampus and thalamus are related to the occurrence of idiopathic generalised epilepsy [8]. Although ion channels play an important role in several types of epilepsy, other pathways and transcription factors lead to neuronal hyperexcitability [2]. However, little is known about the function of transcription factors and related pathways in the control of the CaV3.2 gene expression.

Epileptic rodent models (induced or genetic) have been used to better understand epilepsy in humans [9]. However, the value of rodent models is limited by their phenotype, particularly in models where seizures are absent. Hence, key aspects of the disease may not be faithfully recapitulated [9]. It is important to develop other animal models that more closely resemble human neurophysiology, to gain a more comprehensive understanding of epilepsy [10]. Recently, GMPs have been used extensively due to high anatomical and physiological resemblances to humans in comparison to other non-rodent species. GMPs may help bridge the gap between initial findings in rodents before human clinical trials [11–13]. The incidence of epilepsy in a small subset of GMPs with a well-described genetic background provides a promising platform for the establishment of an animal model for characterising epilepsy and better understanding the mechanisms behind the disease in humans. Thus, this study aimed to identify genomic and transcriptomic correlates to human epilepsy in epileptic GMPs.

We identified numerous known epilepsy and seizure genes as well as novel candidate genes by comparing whole genomes of healthy with seizure-phenotype GMPs. Furthermore, we identified genes that were differentially expressed (DE) in primary skin fibroblasts isolated from epileptic and healthy GMPs and correlated these genes to mutations that were close to fixation in the genome of epileptic GMPs. Using this approach, we discovered numerous epilepsy candidate genes, e.g. *CACNA1H*. Furthermore, we identified the transcription factors *EGR3* and *HOXB6* as likely candidate regulators of *CACNA1H* expression. We provide the first insight into the underlying basis of the epileptic phenotype of GMPs and identified new candidate genes for validation in human epilepsy. This study is the first step toward establishing GMPs as a model system for human epilepsy.

## Material and methods

### Spontaneous seizure behaviour

All GMPs were born and housed in dedicated barrier facilities (Ellegaard Göttingen Minipigs A/S). Health monitoring of the animals at the facility is performed according to the Federation of European Laboratory Animal Science Associations (FELASA). Recommendations of Best Practices for the Health Management of Ruminants and Pigs used for Scientific and Educational Purposes (2020) were followed and documented biannually. Animals were group housed, when possible, in pens with bedding material (irradiated straw) and biting nipples as environmental enrichment. Animals were fed twice daily and water was offered ad libitum. Six GMPs found to display intermittent spontaneous seizures were placed under surveillance in dedicated barrier facilities (Ellegaard Göttingen Minipigs A/S, Denmark). Abnormal behaviours as described below were logged as spontaneous seizure events. Epileptiform behaviours were observed as involuntary movements of the body and/or head, which were further defined as repeated head throwing, masticatory movement without food/abrupt jaw movements, falling to the ground, losing a step, excessive eye blinking, backing, abnormal gait, opisthotonos/hyperextension of the neck, paddling, and tonic and/or clonic convulsions. Information about age, detected age of onset of the seizure phenotype, and seizure durations of animals that were used for primary fibroblast culture is summarised in Supplementary Information S1. No randomisation was performed as this was an observational study. All investigators were aware of group designation in the study.

### Tissue sample collection

Skin biopsies (2 cm^2^) were taken immediately post-mortem from the ventral flank area of 6 epileptic GMPs (3 females/3 males, at the age of 5–11 months) and 3 non-epileptic control animals (males, 31–36 months). The total sample size was 9, with no power calculations as this was a case study. Biopsies were washed gently with gauze swabs soaked in normal saline and then 70% ethanol to remove any debris, then rinsed with phosphate-buffered saline (PBS) and immediately placed in a 15-ml tube filled with Dulbecco’s Modified Eagle Medium (DMEM), 10% foetal bovine serum (FBS), and 1% Penicillin–Streptomycin (Pen/Strep). Tubes were transported to the laboratory on ice.

### Isolation of primary fibroblasts

Skin biopsies were prepared and cultured under a modified protocol [14]. In brief, the skin tissue was washed five times with PBS, soaked once in 70% ethanol for 5 min, then allowed to dry. Biopsy was transferred into a 100-mm tissue culture dish containing 10 ml DMEM medium and cut into pieces smaller than 3 mm in diameter and then digested in a mix of 10 mg collagenase D (0.153 U/mg) in 4 ml complete medium and 10 mg Pronase (50 U/mg) for 90 min. After incubation, the digested tissue was passed through a 70-µm cell strainer and the cell suspensions were centrifuged for 7 min at ~ 600 × g. The supernatant was removed, and the cell pellet was resuspended in a 5 ml DMEM medium and added to a gelatine-coated T25 flask. Cells were incubated at 37 °C at 95% relative humidity and 5% CO_2_. On the third day, the medium was replaced with 5 ml fresh medium. When the primary fibroblasts were confluent enough for passaging, they were detached by adding 1 ml accutase and incubated for 5 min, then resuspended in 2 ml medium and transferred to the 15-ml tube for 5 min centrifuging at 400 × g. The cell pellet was resuspended in DMEM medium and passaged to a gelatine-coated T75 flask. The fibroblast cells were passaged again after 4 days and then the cell pellet was used for further processing.

### RNA isolation from fibroblast cells

RNA isolation from primary fibroblasts was performed for RNA sequencing. RNA was isolated using the RNeasy Mini kit (Qiagen, Valencia, CA, USA). For RNeasy Mini kit RNA isolation, cells were lysed using 350 μl of buffer RLT (supplied in kit), then placed into QIAshredder homogenizer (Qiagen) and centrifuged at ~ 580 × g for 2 min. 350 μl of 70% ethanol was added to the flow-through, mixed, and centrifuged in the RNeasy Mini column (supplied in kit) for 15 s at 8000 × g. The flow-through was discarded, and the column was washed with 700 μl of buffer RW1 (supplied in the kit) for 15 s at 8000 × g. Two additional washes were performed with 500 μl of buffer RPE (supplied in kit) at 8000 × g for 2 min and 15 s, respectively. The flow-through was discarded, and the columns were placed in a sterile 2-ml collection tube. Depending on the expected yield, 20–50 μl RNase-free water was pipetted into the column and centrifuged for 1 min at 8000 × g. The RNA yield was measured (A260/A280) using a Tecan Infinite F200 microplate reader (Tecan, Zürich, Switzerland) and isolated RNA were stored at − 80 °C until further analysis.

### NGS library preparation and sequencing

RNA sequencing was performed as a service by Eurofins Genomics Europe Sequencing GmbH, which included strand-specific cDNA library construction, sequencing on Illumina NovaSeq 6000 S4 PE150 XP (2 × 150 bp reads), and demultiplexing.

For DNA sequencing, blood samples of animals showing spontaneous convulsive seizures were taken from 22 (13 females, 9 males) GMPs born at Ellegaard Göttingen Minipigs A/S in 2015 and 2016. DNA was extracted and pooled with equimolar quantities. Four DNA pools made up of ten clinically healthy companions each were used as control. The latter is identical to the pools DA2_1, DA2_2, DA3_1, and DA3_2 [15]. All pools were paired-end sequenced with an Illumina HiSeq 4000 to an aim depth of coverage of 30 × , 150 bp read length, and roughly 420 bp insert size.

### Comparative genomic analyses, variant calling, and FST analysis

Raw resequencing data were aligned to the reference genome SScrofa11.1 [16] and subsequently processed with a GATK pipeline as described in [15]. Alternative allele frequencies in each pool $k$ were estimated as ${p}_{k}=\frac{{R}_{alt}}{{R}_{ref}+{R}_{alt}}$, with ${R}_{ref;alt}$ referring to number of alleles supporting reads, derived from the allelic depth values from the VCF. ${F}_{ST}$ was estimated for all variable loci with successful call in the seizure pool and at least one control pool as ${F}_{ST}=\frac{\overline{p }*\left(1-\overline{p }\right)-\frac{{n}_{SE}*{p}_{SE}*\left(1-{p}_{SE}\right)+{n}_{CO}*{p}_{CO}*\left(1-{p}_{CO}\right)}{{n}_{SE}+{n}_{CO}}}{\overline{p }*\left(1-\overline{p }\right)}$, with $\overline{p }=\frac{{n}_{SE}*{p}_{SE}+{n}_{CO}*{p}_{CO}}{{n}_{SE}*{n}_{CO}}$. Subscripts $SE$ and $CO$ indicate either the seizure or the control pool, which is a virtual pool of the healthy pools successfully genotyped for the respective position. Accordingly, allele frequencies are weighted with ${n}_{SE}$ being 22, ${n}_{CO}$ being the number of successfully called pools (up to 4) with 10 (individuals each), and ${p}_{CO}$ being the average alternative allele frequency of the control pools.${F}_{ST}$ results were intersected with the positional data from DEGs which were extended by 200 kbp to both sides.

### Transcriptome analyses

Quality control and trimming of raw sequencing reads were performed with Trimmomatic version 0.36 (settings: PE -phred33 LEADING:3 TRAILING:3 SLIDINGWINDOW:4:15 MINLEN:36) [17]. Reads were aligned to the *Sus scrofa* reference genome version GCF_000003025.6 (see Availability of data and materials) using HiSat2 version 2.1.0 with default settings [18]. Splice sites were derived from the Gene transfer format (GTF) file (see Availability of data and materials). The R packages Genomic Features (Version 1.46.5) and summarize Overlaps were used to count exon spanning reads [19]. DE analyses were conducted with DESeq2 (Version 1.34.0) [20]. The R package Enhanced Volcano (Version 1.12.0) was used to generate volcano plots of DE results.

### Functional analyses

Variant effect prediction was performed with the Ensembl Variant Effect Predictor web interface [21] with all variants that exceeded the threshold FST > 0.9 using the default settings with an Upstream/Downstream distance of 5 kb. Gene set enrichment analyses were performed with STRING (Version 12.0) [22]. Gene cluster comparison and visualization were achieved with the R package clusterProfiler (Version 4.2.2) [23]. Gene symbols were converted to ensemble IDs with the clusterProfiler Biological Id Translator (bitr). GO term analyses were performed with enrichGO (settings: pAdjustMethod = “fdr”, pvalueCutoff = 1, qvalueCutoff = 0.25, readable = TRUE, minGSSize = 10). KEGG pathway analysis was done with enrichKEGG (settings: pvalueCutoff = 1, pAdjustMethod = “BH”, minGSSize = 10, maxGSSize = 500, qvalueCutoff = 0.25, use_internal_data = FALSE). Plots were created with the dotplot function.

### Gene network construction and transcription factor binding site enrichment analysis

Co-regulated genes were identified with the dGCR (Database of Gene Co-regulation) web tool [24] using the default settings. The output of dGCR was filtered to only contain DEGs (abs. Log_2_FC > 1, *p*-value < 0.01). Gene networks with less than 4 nodes were discarded. Graphical representation of the gene network was created with Cytoscape (Version 3.9.1) [25] and gene classification was performed with the PANTHER classification system [26]. Transcription factor binding site enrichment analysis was performed with Ciiider (build May 15th, 2020) [27] using the following position frequency matrices (PFMs), which were acquired from the 9th release (2022) of JASPAR [28]: MA1500.1 (*HOXB6*), MA0904.2 (*HOXB5*), MA0732.1 (*EGR3*), MA0904.1 (*HOXB5*), and MA0905.1 (*HOXC10*). Background genes with an abs. Log_2_FC < 0.5 were selected, which resulted in a set of 11,294 genes. The *p*-value threshold for gene coverage enrichment was set to 0.05, the base position upstream scan limit to 1500 bp, and the base position downstream scan limit to 500 bp.

## Results

**Fig. 1:**
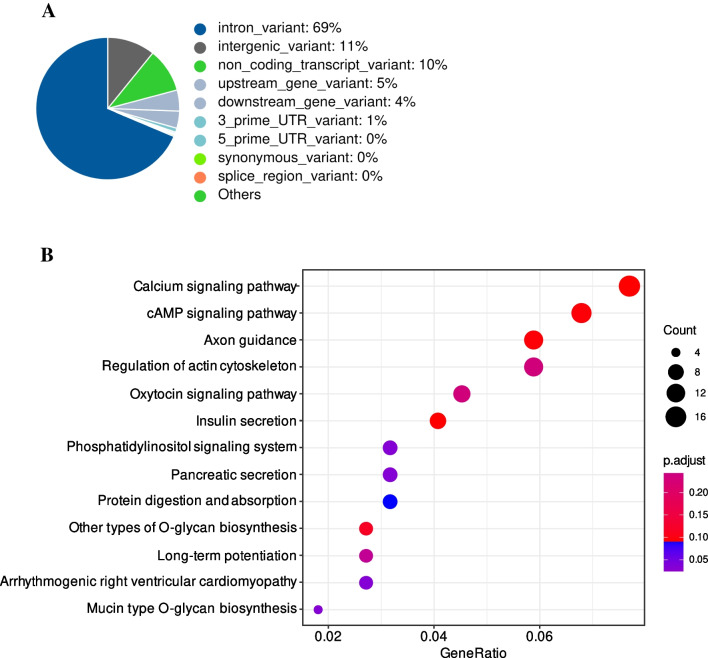
**A** Summary of variant effect prediction of highly differentiated genetic variants between healthy and seizure-phenotype GMPs. **B** KEGG pathway gene clustering results of genes in close proximity (± 5 kbp) to fixed variants between healthy and seizure-phenotype GMPs

To identify causative genetic variants for epileptic seizures in GMPs, WGS of pooled animals was performed. Variant calling in the genomic sequence data of seizure and control pools resulted in the discovery of 15.8 M SNP variants with variability within GMPs. Of these variants, 1276 showed outstanding differentiation with an ${F}_{ST}$ ≥ 0.95 between healthy and epileptic GMPs (Supplementary Information S2). Variant effect prediction revealed that 628 genes were located in close proximity (± 5 kbp) to those genes (Fig. 1A, Supplementary Information S3). Gene set enrichment analysis (GSEA) results are shown in Table 1 (complete results in Supplementary Information S4).

**Table 1:** Gene set enrichment analysis results with STRING. The top five results of each category are shown

Category	Term ID	Term description	Strength	FDR
GO Process	GO:0099504	Synaptic vesicle cycle	0.71	0.0118
GO Process	GO:0007156	Homophilic cell adhesion via plasma membrane adhesion molecules	0.65	0.0249
GO Process	GO:0001505	Regulation of neurotransmitter levels	0.64	0.0033
GO Process	GO:0006836	Neurotransmitter transport	0.64	0.0439
GO Process	GO:0098742	Cell–cell adhesion via plasma membrane adhesion molecules	0.6	0.0067
GO Function	GO:0099604	Ligand-gated calcium channel activity	1.04	0.0243
GO Function	GO:0008376	Acetylgalactosaminyltransferase activity	0.88	0.0266
GO Function	GO:0005516	Calmodulin binding	0.69	0.00099
GO Function	GO:0004725	Protein tyrosine phosphatase activity	0.64	0.0404
GO Function	GO:0016758	Hexosyltransferase activity	0.49	0.0467
GO Component	GO:0031901	Early endosome membrane	0.68	0.006
GO Component	GO:0030027	Lamellipodium	0.64	0.0013
GO Component	GO:0030175	Filopodium	0.64	0.0483
GO Component	GO:0098978	Glutamatergic synapse	0.49	0.0188
GO Component	GO:0031252	Cell leading edge	0.44	0.0138
TISSUES	BTO:0000227	Central nervous system	0.23	0.0000342
TISSUES	BTO:0001484	Nervous system	0.23	0.0000342
TISSUES	BTO:0000142	Brain	0.23	0.0000697
TISSUES	BTO:0000282	Head	0.2	0.0000785
TISSUES	BTO:0000042	Animal	0.09	0.000000024
COMPARTMENTS	GOCC:0030054	Cell junction	0.38	0.002
COMPARTMENTS	GOCC:0045202	Synapse	0.43	0.0093

In the category *TISSUES*, the top four terms were “Central nervous system”, “Nervous system”, “Brain”, and “Head”, which verify that the discovered variants mainly affect tissues that are relevant for the seizure phenotype under investigation. Regarding Gene Ontology Processes and Functions terms related to the synapse, neurotransmitters and calcium channels were found, which also fit the phenotype. Gene clustering results for KEGG pathways are shown in Fig. 1B. The KEGG term with the highest significance detected was “calcium signalling pathway”, which included the genes *ADORA2B*, *CACNA1E*, *CAMK1D*, *CAMK1G*, *CAMK2A*, *CHRM2*, *GRIN2B*, *ITPKB*, *LOC100154782*, *MCOLN2*, *MYLK*, *NFATC3*, *PDGFD*, *PHKB*, *PPP3CC*, *RYR2*, and *VDAC1*. Since epilepsy is a disease associated with ion channel dysfunction, these genes have a high probability to being causative for the seizure phenotype under investigation in this study.

**Fig. 2:**
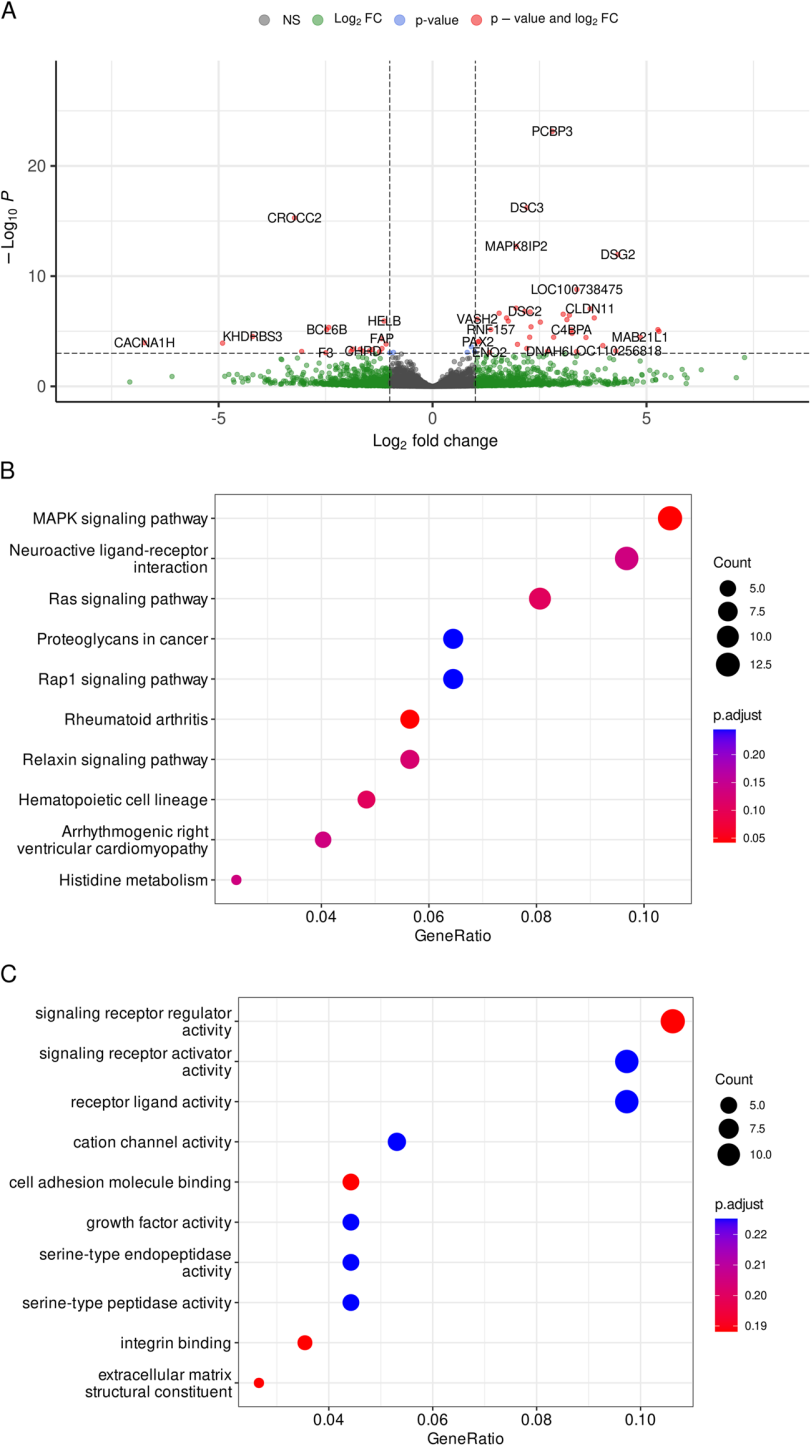
**A** Volcano plots of DEGs in primary fibroblasts isolated from healthy and seizure-phenotype GMPs. **B** KEGG pathway gene clustering results and **C** GO molecular functions gene clustering results from DEGs between fibroblasts isolated from healthy and seizure-phenotype GMPs

To validate the candidate mutations from the genomic analyses, we compared the primary skin fibroblast transcriptomes from three healthy minipigs against six animals that had developed a spontaneous seizure phenotype. Fibroblasts have a high predictive diagnostic power over other minimally invasive tissue samples [29]. For this, RNA was isolated from primary cultured fibroblasts established from each animal. The average overall alignment rate was 97.9% (SD = 0.4%), which resulted in an average exon coverage of 114 × (SD = 22) and a transcriptome coverage of 45 × (SD = 8.6). Differential expression analysis led to the discovery of 56 differentially expressed genes (DEGs) (abs. Log_2_ fold change (Log_2_FC) > 1, *p-*adj. < 0.001, Fig. 2A). The complete results of the differential expression analysis are summarised in Supplementary Information S5. Of the highly differentiated variants, 27 were in proximity (± 200 kbp) to the 56 DEGs (Table 2, Supplementary Information S6). As such, DEGs with extended boundaries cover approximately 26.2 Mbp, which is about 1% of the assembled chromosome length, only 14 highly differentiated SNPs are expected under the assumption of random distribution, leading to a 1.9-fold enrichment of highly differentiated SNPs around DEGs.

**Table 2:** Differentially expressed genes with mutations that were close to fixation in GMPs within a maximum distance of 200 kbp

Position	Gene_ID	Symbol	Ref	Alt	Gene product	Consequence	Distance (bp)	Log_2_FC	*p-*adj
1:142,630,415	100155343	*MKRN3*	G	A	Makorin ring finger protein 3	upstream_gene_variant	129,446	5.29	1.03 × 10^−5^
3:40,590,098	110259950	*CACNA1H*	A	G	Ca^2+^ voltage-gated channel subunit alpha1 H	downstream_gene_variant	47,323	− 6.73	1.10 × 10^−4^
4:6,186,373	100155706	*KHDRBS3*	A	G	KH RNA-binding domain containing signal transduction associated 3	downstream_gene_variant	103,205	− 4.21	2.95 × 10^−5^
4:6,270,027	100155706	*KHDRBS3*	G	T	KH RNA-binding domain containing signal transduction associated 3	downstream_gene_variant	19,551	− 4.21	2.95 × 10^−5^
4:27,970,032	100157483	*SYBU*	C	T	Syntabulin	intron_variant	/	− 2.43	4.50 × 10^−6^
4:122,811,247	396677	*F3*	A	G	Coagulation factor III, tissue factor	upstream_gene_variant	15,774	− 2.50	9.01 × 10^−4^
4:122,811,311	396677	*F3*	T	G	Coagulation factor III, tissue factor	upstream_gene_variant	15,708	− 2.50	9.01 × 10^−4^
4:122,811,350	396677	*F3*	C	T	Coagulation factor III, tissue factor	upstream_gene_variant	15,671	− 2.50	9.01 × 10^−4^
4:122,811,369	396677	*F3*	A	G	Coagulation factor III, tissue factor	upstream_gene_variant	15,652	− 2.50	9.01 × 10^−4^
4:122,811,392	396677	*F3*	C	G	Coagulation factor III, tissue factor	upstream_gene_variant	15,629	− 2.50	9.01 × 10^−4^
5:30,751,306	100514840	*HELB*	C	A	DNA helicase B	downstream_gene_variant	54,817	− 1.13	1.18 × 10^−6^
5:63,737,977	100157750	*ENO2*	G	C	Enolase 2	downstream_gene_variant	61,951	1.33	8.25 × 10^−4^
6:2,738,830	106510455	*LOC106510455*	C	T	ncRNA	downstream_gene_variant	103,866	3.98	2.00 × 10^−4^
6:2,738,833	106510455	*LOC106510455*	G	A	ncRNA	downstream_gene_variant	103,863	3.98	2.00 × 10^−4^
6:2,912,893	106510455	*LOC106510455*	C	A	ncRNA	upstream_gene_variant	60,155	3.98	2.00 × 10^−4^
9:1,044,237	100520142	*RIC3*	C	T	RIC3 acetylcholine receptor chaperone	upstream_gene_variant	90,679	− 1.09	1.44 × 10^−4^
12:5,426,670	100522340	*RNF157*	T	C	Ring finger protein 157	downstream_gene_variant	120,472	1.36	7.13 × 10^−6^
12:24,989,432	100522700	*HOXB5*	C	G	Homeobox protein Hox-B5	upstream_gene_variant	139,792	3.20	3.47 × 10^−7^
12:24,989,432	100627849	*HOXB6*	C	G	Homeobox protein Hox-B6	upstream_gene_variant	135,937	1.73	6.16 × 10^−7^
12:24,989,432	102166259	*LOC102166259*	C	G	ncRNA	upstream_gene_variant	150,705	1.98	1.54 × 10^−4^
12:24,989,457	100522700	*HOXB5*	G	T	Homeobox protein Hox-B5	upstream_gene_variant	139,817	3.20	3.47 × 10^−7^
12:24,989,457	100627849	*HOXB6*	G	T	Homeobox protein Hox-B6	upstream_gene_variant	135,962	1.73	6.16 × 10^−7^
12:24,989,457	102166259	*LOC102166259*	G	T	ncRNA	upstream_gene_variant	150,730	1.98	1.54 × 10^−4^
12:59,436,738	102158777	*SPECC1*	G	A	Sperm antigen with calponin homology and coiled-coil domains 1	downstream_gene_variant	30,041	1.09	8.15 × 10^−5^
14:82,086,114	100524960	*DYDC1*	A	T	DPY30 domain containing 1	downstream_gene_variant	90,891	3.59	3.68 × 10^−5^
15:34,150,428	106506202	*LOC106506202*	G	A	Uncharacterized protein	intron_variant	/	2.27	3.47 × 10^−5^

Gene cluster enrichment analysis was performed with DEGs (abs. Log_2_FC > 1, *p*-value < 0.01) and we discovered the top KEGG terms affected in the comparison between healthy and seizure pigs were “MAPK signalling pathway”, “Neuroactive ligand-receptor interaction”, and “Ras signalling pathway” (Fig. 2B). Enriched GO molecular functions were largely related to receptor and channel activities (Fig. 2C). The complete dataset of gene clustering results is summarised in Supplementary Information S4.

**Fig. 3:**
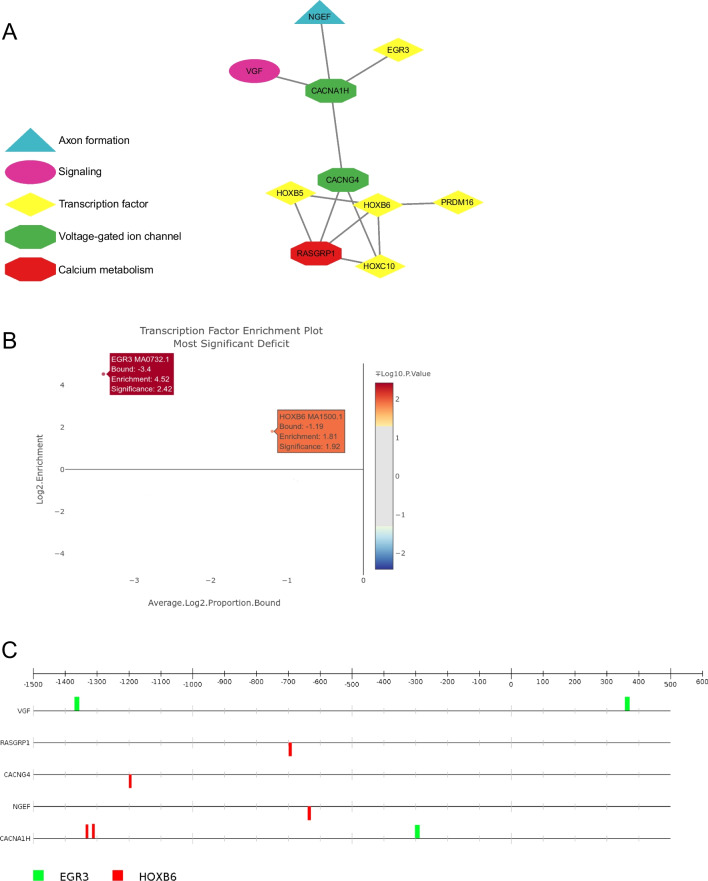
**A** Core regulatory network of DEGs in proximity to fixed genetic variants. **B** Transcription factor binding site enrichment results of the core network genes performed with the transcription factors in the network. **C** Positions of transcription factor binding sites in DEGs of the core regulatory network

The dGCR web tool [24] was utilised to discover genes, which are co-regulated with the DEGs in proximity to a fixed mutation. The output of dGCR was compared with our list of DEGs (abs. Log_2_FC > 1, *p*-value < 0.01) and this resulted in the discovery of one co-regulated gene network with more than 3 nodes (Fig. 3A). This network included the two voltage-gated ion channels *CACNA1H* and *CACNG4*, the Ca^2+^ metabolism protein *RASGRP1*, and *NGEF*, which is involved in axon formation, and the signalling molecule *VGF* [30, 31]. Furthermore, the five transcription factors *EGR3*, *HOXB5*, *HOXB6*, *HOXC10*, and *PRDM16* are part of the network. Transcription factor binding site enrichment analysis with Ciiider revealed that binding sites for the transcription factors *EGR3* (Log_2_ enrichment = 4.52, *p-*value = 0.004) and *HOXB6* (Log_2_ enrichment = 1.81, *p-*value = 0.012) are significantly enriched in proximity (1.5 kb upstream, 0.5 kb downstream) to the genes encoding for the proteins in that co-regulated network (Fig. 3B). *CACNA1H* is exceptional in this regard since it contains predicted binding sites for both transcription factors (Fig. 3C). Since *CACNA1H* is the most downregulated gene among DEGs (Log_2_FC =  − 6.734), we conclude that the downregulated *EGR3* (Log_2_FC =  − 2.147) is an activator of *CACNA1H* expression and that the upregulated *HOXB6* (Log_2_FC = 1.728) is a repressor of *CACNA1H*. The complete transcription factor binding site enrichment results are summarised in Supplementary Information S7.

## Discussion

Epilepsy has a significant genetic component, with research suggesting that genetic factors may account for up to 80% of the risk of developing the condition. While specific genes and genetic variations associated with epilepsy have been identified, the inheritance pattern is often complex and multifactorial. Currently, available animal models for epilepsy do not recapitulate the diverse aspects of epilepsy [32]. The identification of the rare occurrence of GMPs with high recurrent spontaneous seizures offers a unique opportunity for the establishment of a pig model of epilepsy-type disease, both to better understand disease pathogenesis and in the development of new treatments. The advantages of a GMP model for epilepsy include the well-described genetic background and controlled breeding in an established breeding program. In addition, their high health status and microbiological definition decrease the risk of confounders and unwanted variables. The large and complex gyrencephalic brain of pigs has more similarity to the human brain than those of simpler model organisms and provides the potential for a better understanding of the genetic aspects of epilepsy [33]. Unpublished data based on pedigrees of affected animals suggest a moderate to high heritability and recessive mode of inheritance. Hence, the aim of this study was to elucidate if GMPs with a seizure phenotype have commonalities at the genome and transcriptome level with human epileptic disorders. We compared the genomes and transcriptomes of two experimental groups of healthy and seizure-phenotype GMPs to discover the seizure-associated genes. Variant calling from whole genome sequences led to the identification of numerous genes that were enriched in GO terms, which link them to the synapse, neurotransmitters, and calcium channel activity. Furthermore, 17 genes clustered in the KEGG “calcium signalling pathway” (Table 3). About half of those genes have been previously linked to epilepsy or seizures. This is a clear indicator that GMPs are suitable as a reliable and robust model system for human epilepsy. The remaining genes should be considered novel epilepsy candidate genes and they were mostly linked to other neurological disorders.

**Table 3:** Genes within close proximity to highly differentiated genetic variants between healthy and seizure-phenotype GMPs, which belong to the KEGG “calcium signalling pathway”. Studies linking these genes to epilepsy, seizures, or other neurological disorders are indicated

Gene	Gene product	Associated neurological disorders
*ADORA2B*	Adenosine receptor A2b	Chronic pain [34]
*CACNA1E*	Voltage-dependent R-type calcium channel subunit alpha-1E	Epilepsy [35]
*CAMK1D*	Calcium/calmodulin-dependent protein kinase type 1D	Alzheimer's disease [36]
*CAMK1G*	Calcium/calmodulin-dependent protein kinase type 1G	Epilepsy [37]
*CAMK2A*	Calcium/calmodulin-dependent protein kinase type II subunit alpha	Seizures [38]
*CHRM2*	Muscarinic acetylcholine receptor M2	Epilepsy [39]
*GRIN2B*	Glutamate receptor ionotropic, NMDA 2B	Seizures [40]
*ITPKB*	Inositol-trisphosphate 3-kinase B	Parkinson’s disease [41]
*LOC100154782*	5-hydroxytryptamine receptor 5B	
*MCOLN2*	Mucolipin-2	
*MYLK*	Myosin light chain kinase, smooth muscle	Intracranial aneurysm [42]
*NFATC3*	Nuclear factor of activated T-cells, cytoplasmic 3	Excitotoxic and raumatic brain insults [43]
*PDGFD*	Platelet-derived growth factor D	Stroke [44]
*PHKB*	Phosphorylase b kinase regulatory subunit beta	Pediatric glycogen storage disease [45]
*PPP3CC*	Serine/threonine-protein phosphatase 2B catalytic subunit gamma isoform	Epilepsy [46]
*RYR2*	Ryanodine receptor 2	Epilepsy [47]
*VDAC1*	Voltage-dependent anion-selective channel protein 1	Epilepsy [48]

Among genes, which were not linked to neuropathology thus far, is the Ca^2+^-permeable cation channel *MCOLN2* (synonym *TRPML2*). Constitutive activity of *MCOLN2* induces cell degeneration [49] and might play a role in the irreversible brain damage and cognitive decline associated with frequent and recurrent epileptic seizures. *LOC100154782* (5-hydroxytryptamine receptor 5B) is a 5-HT and serotonin receptor. 5-HT receptors are under discussion to be novel epilepsy treatment targets. [50]. The gene *Htr5b* is expressed in mice and rats, but not in humans, where the open reading frame is disrupted by stop codons [51]. Hence, it might play a role in seizures in animals but not in humans.

The remaining genes from Table 3 are involved in other neurological conditions and are therefore interesting epilepsy candidate genes. In particular the transcription factor *NFATC3*, since it was linked to excitotoxic and traumatic brain insults [43]. Excitotoxic damage is a hallmark in epilepsy and has been attributed to glutamatergic mechanisms [52]. Nuclear NFATC3 accumulation in pericytes depends on neuronal activity and the activation of group I metabotropic glutamate receptor [53]. In summary, this evidence makes *NFATC3* a promising epilepsy candidate gene. The *ITPKB* gene protects against α-synuclein aggregation in Parkinson’s disease [41] and high cytoplasmic expression of α-synuclein was detected in brains of rats with induced experimental epilepsy [54]. A connection between the two observations should be further investigated.

To investigate the functional impact of the detected variants on gene expression, transcriptome analyses were performed. Since only primary fibroblasts were available as a source for RNA at the time, only DEGs with a highly differentiated genetic variant in proximity were considered relevant. Among the highest DEGs with fixed genetics variants in proximity, we found *CACNA1H* and *SPECC1*, which have previously been linked to epilepsy-type disorders in humans [8, 55, 56]. A wide range of epileptic disorders in humans have been related to dysregulation of the electrophysiological properties of ion channels and neurotransmitter systems, known as idiopathic generalised epilepsy (IGE). Ion channels are pore-forming proteins that allow ions to pass through the cell membrane [57, 58]. Voltage-gated Ca^2+^ channels lead to the conduction of inward Ca^2+^ currents after depolarization, which mediates the firing of an action potential [3]. These channels can contribute to the development of seizures by mediating the conduction of a Ca^2+^ current after depolarization. Thus, targeting these channels is a potential strategy for new treatments. Understanding the specific role of voltage-gated Ca^2+^ channels in epilepsy is important for the development of targeted therapies.

The role of voltage-gated channels in the activity of neuronal excitability indicates that mutations in Ca^2+^ channel genes *CACNA1A*, *CACNA1H*, *CACNA2D2*, and *CACNB4* might be associated with the occurrence of epilepsy [55]. *CACNA1H* encodes the α1 subunit of Ca_V_3.2, which is a Ca_V_3 subfamily member. Ca_V_3 channels are mainly expressed in thalamic neurons and have a main role in the conduction of low-voltage activated T-type Ca^2+^ currents [8]. Nelson et al. showed that *CACNA1H* mutations disrupt the function of Ca^2+^ channels and may lead to an increase in synchronously firing neurons [59]. An increase in the synchronous firing of neurons leads to neural hyperexcitability and the occurrence of seizures. It should be pointed out that the unusual activity of neurons is referred normally to its onset origin, but during the occurrence of seizures, it could spread to other parts of the brain, leading to extensive abnormalities in the functionality of the brain [59]. Our findings are consistent with previous studies and strongly indicate the role of *CACNA1H* as a potential gene in IGE. Khosravani and Zamponi demonstrated that *CACNA1H* mutations and their dysfunctional regulations act interactively with many factors like other cation channels and transcription factors, which lead to many abnormal activities in the epileptic brain [8]. Another DEG identified in our study is *SPECC1*. Two probands with idiopathic generalised epilepsy carried deletions in *SPECC1*. Therefore, we suggest that *SPECC1* could be a potential novel candidate gene for epilepsy [56]. By conducting gene cluster enrichment analysis with DEGs, we identified two key pathways: mitogen-activated protein kinases (MAPK) and neuroactive ligand-receptor interaction pathway. Our data indicate that the most significant DEGs were particularly enriched in MAPK signalling pathways, such as *CACNA1H* and *TNF*, which are involved in generalised epilepsy [60]. Notably, the mTOR/MAPK signalling pathway regulates RNA-binding proteins (RBPs), and then influences the mRNA expression encoding target markers of epilepsy. Thereby, misregulated mTOR/MAPK-RBPs interaction may lead to the excessive synthesis of ion channels and their receptors, resulting in hyperexcitability of the cells [58]. Accordingly, if the stimulation of synapses is continuous, this leads to further activation of the mTOR/MAPK pathway through RBPs expression and provides a basis for the occurrence of epilepsy [61]. In general, one important aspect of epileptogenesis in all epileptic animal models studied so far is dysregulation of synaptic function, which includes the dysfunction of ion channel expression, presynaptic and postsynaptic neurotransmitters, and their receptor expression. Although in this study, we have not investigated RBP expression, previous studies have demonstrated that ion channels and their receptors might be controlled by RBPs [62]. For instance, Ferron et al. indicated that FMRP as an RNA-binding protein regulates the localization of voltage-gated Ca^2+^ channels to the presynaptic nerve terminal. Therefore, it might be expected that any inopportune translation of synaptically synthesised proteins involved in excitability could lead to epilepsy [31].

We also identified the KEGG pathway neuroactive ligand-receptor interaction, consisting of DEGs encoding neuroreceptors, such as *GRIA4*, which is significantly associated with a molecular mechanism involved in temporal epilepsy [63]. Indeed, a mutation in the *GRIA4* gene, which encodes an AMPA receptor (AMPA-R) subunit expressed in cortical as well as thalamic neurons, leads to the absence of seizures [64]. AMPA-R are ionotropic glutamate receptor subtypes that are coupled to ion channels and regulate fast cell excitability by controlling the Na^+^ and Ca^2+^ ion influx throughout the CNS [64, 65]. Another important finding in our study is the identification of the Ras signalling pathway as a possible component of epilepsy. The Ras pathway plays different roles in neuroplasticity. Mignot et al. indicated that the over activation of Ras is often related to neurological disease [66], which is consistent with the current study. Our finding showed Ras signalling in epilepsy includes the DEGs *RASGRP3* and *SYNGAP1. SYNGAP1* encodes the RAS-GTPase-activating protein. It is interesting to note that this gene has a critical function in the density regulation of NMDA and AMPA receptors at the glutamatergic synapse and mediates the activation of glutamate receptors [67]. Some studies have demonstrated that mutation in *SYNGAP1* can be also associated with generalised epilepsy in humans [66, 68].

Most of the GO molecular function enrichment analyses in the current study indicated epilepsy-related genes in cation channel activity and signalling ligand receptors. Among the possible target candidates, we found the DEG *KCNAB1* which belongs to the voltage gate channel group associated with epilepsy [69, 70]. Previous studies demonstrated the involvement of potassium channels in some familial epileptic disorders [71]. Moreover, potassium voltage gate channels are mainly located in the presynaptic area and include two pore-forming α-subunits and one β-subunit (Kvβ1.1), which occludes the channel pore after depolarization and acts as a regulator in channel deactivation. Our findings are consistent with other studies where intronic variants in *KCNAB1*, which encodes Kvβ1.1, were associated with lateral temporal epilepsy [69, 72].

We discovered one co-regulated gene network (Fig. 3A) which includes the Ca^2+^ voltage-gated ion channels *CACNA1H* and *CACNG4* and some transcription factors: *HOXB5*, *HOXB6*, *HOXC10*, and *EGR3*. Among the discovered transcription factors, binding sites for *EGR3* and *HOXB6* are significantly enriched near *CACNA1H* (Fig. 3C). Our results suggest that the downregulated *EGR3* acts as an activator of *CACNA1H* expression. *EGR3* encodes a transcriptional regulatory factor belonging to the EGR family [73], which participates in the regulation of dendritic morphology in sympathetic neurons and branch formation of terminal axons. This process is crucial for the normal development of the sympathetic nervous system [30]. Procedures that lead to neuronal hyperexcitability, such as seizures, lead to *EGR* gene overexpression [74]. *EGR3* expression is elevated in the hippocampus in human and animal models with temporal lobe epilepsy [75]. It has been previously reported that *EGR3* upregulates *GABRA4* promoter activity and endogenous expression level of GABA_A_ receptor α4 subunits in response to a seizure-induced status [75]. Transcription factor binding site enrichment analysis revealed *EGR3* binding sites upstream of *CACNA1H*. *EGR1* binding sites are significantly overrepresented in the Ca_V_3.2 promoter region and regulate transcription of the *CACNA1H* gene, which encodes T-type calcium channel Ca_V_3.2, contributing to the development of neuronal hyperexcitability in epileptogenesis [6]. Although we have found significant enrichment of transcription factor binding sites for *EGR3*, sequence-specific DNA binding, transcription activities, protein structure aspects, and *EGR3* regulation have many similarities to *EGR1* [76]. However, comprehending the key mechanisms of controlling the Ca_V_3.2 gene expression is still limited. Therefore, further experiments, such as expression of transcription factors in a specific region of the brain such as the cortex, will help to elucidate the roles of particular genes involved in epilepsy. Furthermore, we assume that upregulated *HOXB6* acts as a repressor of *CACNA1H* expression*.* Transcriptomic studies demonstrate that HOX family transcription factors are mainly needed for synapse formation and also neuronal network maturation in the central nervous system (CNS) [77, 78]. To our knowledge, HOX genes were not linked to epilepsy yet.

The genomic and transcriptomic analyses of seizure-phenotype GMPs in comparison to healthy GMPs validated numerous known epilepsy and seizure genes and revealed novel candidate genes. The study presented here is the first step toward a robust and reliable non-rodent model system for human epilepsy and warrants further experimental characterisation. Transcriptomic analyses of cultured fibroblasts in three healthy and six epileptic seizure minipigs have revealed a potential role for differential expression of *CACNA1H* in epilepsy. Previous studies have shown that mutations in *CACNA1H* disrupt Ca^2+^ voltage-gated channels, leading to neural hyperexcitability and seizure occurrence. We also found the MAPK signalling pathway, neuroactive ligand-receptor interaction, and cation signalling pathways previously described in human epilepsy are dysregulated in GMPs experiencing spontaneous, recurrent seizures. Furthermore, our results pointed to the involvement of two transcription factors, *EGR3* and *HOXB6*, as potential key regulators of *CACNA1H* and *CACNAG4* expression*,* as well as the involvement of HOX genes, which may point to an early developmental disorder in GMPs.

### Supplementary Information

Below is the link to the electronic supplementary material.

Supplementary file1 (XLSX 13 KB)

Supplementary file2 (XLSX 38 KB)

Supplementary file3 (XLSX 502 KB)

Supplementary file4 (XLSX 74 KB)

Supplementary file5 (XLSX 1890 KB)

Supplementary file6 (PDF 8068 KB)

Supplementary file7 (XLSX 7 KB)

## Data Availability

The short read data of the DNA pools is available in the European Nucleotide Archive (ENA) via BioProject accession number PRJEB36673. The data of seizure minipigs is available via Accession number ERS18230258. The short read data of the RNA sequencing data is available via accession number PRJNA1000481.
