# Supplementary material for: Genomic evidence for the suitability of Göttingen Minipigs with a rare seizure phenotype as a model for human epilepsy
Source: Neurogenetics. 2024 Feb 21;25(2):103–17. doi: 10.1007/s10048-024-00750-2 (PMC11076379; doi:10.1007/s10048-024-00750-2)

Supplementary Information S6: Graphical FST analysis results for genes, which were differentially expressed

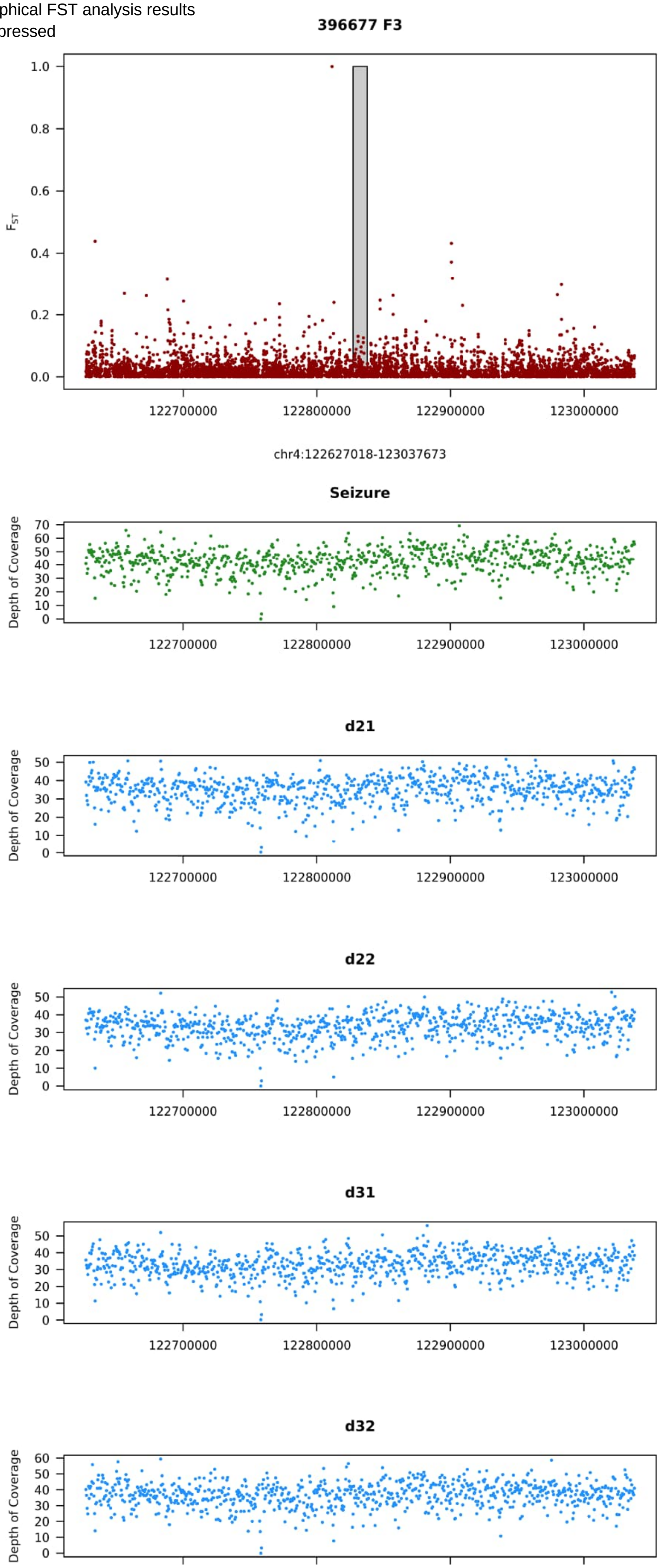

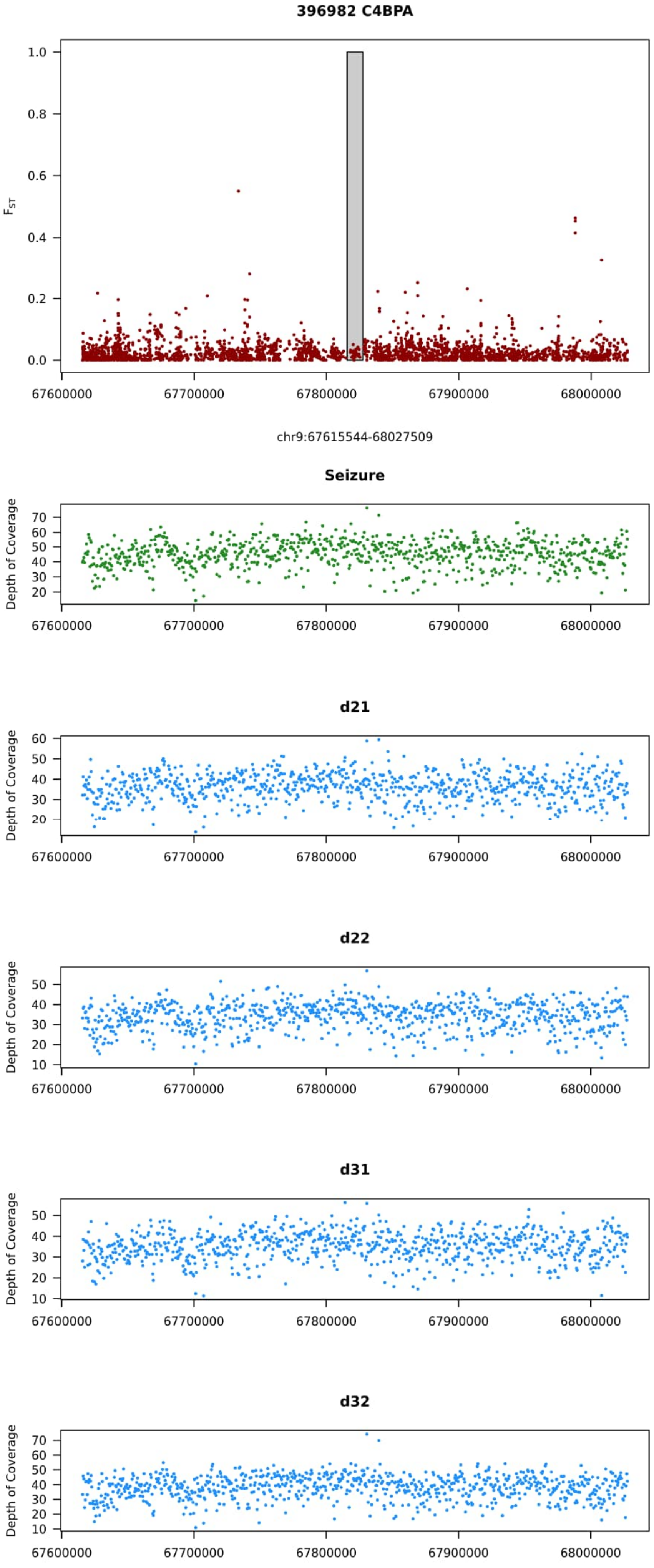

396982 C4BPA
FST
0.0
0.2
0.4
0.6
0.8
1.0
67600000
67700000
67800000
67900000
68000000
chr9:67615544-68027509
Seizure
Depth of Coverage
20 30 40 50 60 70
d21
Depth of Coverage
20 30 40 50 60
d22
Depth of Coverage
10 20 30 40 50
d31
Depth of Coverage
10 20 30 40 50
d32
Depth of Coverage
10 20 30 40 50 60 70

397537 LPL

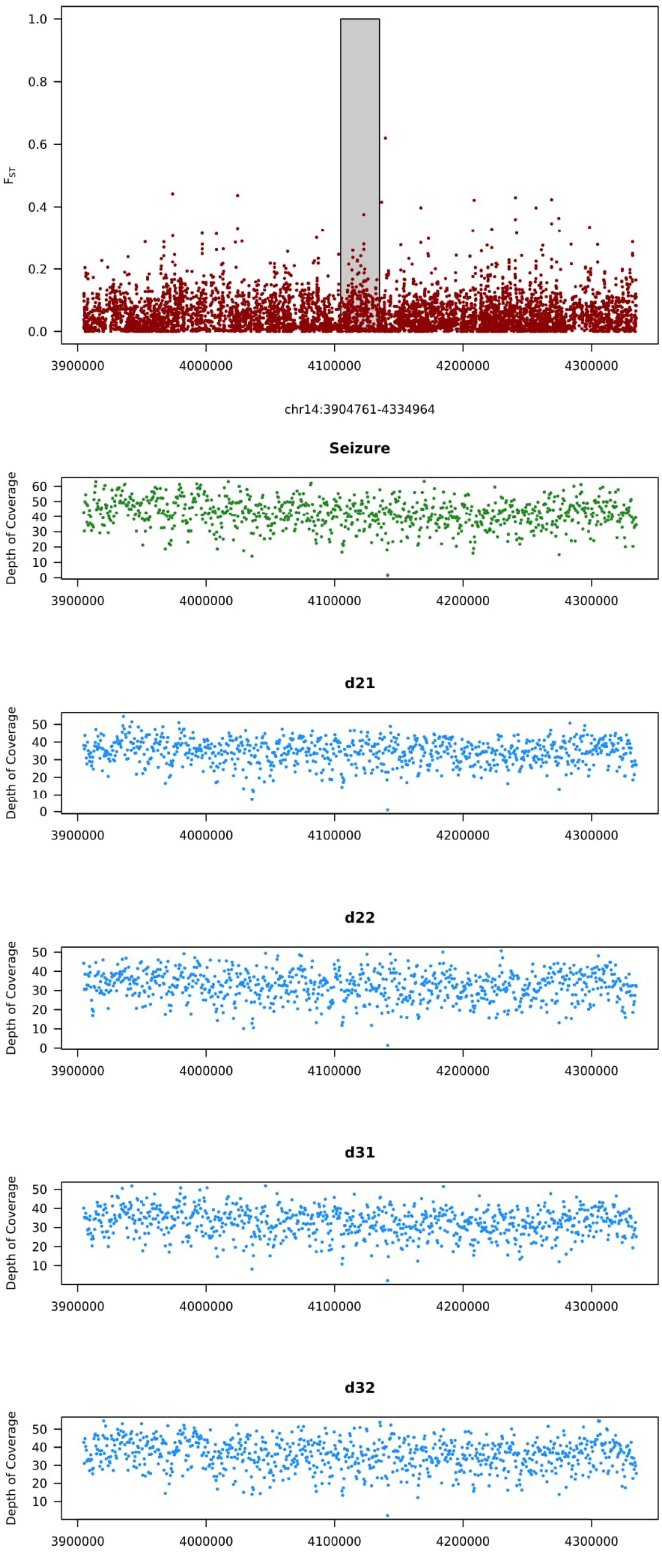

F_ST
chr14:3904761-4334964
Seizure
d21
d22
d31
d32
Depth of Coverage
3900000
4000000
4100000
4200000
4300000

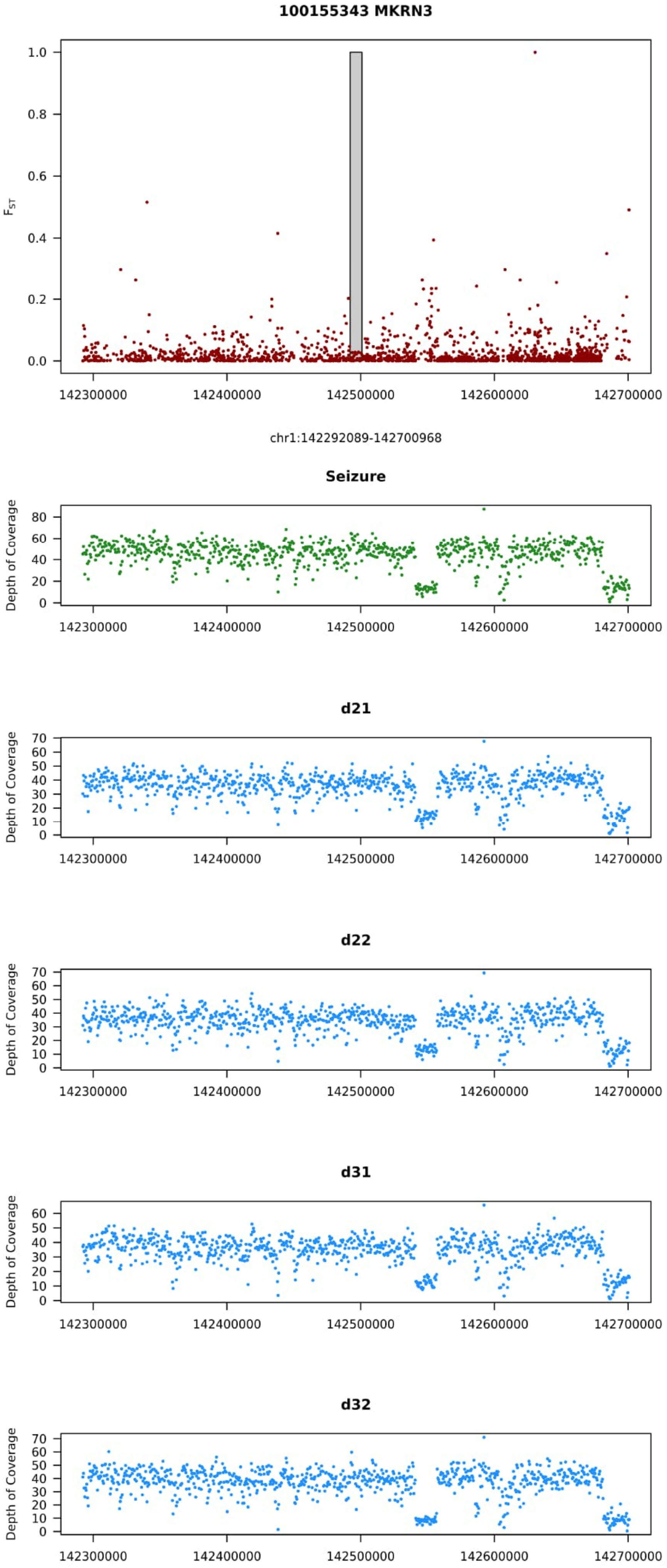
100155343 MKRN3
F_ST
chr1:142292089-142700968
Seizure
Depth of Coverage
d21
Depth of Coverage
d22
Depth of Coverage
d31
Depth of Coverage
d32
Depth of Coverage
142300000
142400000
142500000
142600000
142700000

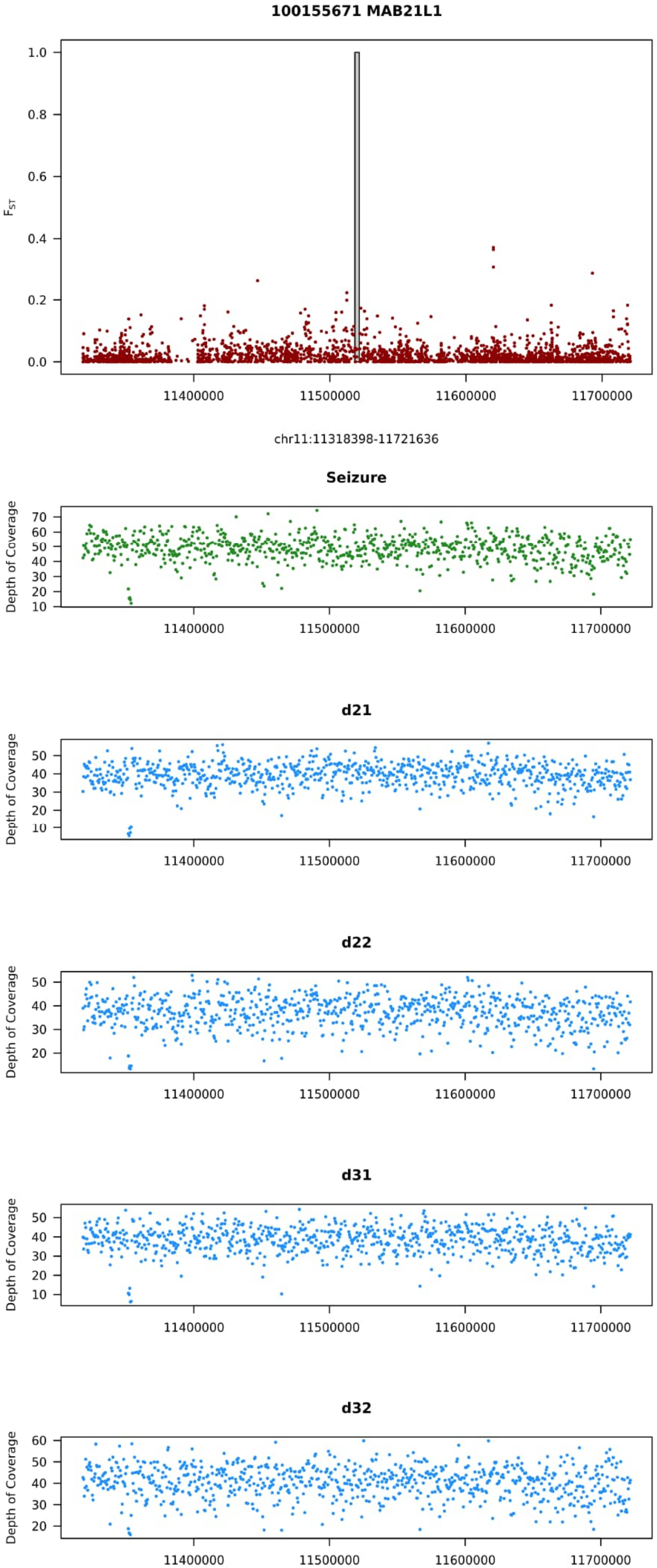
100155671 MAB21L1
1.0
0.8
0.6
0.4
0.2
0.0
F_{ST}
11400000
11500000
11600000
11700000
chr11:11318398-11721636
Seizure
Depth of Coverage
70
60
50
40
30
20
10
d21
Depth of Coverage
d22
Depth of Coverage
d31
Depth of Coverage
d32
Depth of Coverage

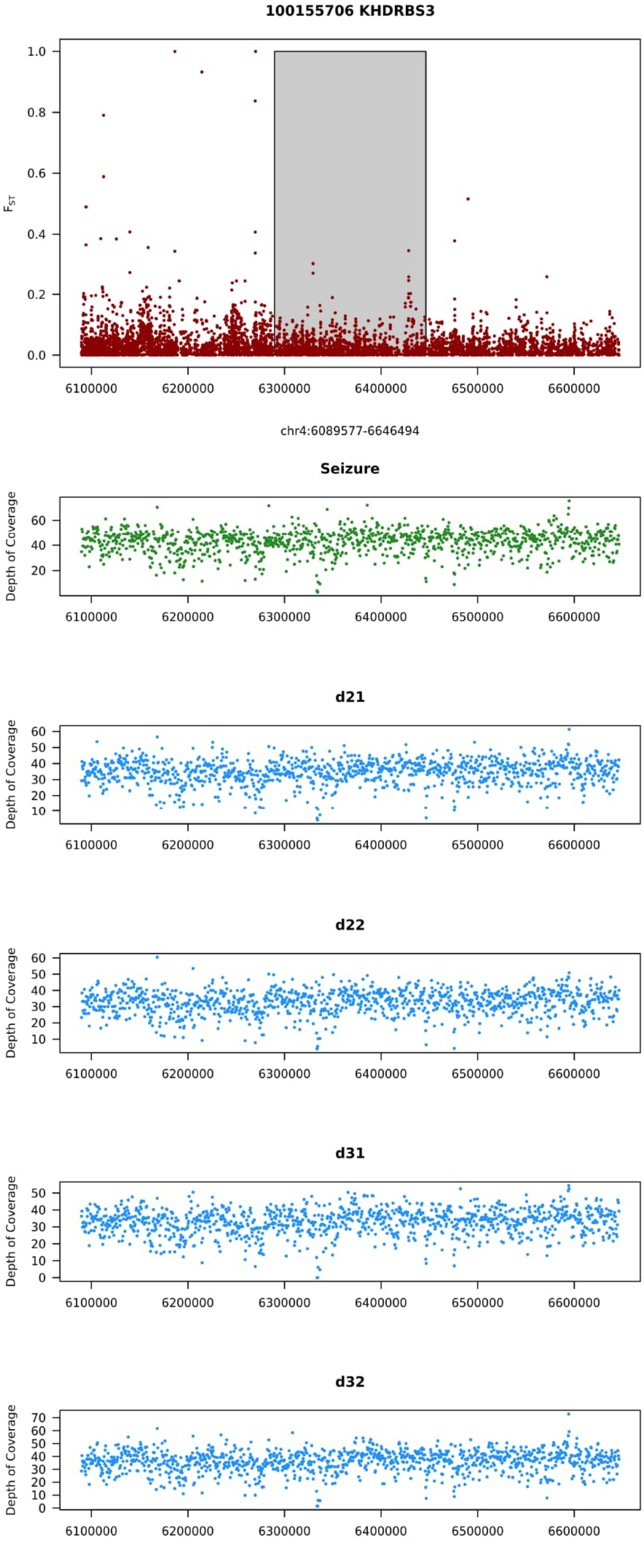
100155706 KHDRBS3
F_{ST}
1.0
0.8
0.6
0.4
0.2
0.0
6100000
6200000
6300000
6400000
6500000
6600000
chr4:6089577-6646494
Seizure
Depth of Coverage
d21
d22
d31
d32

100155963 MMRN2

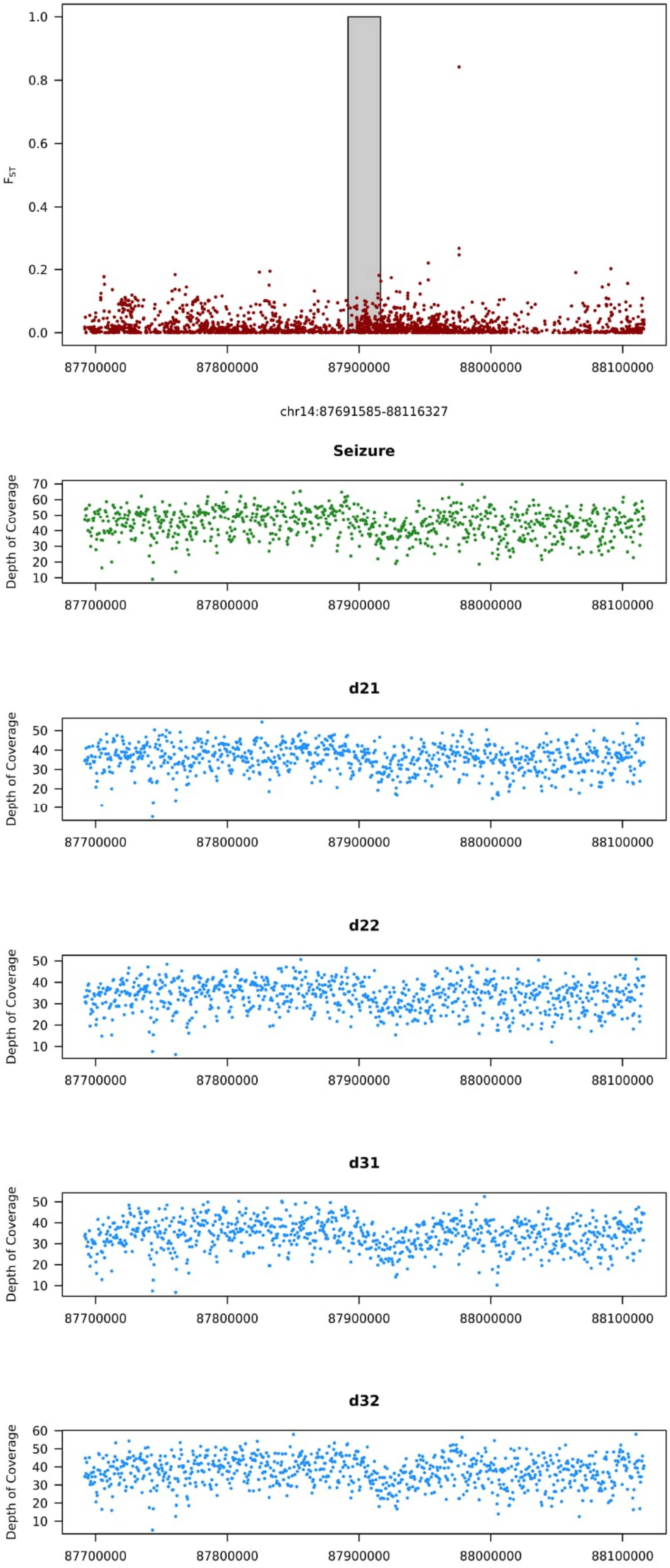

100156493 MN1

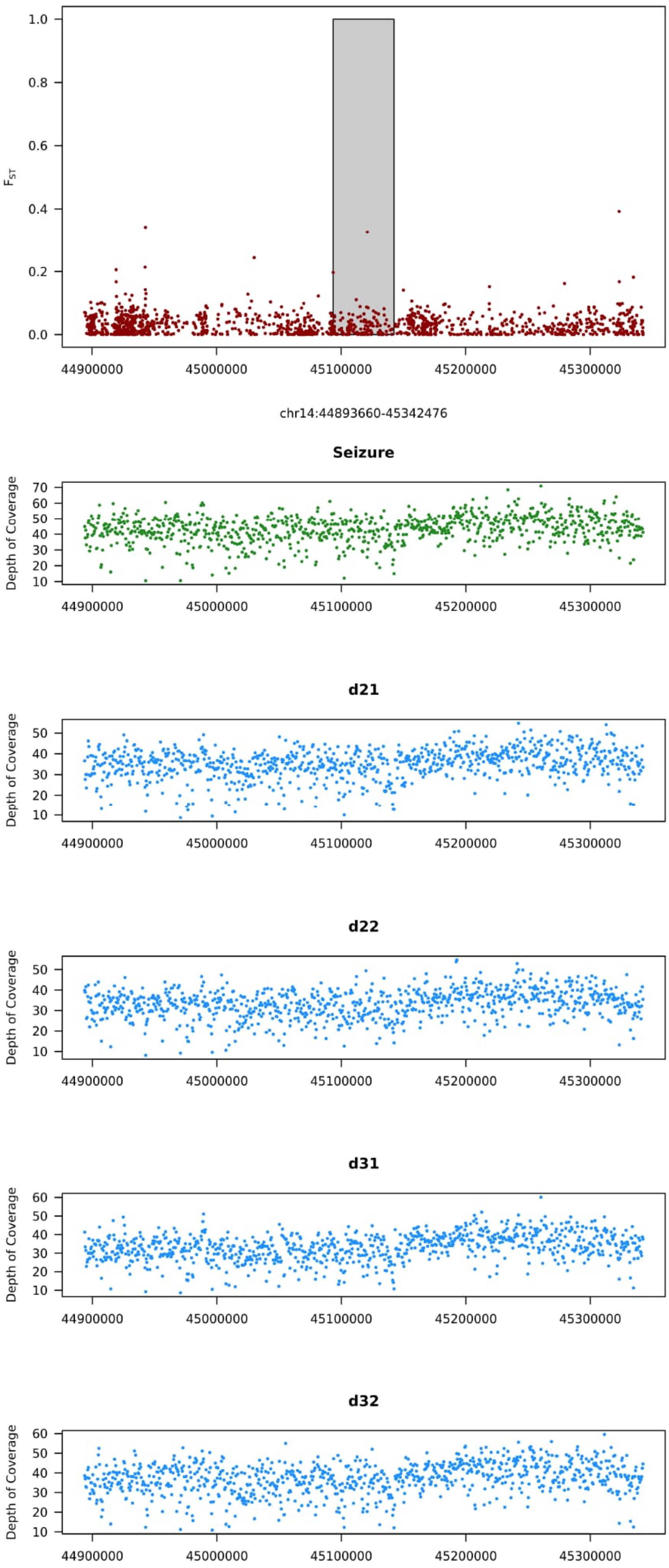

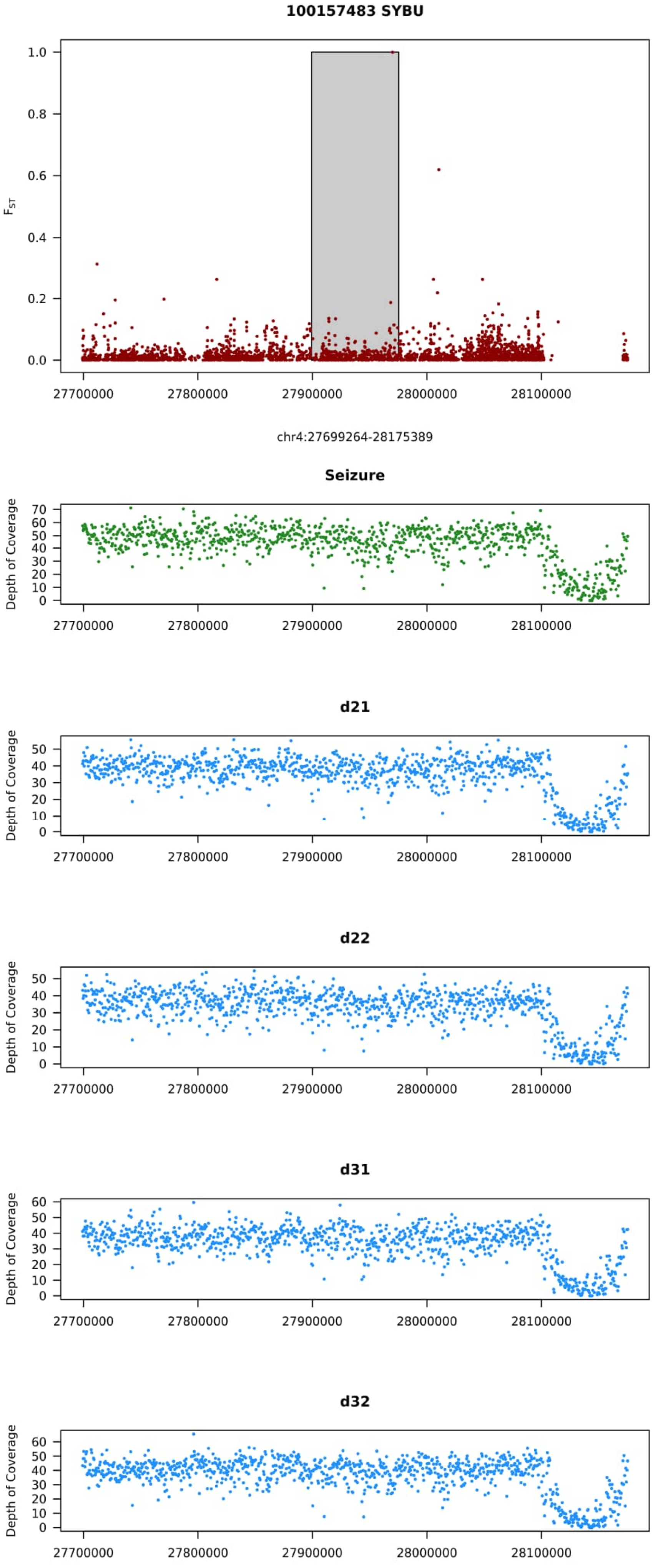
100157483 SYBU
F_{ST}
chr4:27699264-28175389
Seizure
d21
d22
d31
d32
Depth of Coverage
27700000
27800000
27900000
28000000
28100000

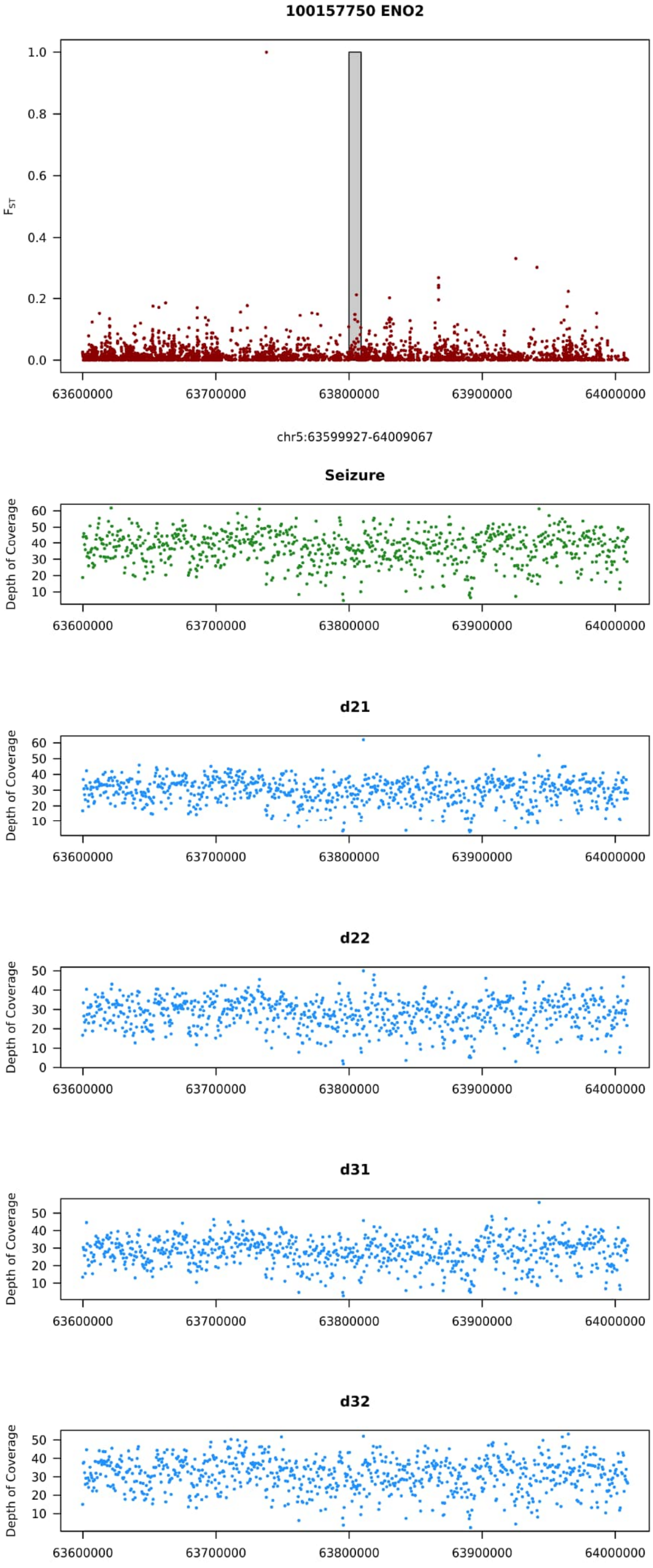

100157750 ENO2
FST
1.0
0.8
0.6
0.4
0.2
0.0
63600000
63700000
63800000
63900000
64000000
chr5:63599927-64009067
Seizure
Depth of Coverage
d21
Depth of Coverage
d22
Depth of Coverage
d31
Depth of Coverage
d32
Depth of Coverage

100157961 FERMT1

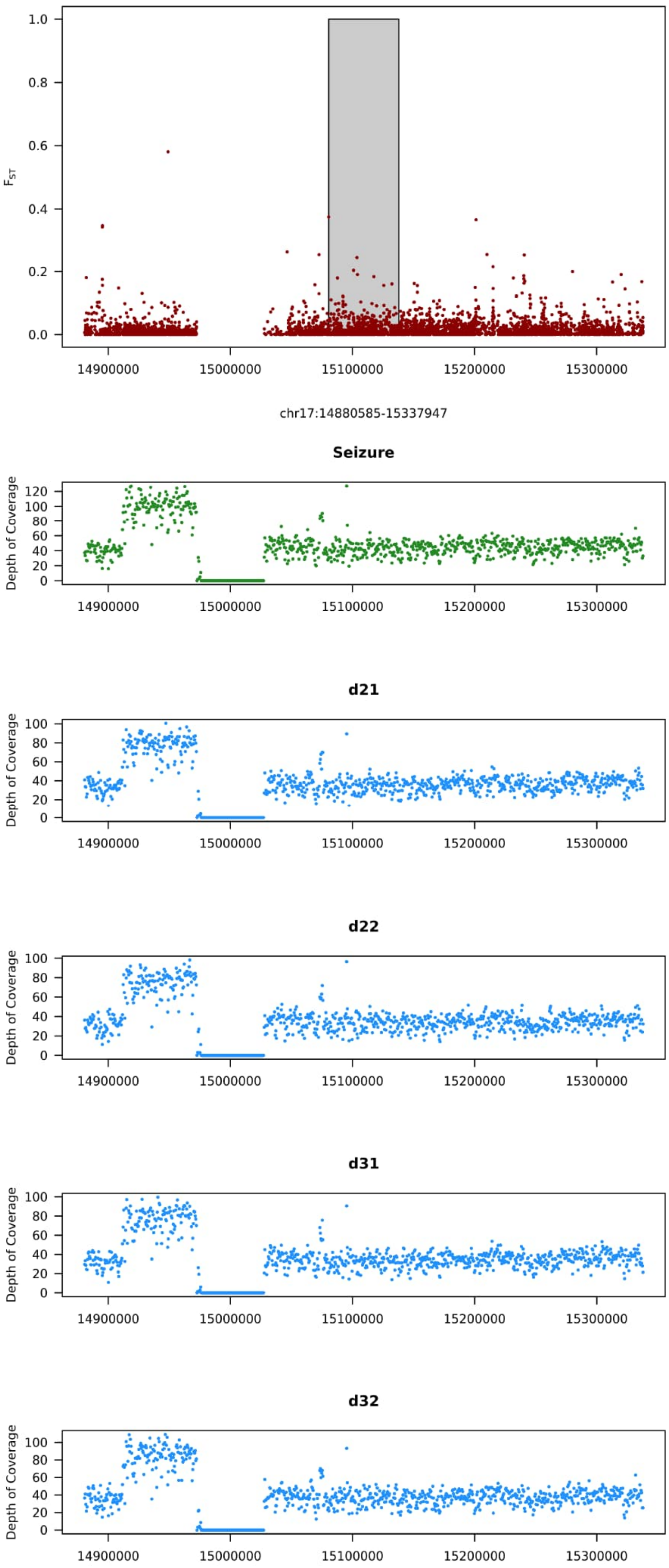

100302016 CLDN11

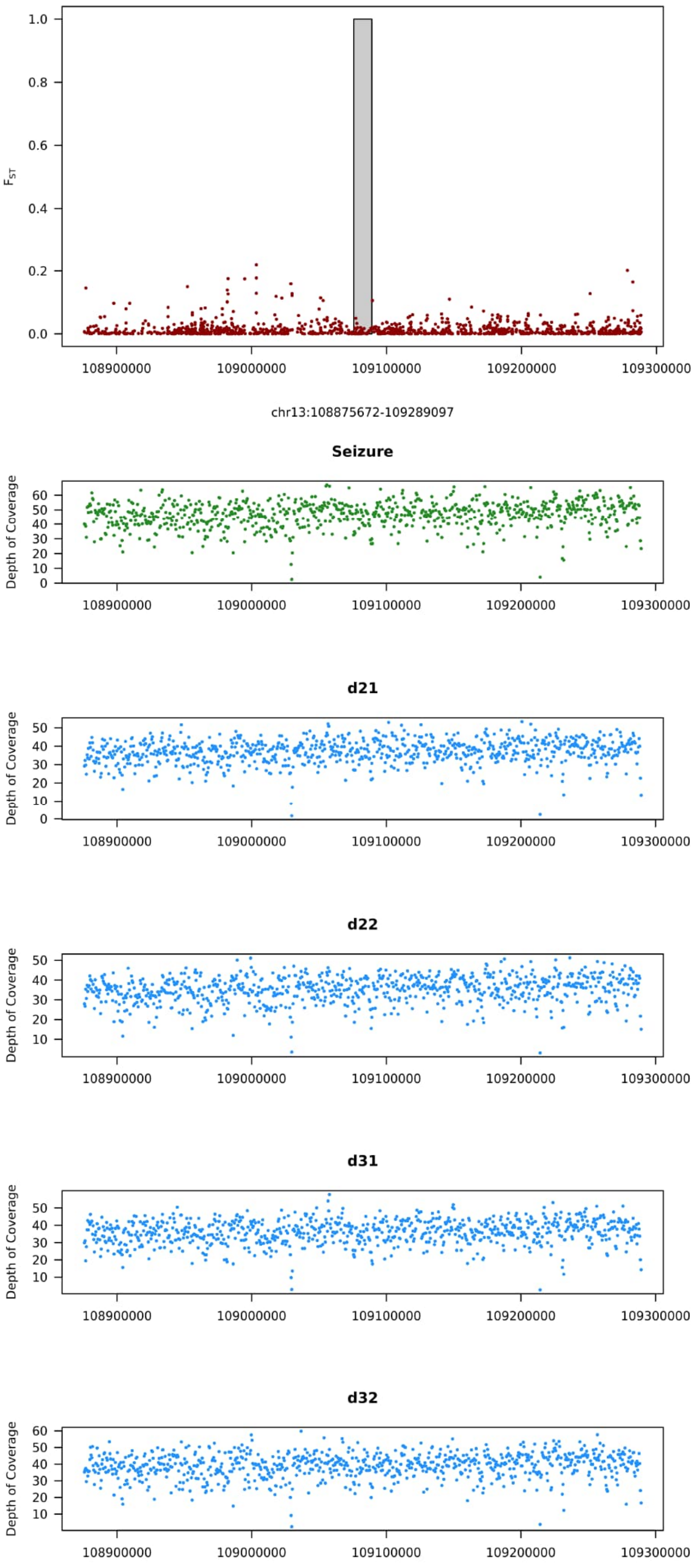

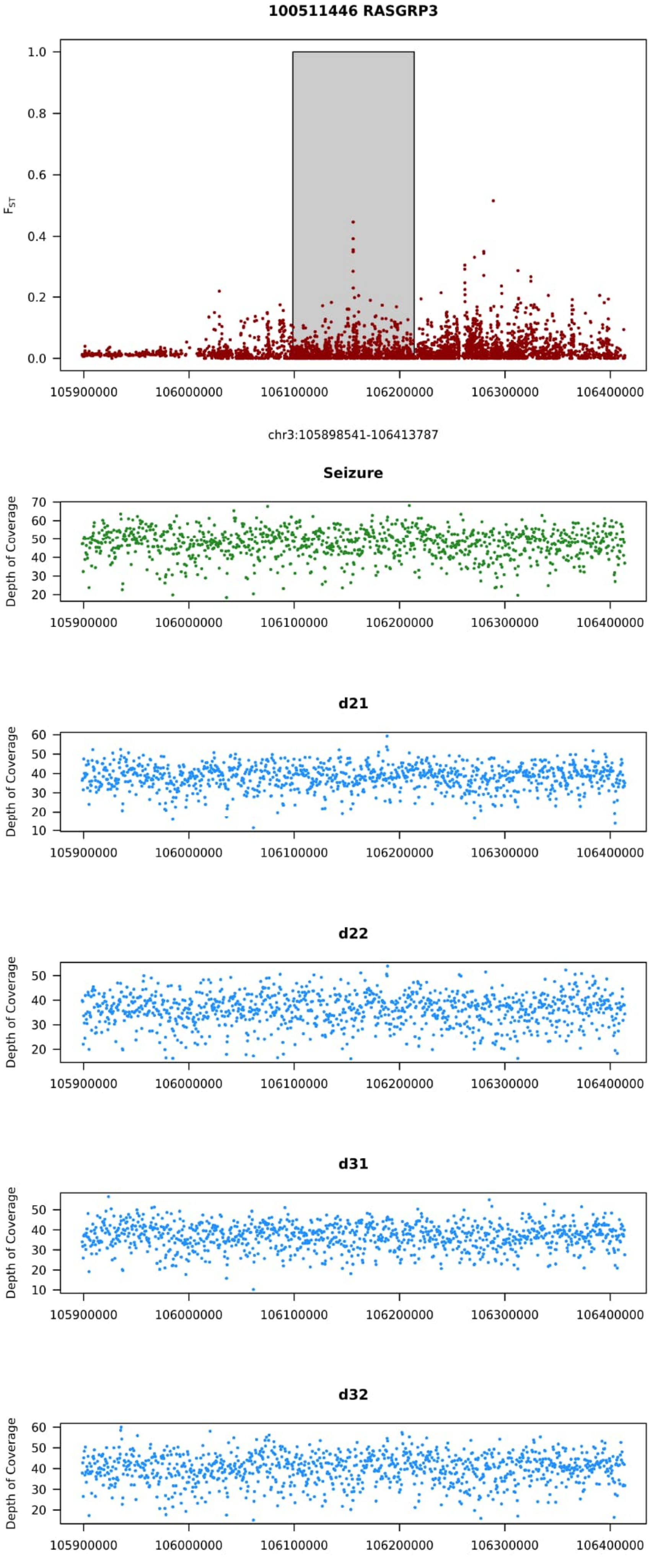
100511446 RASGRP3
F_ST
chr3:105898541-106413787
Seizure
d21
d22
d31
d32
Depth of Coverage
105900000
106000000
106100000
106200000
106300000
106400000

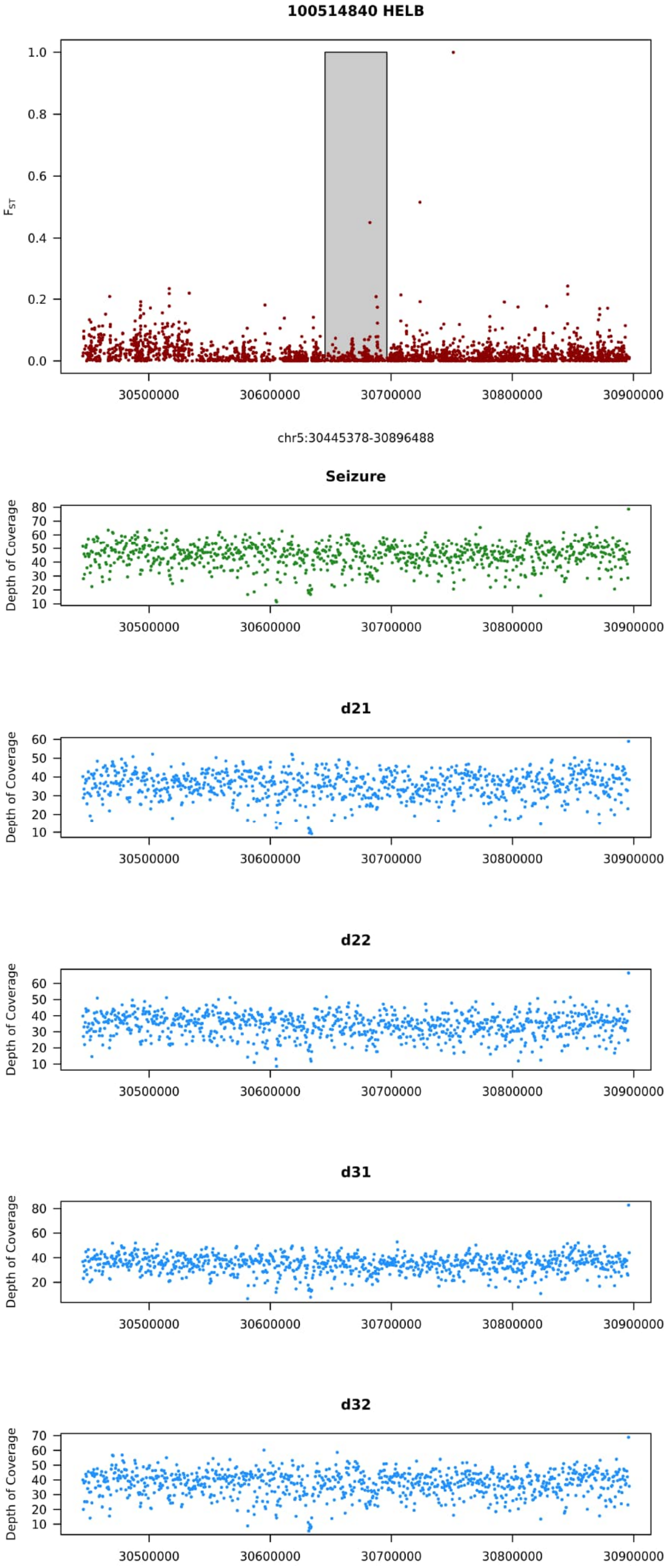
100514840 HELB
F_ST
1.0
0.8
0.6
0.4
0.2
0.0
30500000
30600000
30700000
30800000
30900000
chr5:30445378-30896488
Seizure
Depth of Coverage
d21
Depth of Coverage
d22
Depth of Coverage
d31
Depth of Coverage
d32
Depth of Coverage

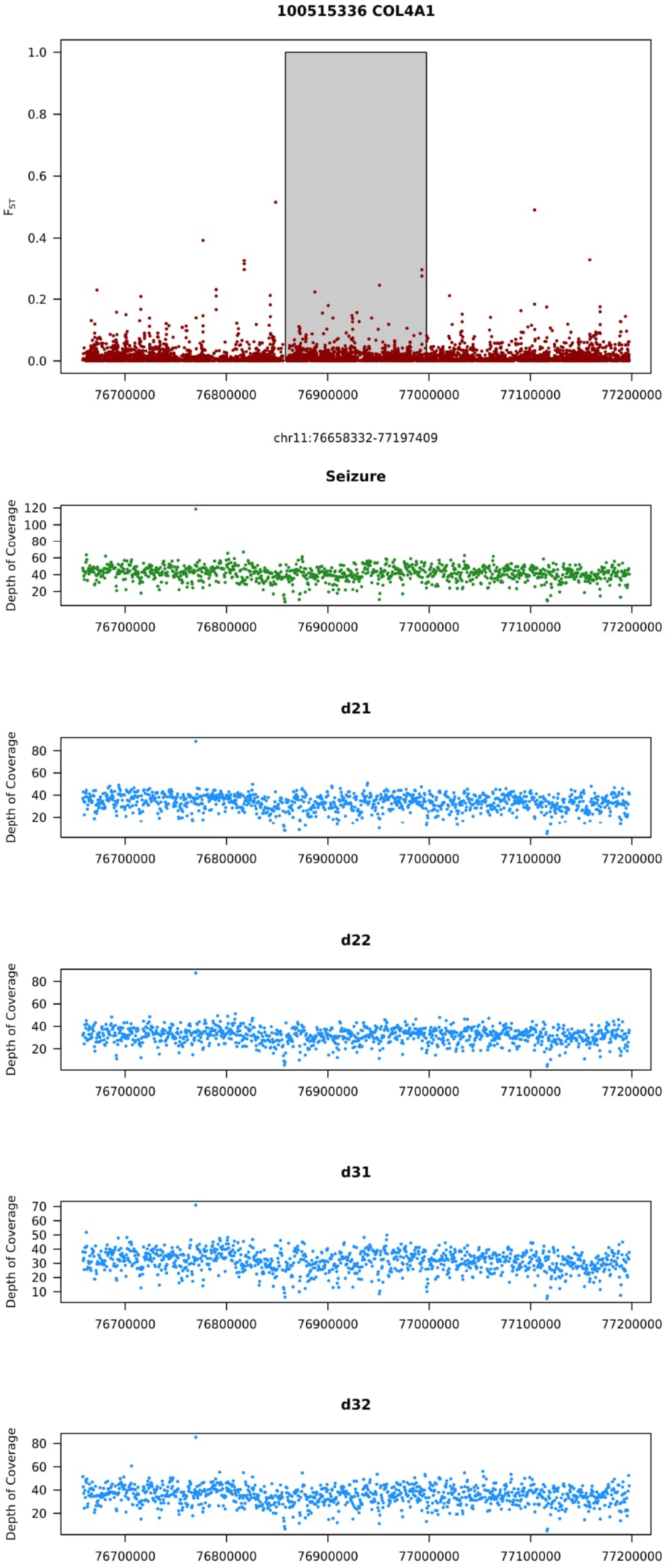

100515336 COL4A1
F_ST
chr11:76658332-77197409
Seizure
d21
d22
d31
d32
Depth of Coverage
76700000
76800000
76900000
77000000
77100000
77200000

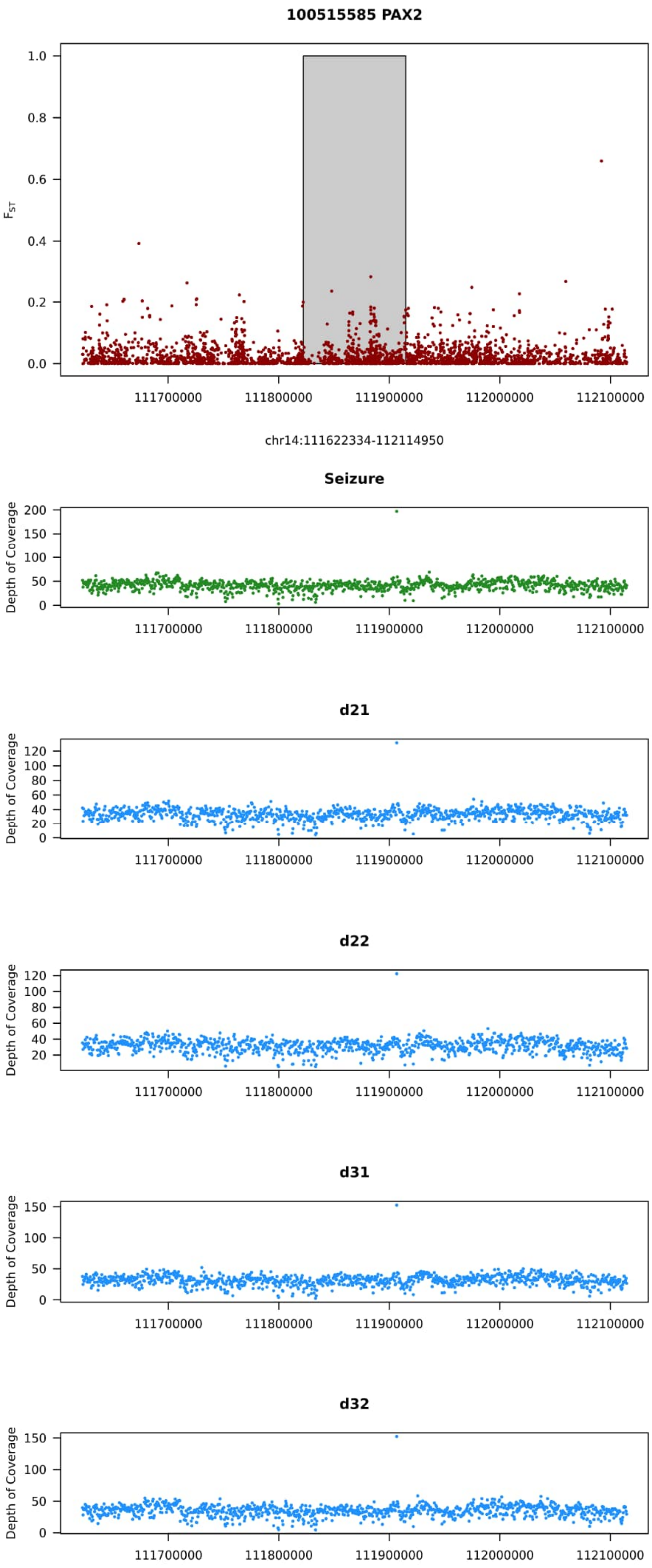
100515585 PAX2
FST
1.0
0.8
0.6
0.4
0.2
0.0
111700000
111800000
111900000
112000000
112100000
chr14:111622334-112114950
Seizure
Depth of Coverage
200
150
100
50
0
d21
120
100
80
60
40
20
d22
d31
d32

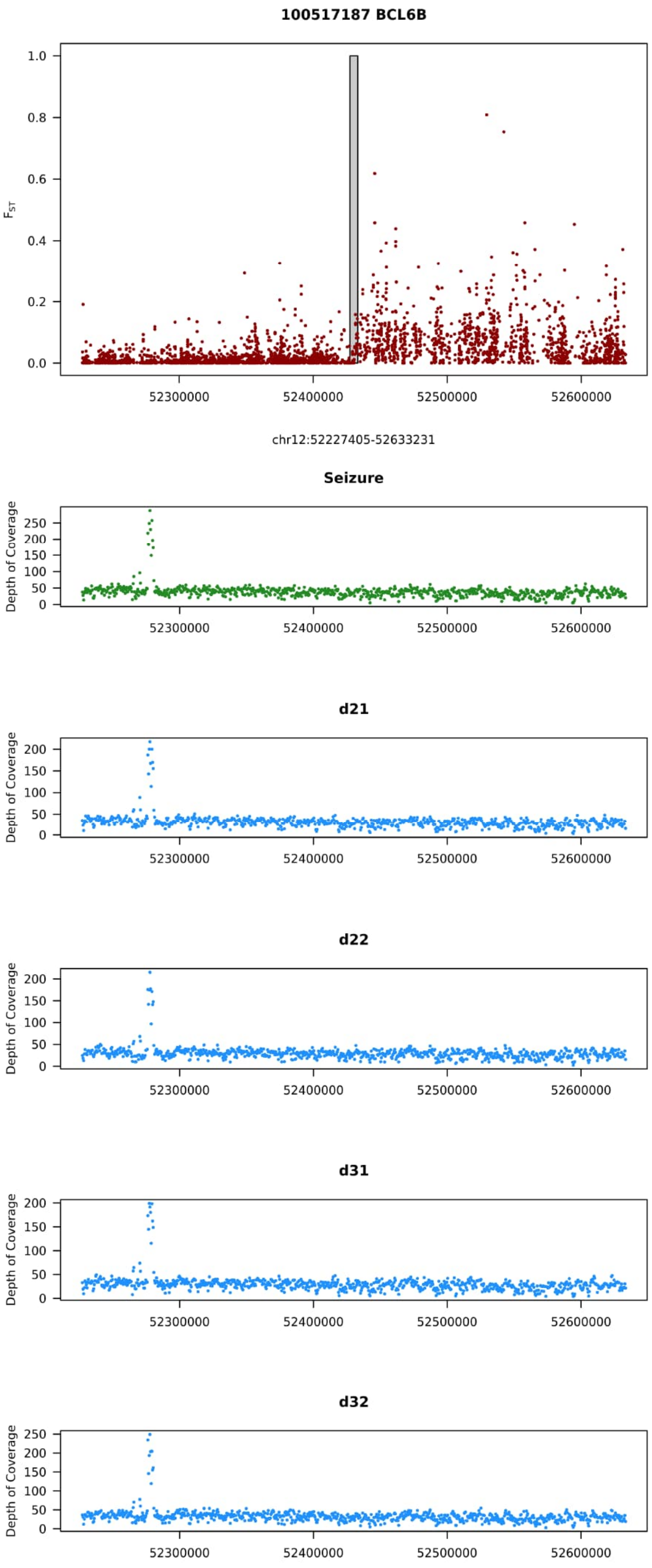
100517187 BCL6B
F_ST
chr12:52227405-52633231
Seizure
d21
d22
d31
d32
Depth of Coverage
52300000
52400000
52500000
52600000

100518432 MYCL

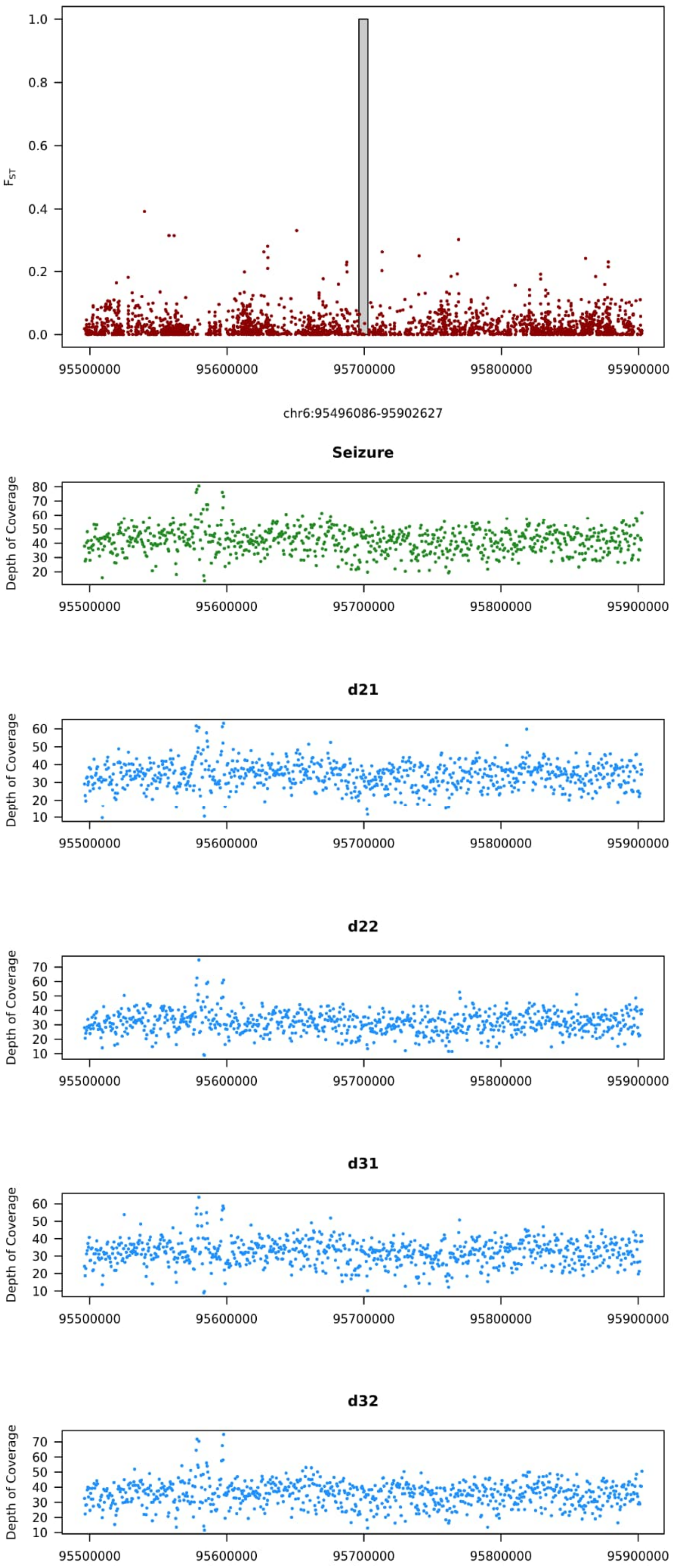

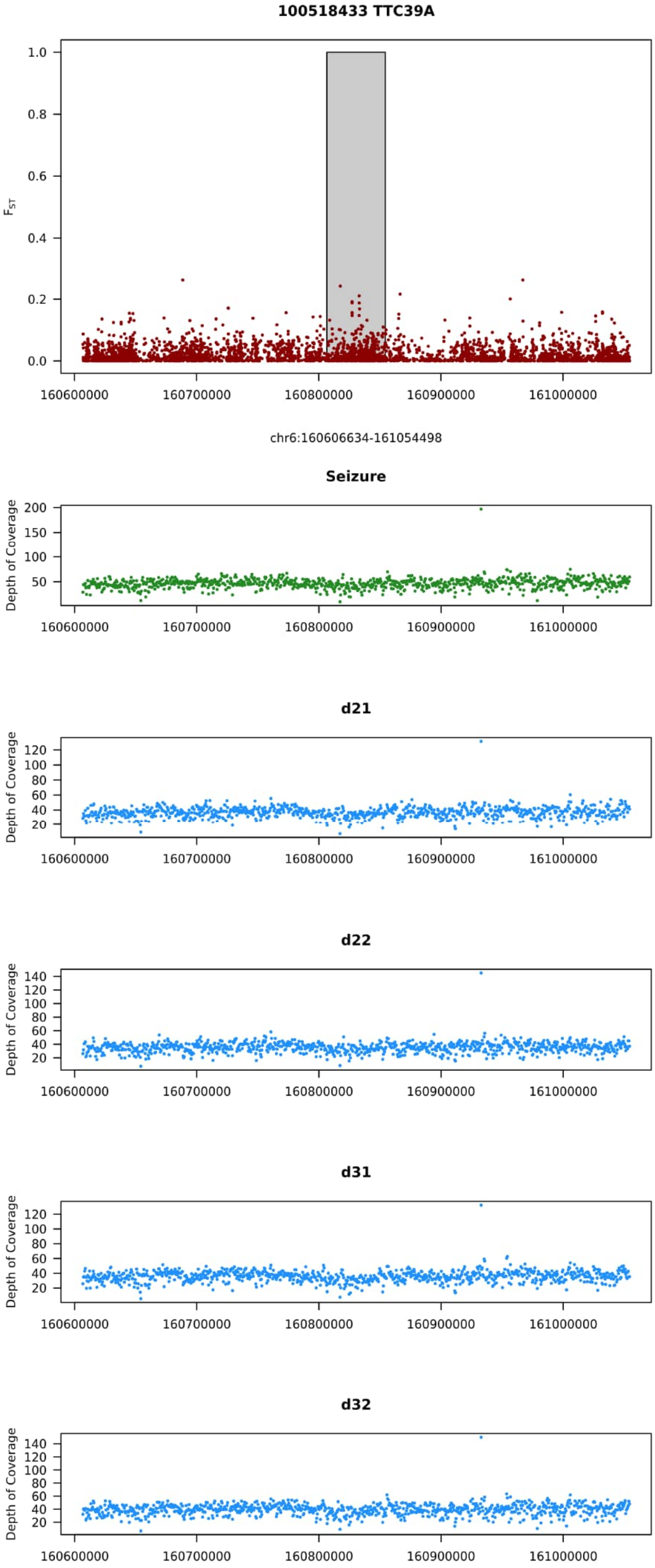

100518433 TTC39A
FST
1.0
0.8
0.6
0.4
0.2
0.0
160600000
160700000
160800000
160900000
161000000
chr6:160606634-161054498
Seizure
Depth of Coverage
200
150
100
50
d21
120
100
80
60
40
20
d22
140
d31
d32

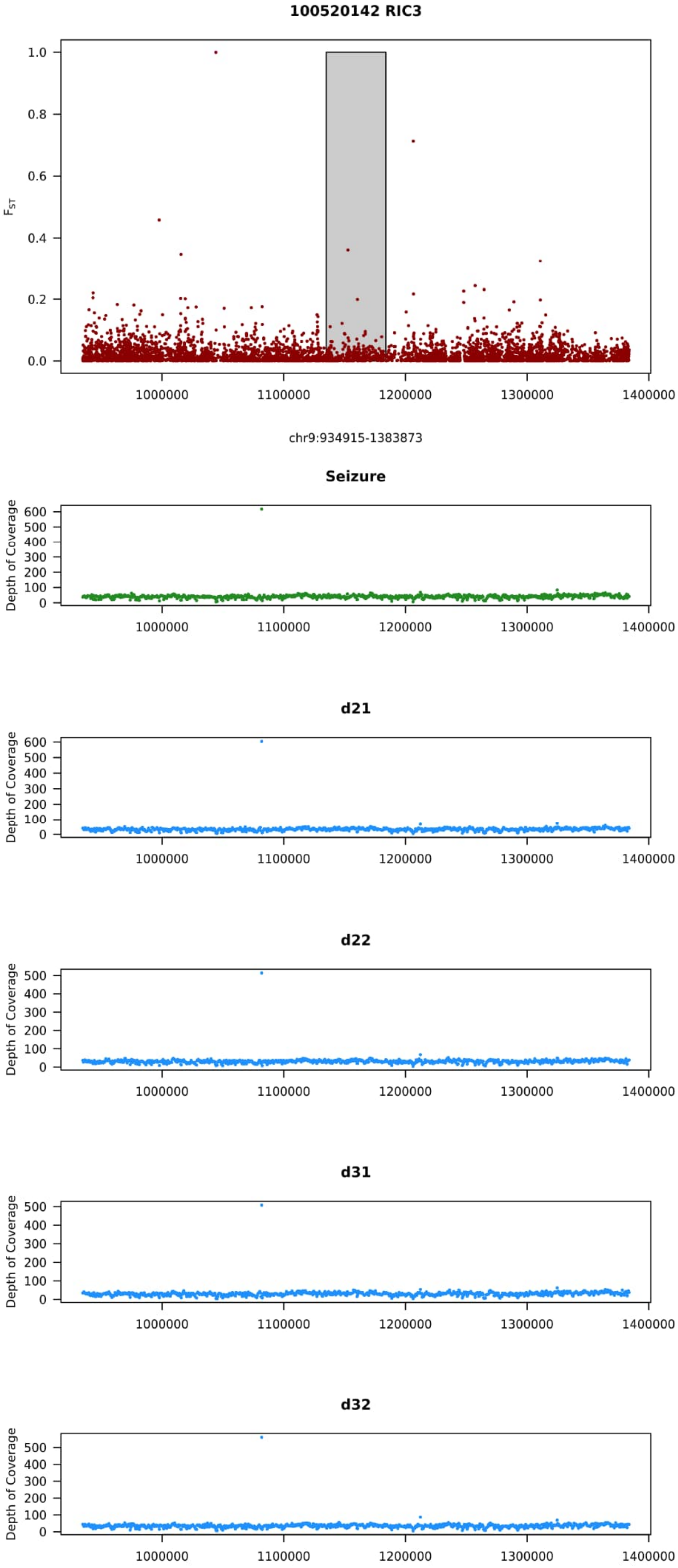
100520142 RIC3
F_ST
chr9:934915-1383873
Seizure
d21
d22
d31
d32
Depth of Coverage
1000000
1100000
1200000
1300000
1400000

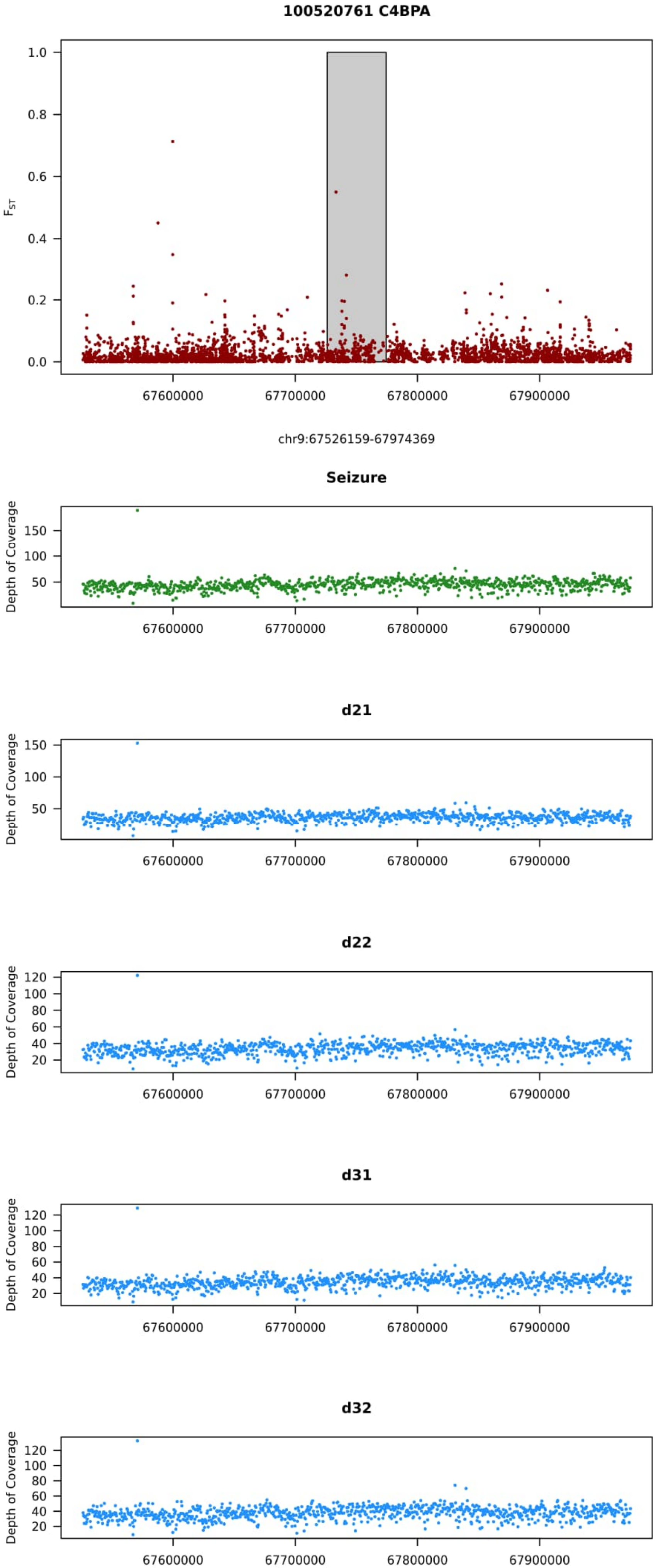
100520761 C4BPA
F_{ST}
1.0
0.8
0.6
0.4
0.2
0.0
67600000
67700000
67800000
67900000
chr9:67526159-67974369
Seizure
Depth of Coverage
150
100
50
d21
d22
120
100
80
60
40
20
d31
d32

100522280 TBX5

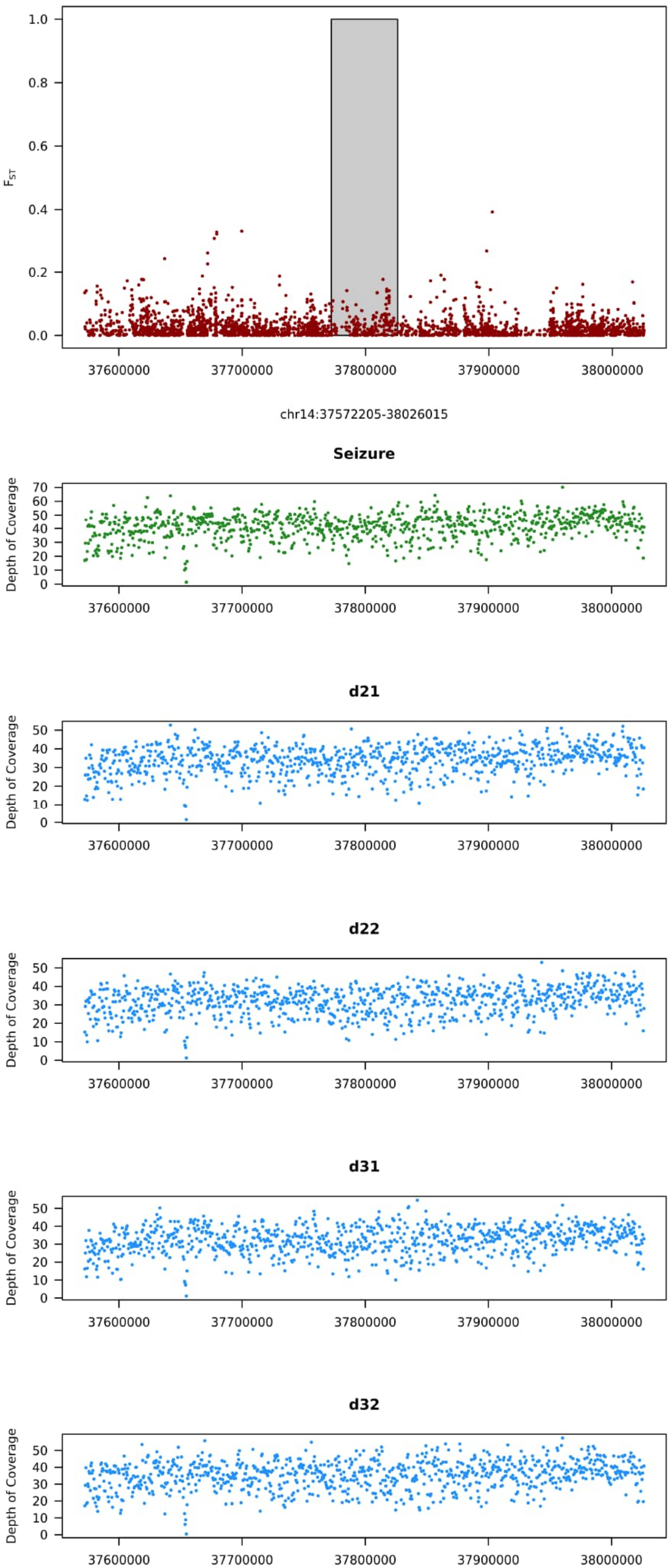

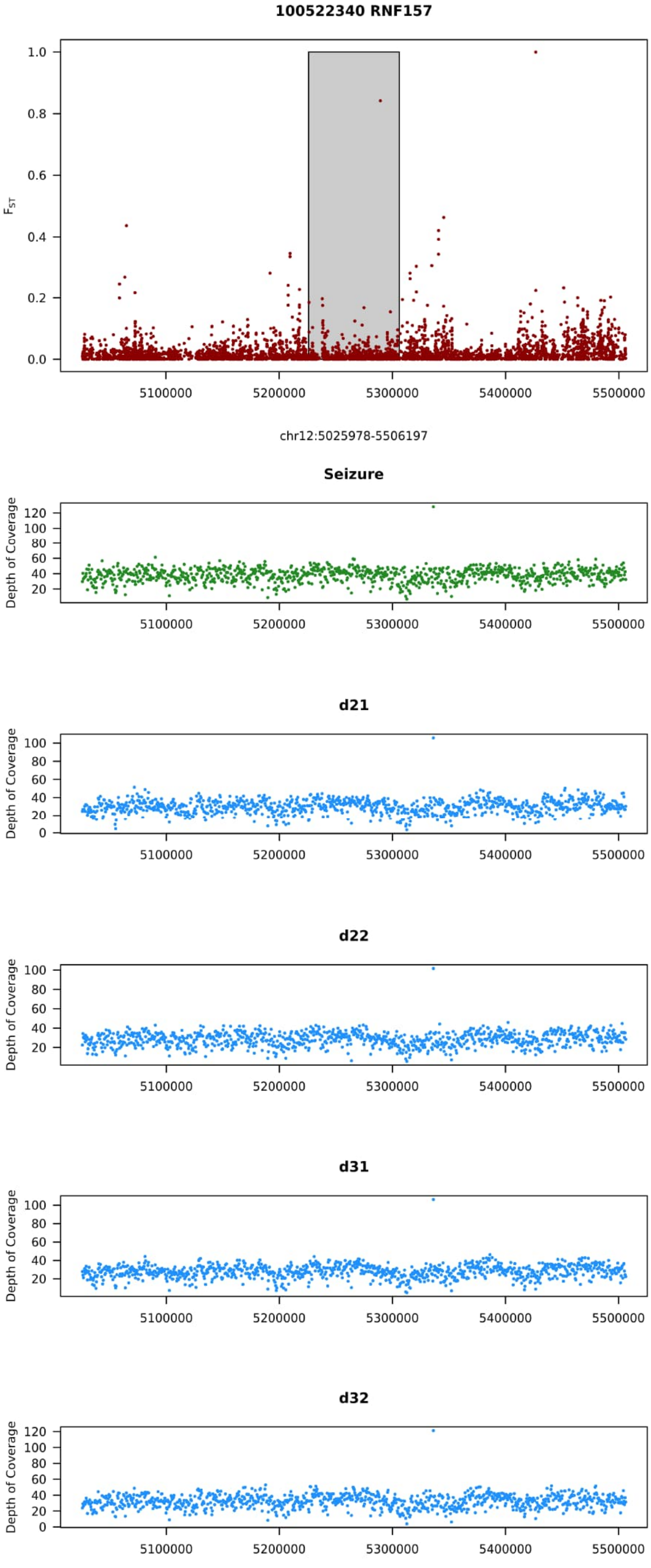
100522340 RNF157
FST
1.0
0.8
0.6
0.4
0.2
0.0
5100000
5200000
5300000
5400000
5500000
chr12:5025978-5506197
Seizure
Depth of Coverage
120
100
80
60
40
20
d21
d22
d31
d32

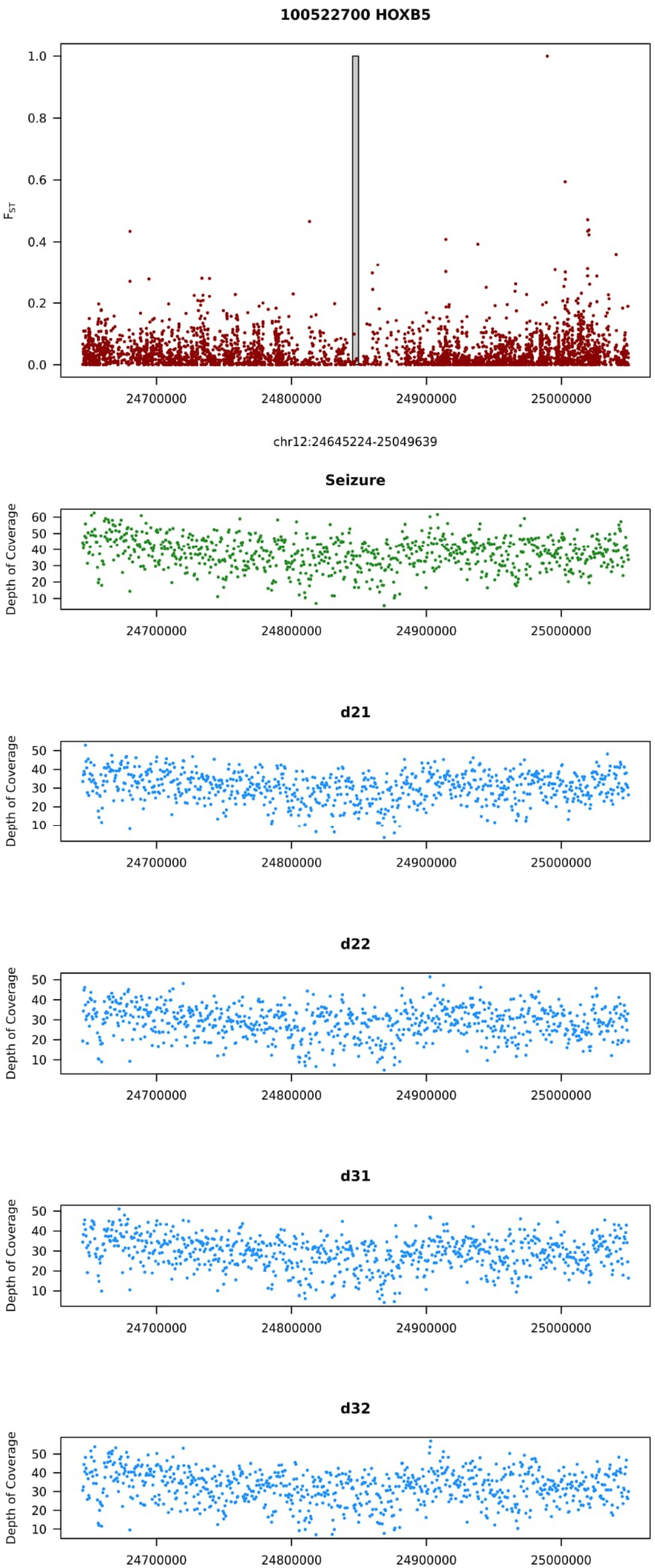
100522700 HOXB5
F_{ST}
chr12:24645224-25049639
Seizure
d21
d22
d31
d32
Depth of Coverage
24700000
24800000
24900000
25000000

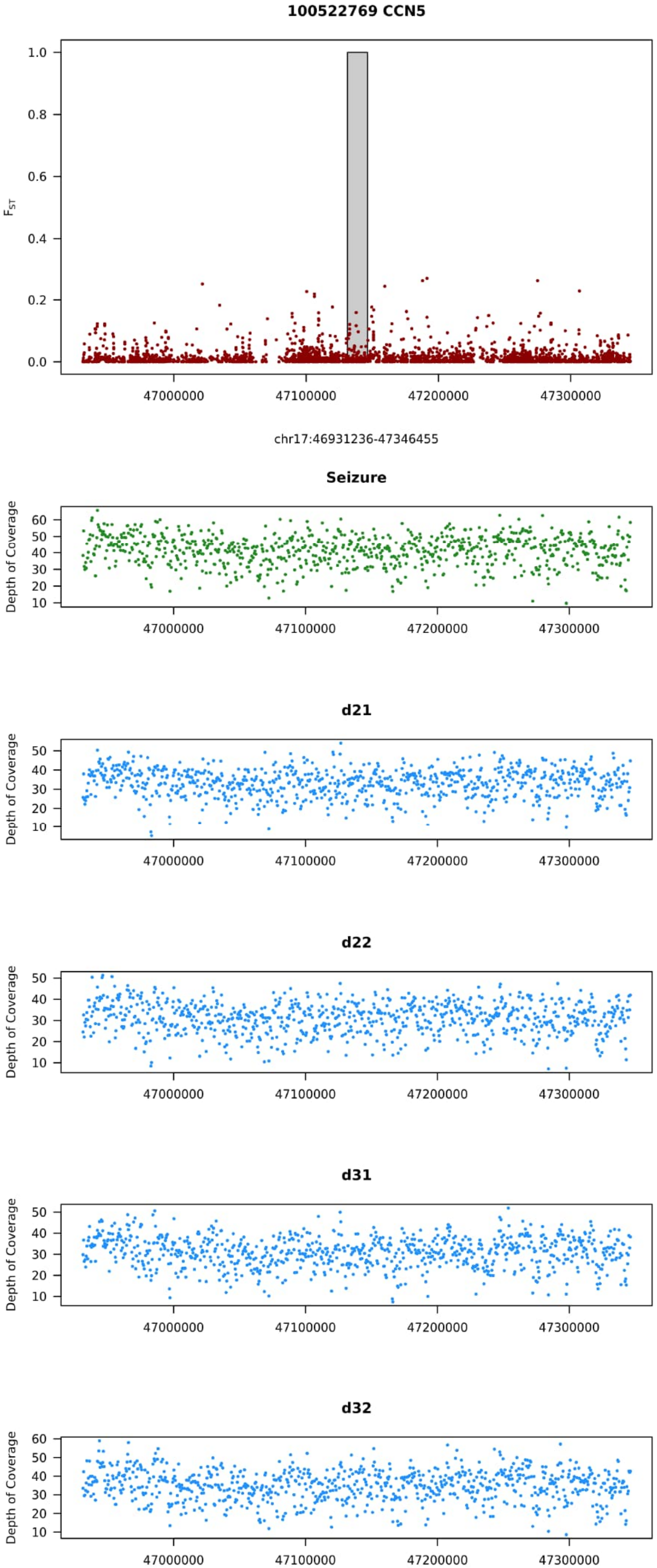
100522769 CCN5
F_ST
1.0
0.8
0.6
0.4
0.2
0.0
47000000
47100000
47200000
47300000
chr17:46931236-47346455
Seizure
Depth of Coverage
d21
d22
d31
d32

100524798 DNAH6

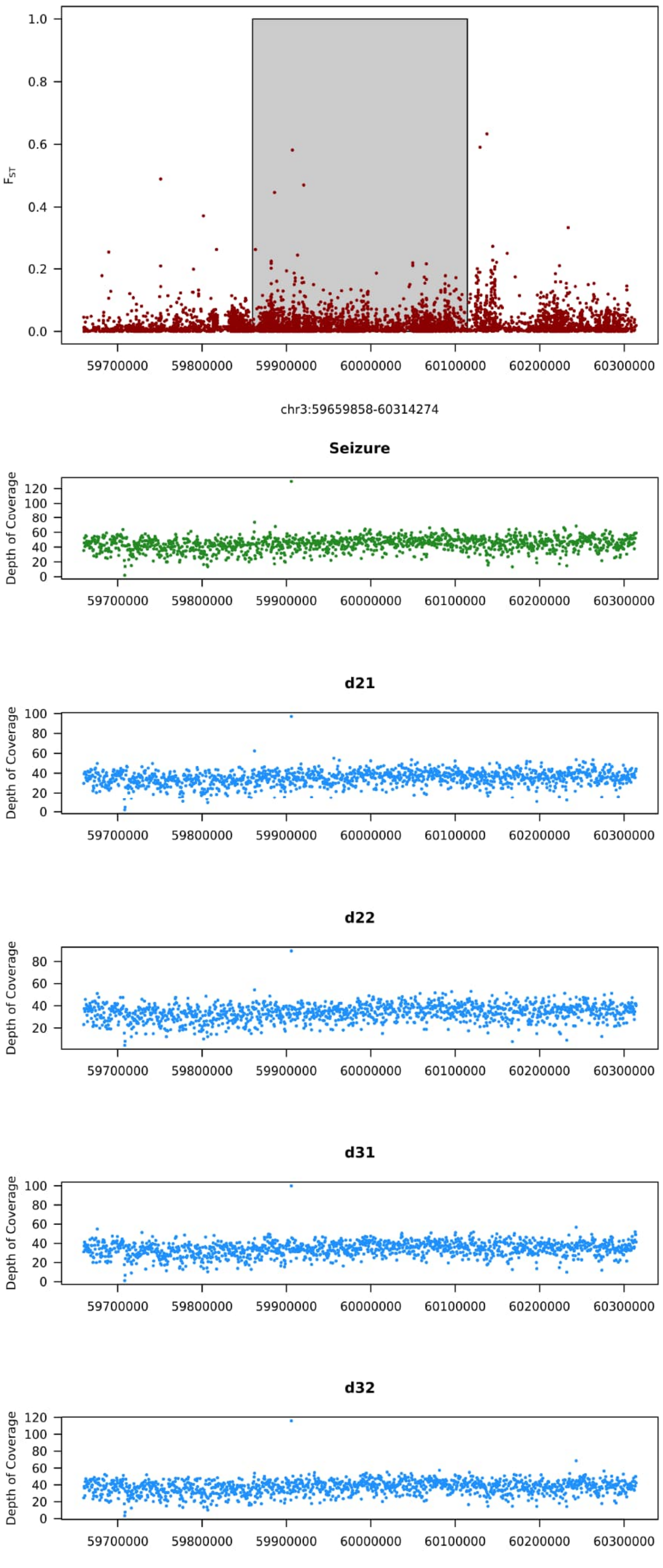

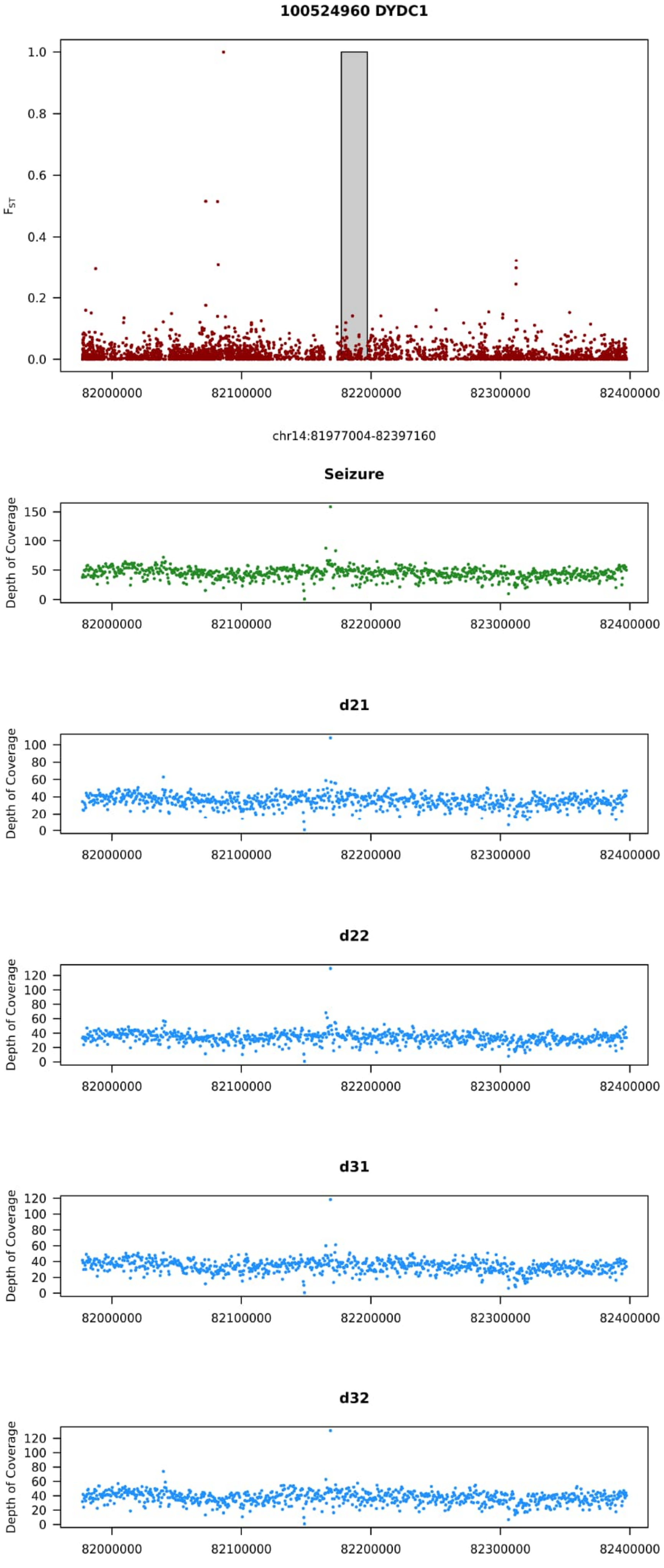
100524960 DYDC1
F_ST
1.0
0.8
0.6
0.4
0.2
0.0
82000000
82100000
82200000
82300000
82400000
chr14:81977004-82397160
Seizure
Depth of Coverage
d21
d22
d31
d32

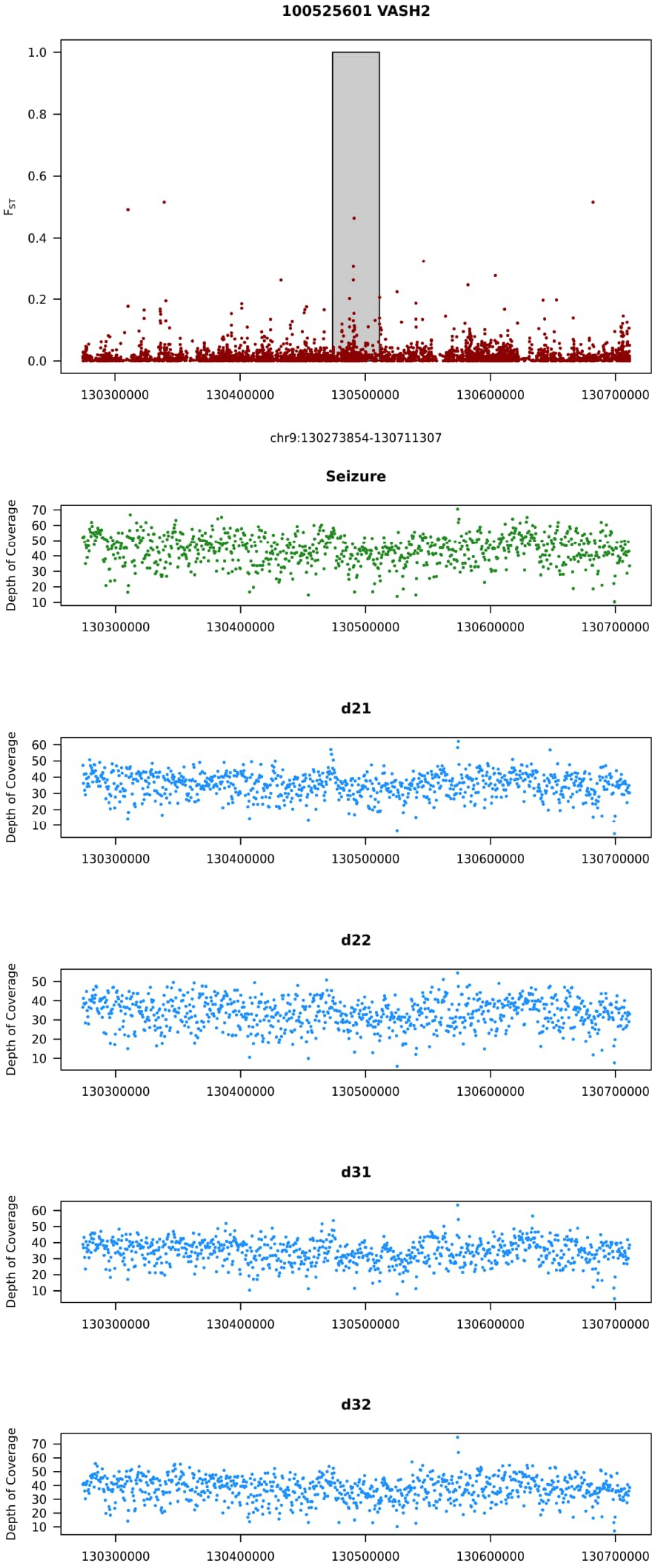

100525601 VASH2
F_ST
1.0
0.8
0.6
0.4
0.2
0.0
130300000
130400000
130500000
130600000
130700000
chr9:130273854-130711307
Seizure
d21
d22
d31
d32
Depth of Coverage

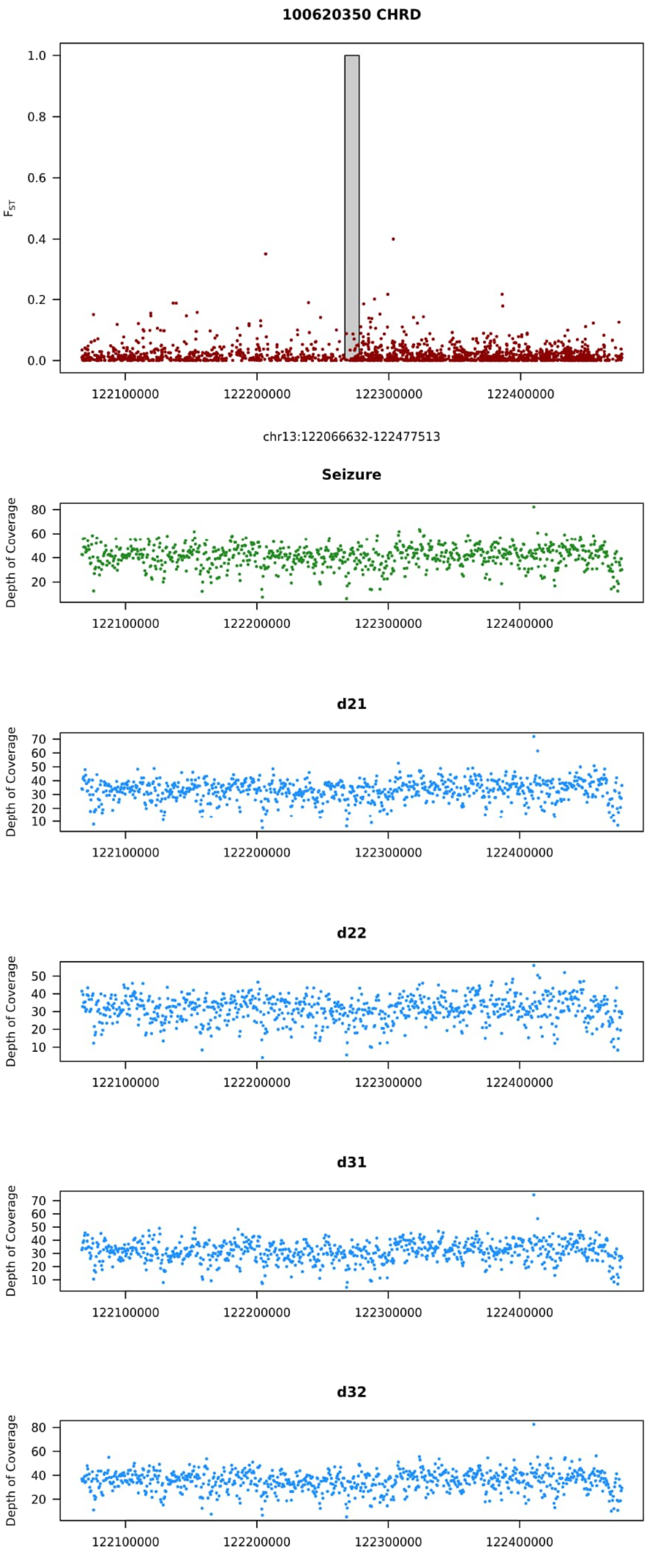

100620350 CHRD
F$_{ST}$
1.0
0.8
0.6
0.4
0.2
0.0
122100000
122200000
122300000
122400000
chr13:122066632-122477513
Seizure
Depth of Coverage
d21
d22
d31
d32

100623340 GPRC5B

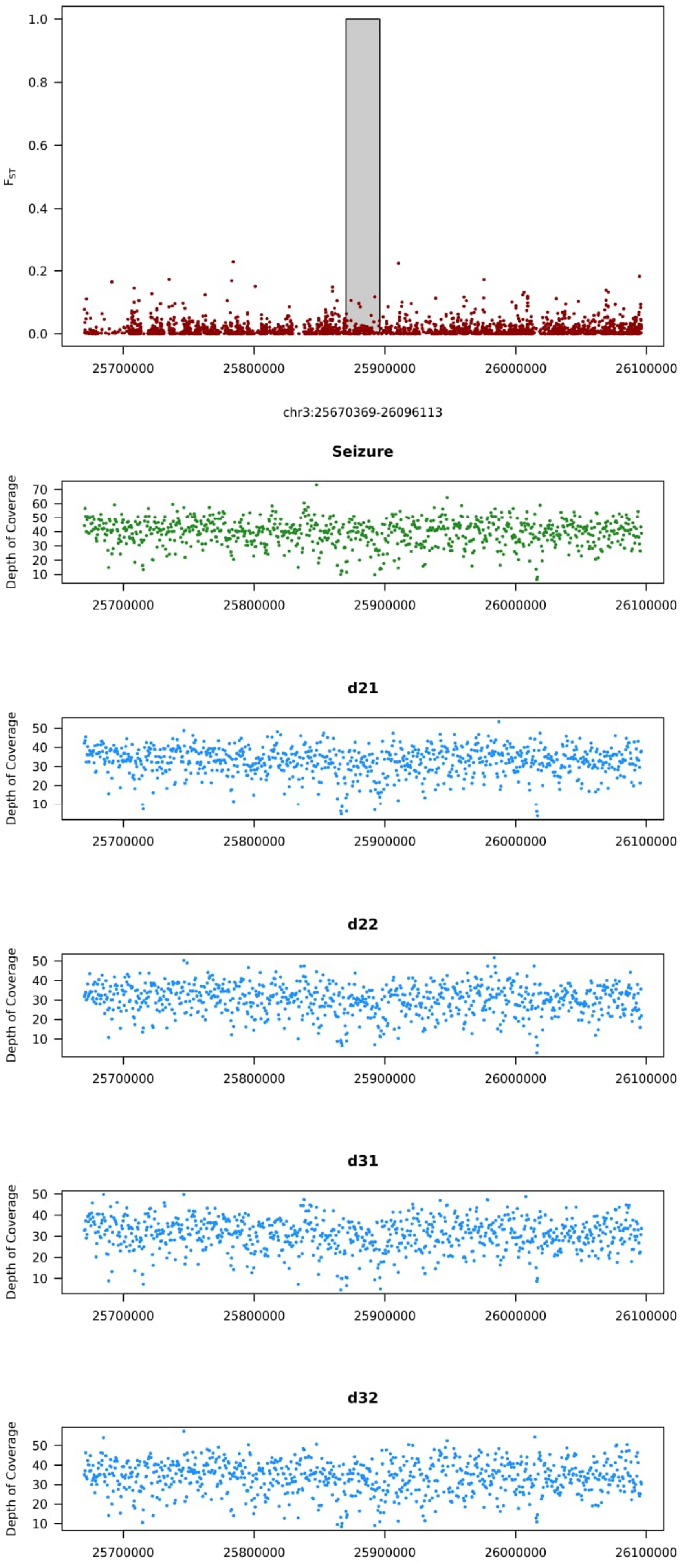

100623437 DSC3

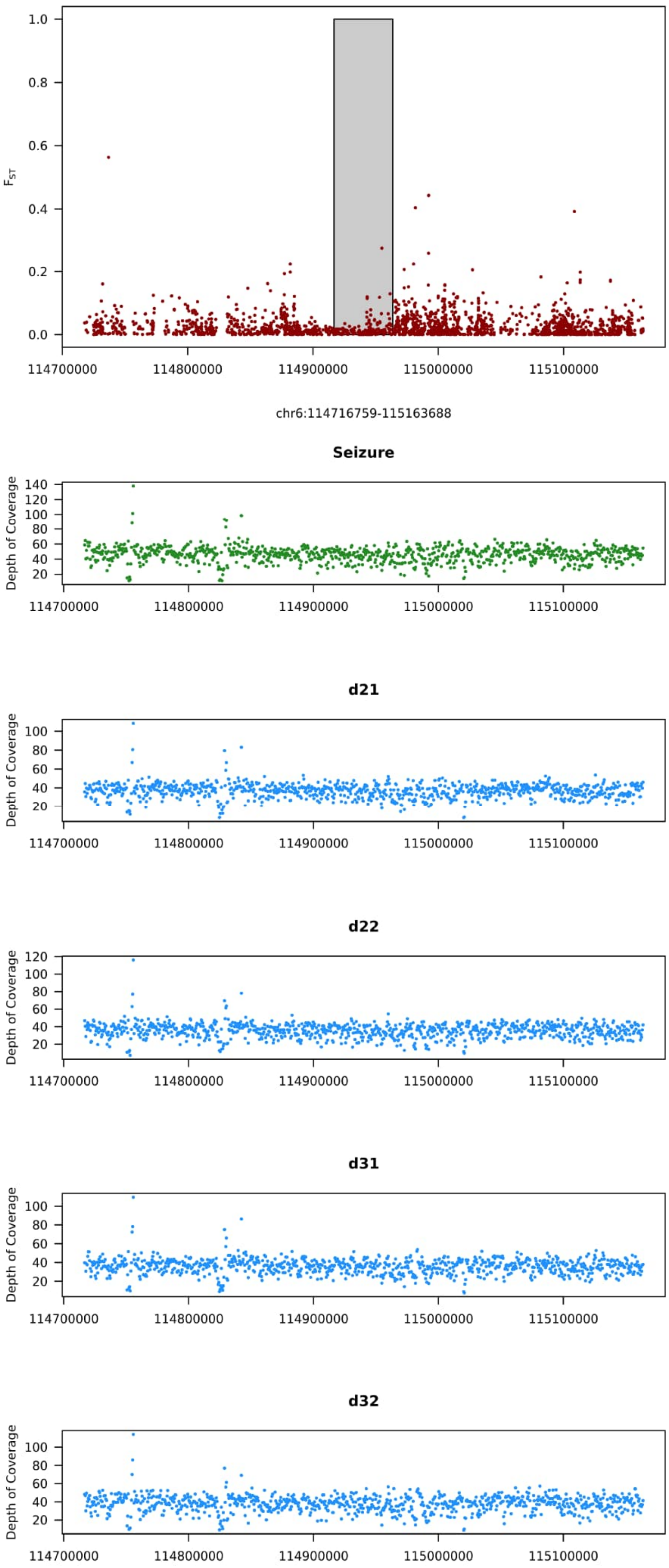

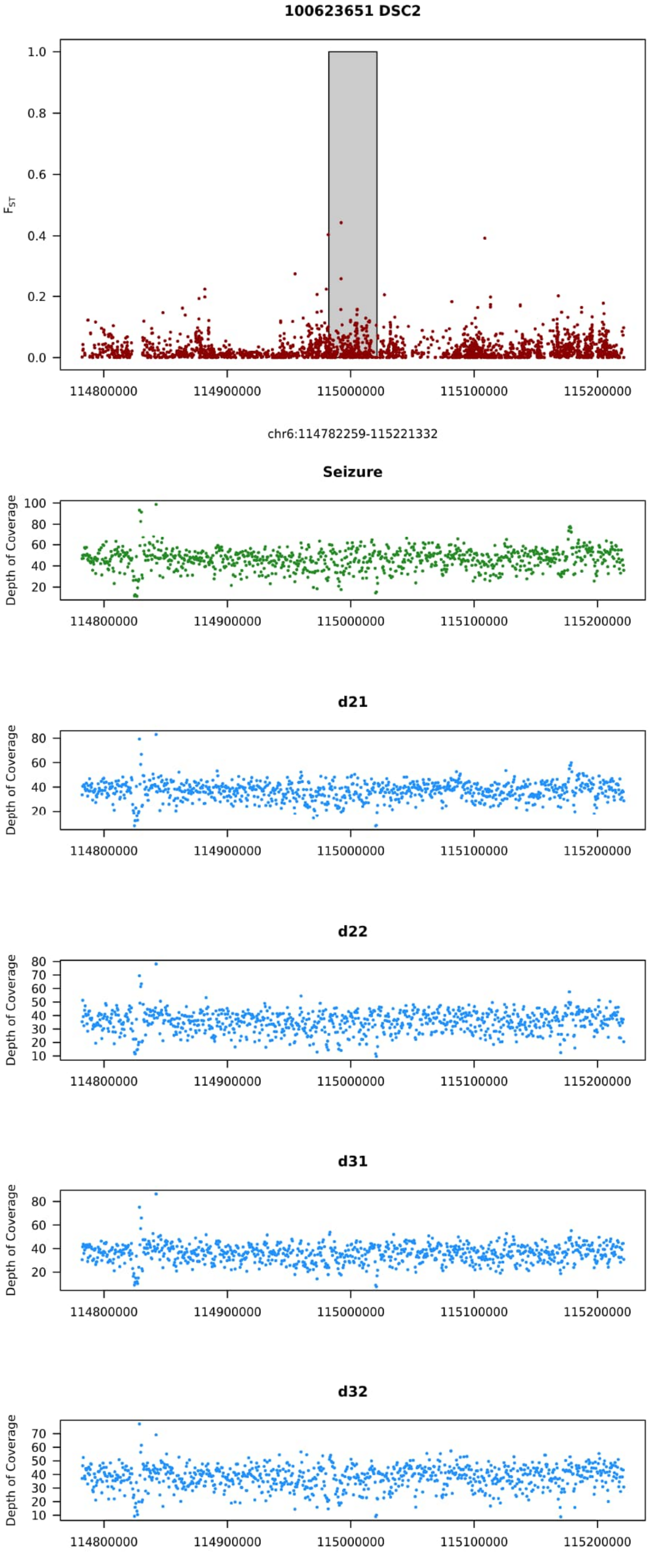
100623651 DSC2
F_ST
chr6:114782259-115221332
Seizure
d21
d22
d31
d32
Depth of Coverage
114800000
114900000
115000000
115100000
115200000

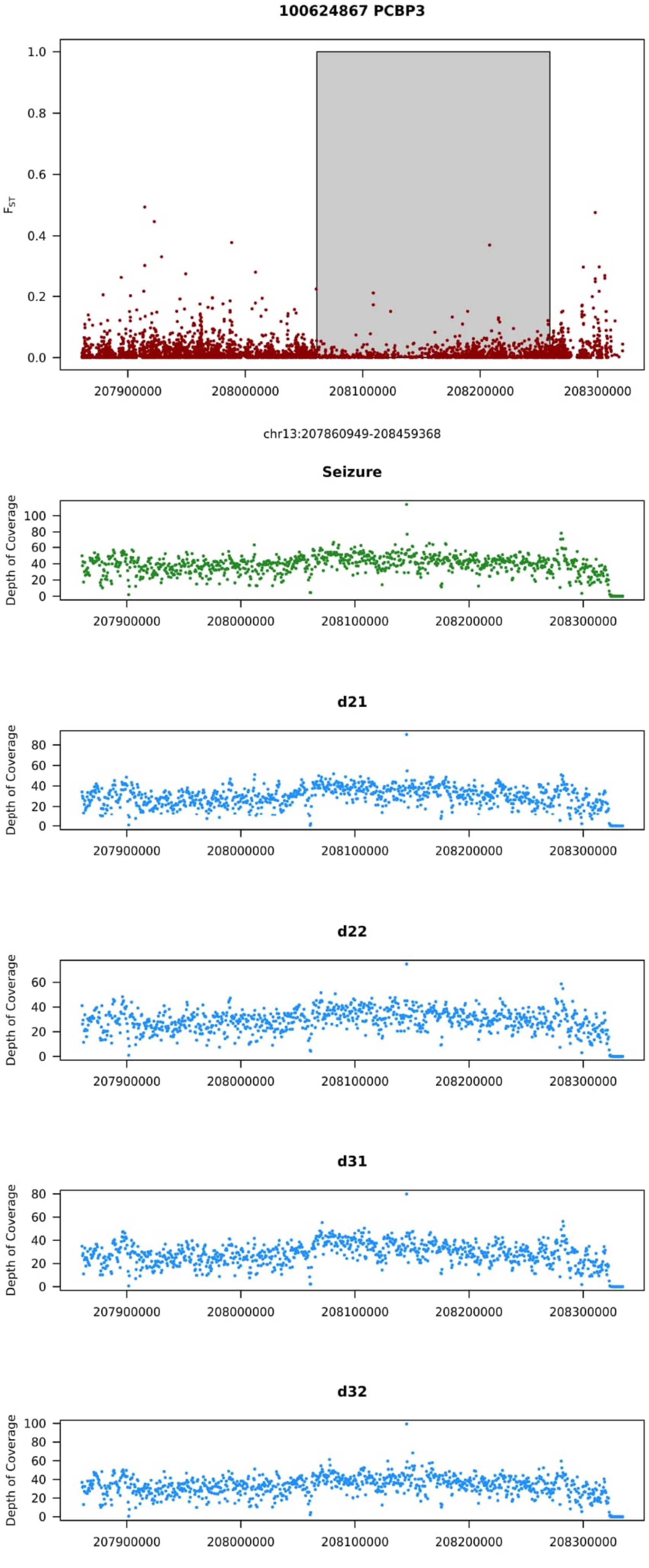
100624867 PCBP3
F_ST
chr13:207860949-208459368
Seizure
d21
d22
d31
d32
Depth of Coverage
207900000
208000000
208100000
208200000
208300000

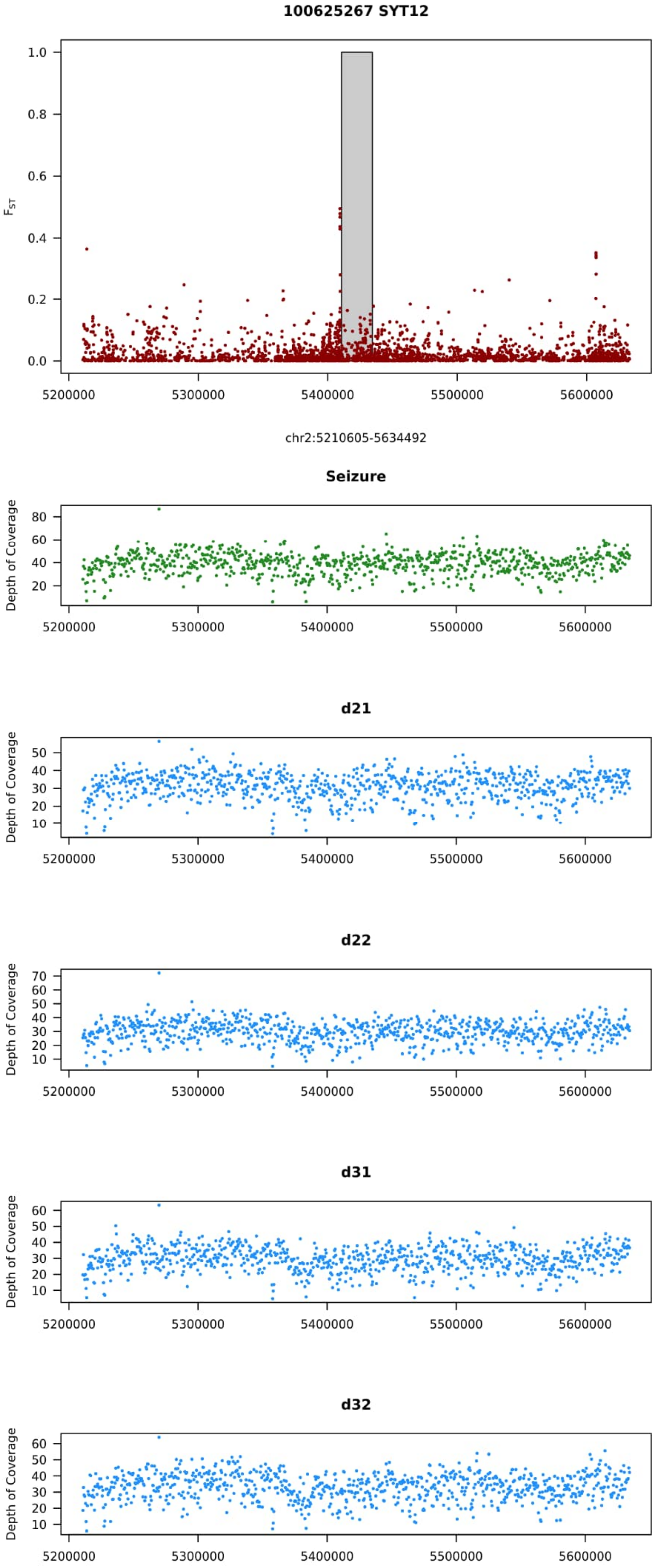
100625267 SYT12
F_{ST}
chr2:5210605-5634492
Seizure
Depth of Coverage
d21
Depth of Coverage
d22
Depth of Coverage
d31
Depth of Coverage
d32
Depth of Coverage
5200000
5300000
5400000
5500000
5600000

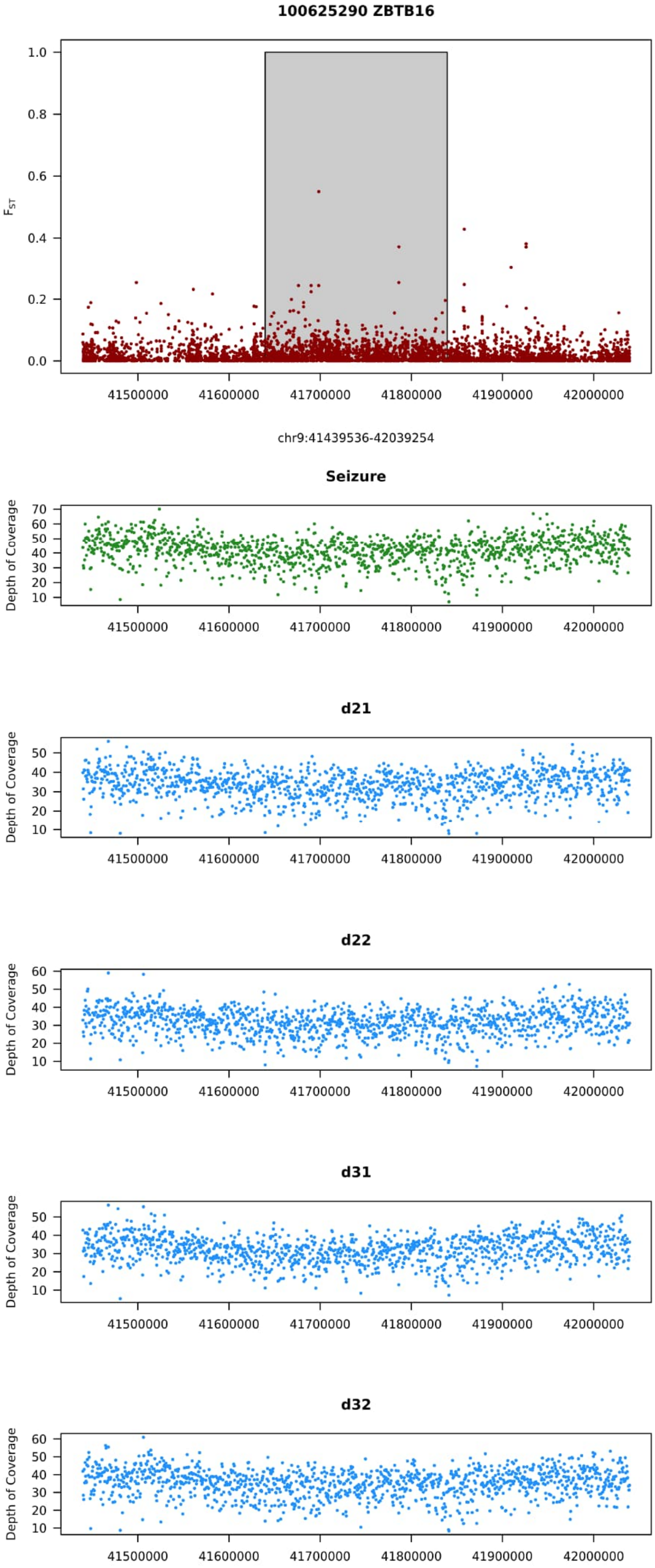
100625290 ZBTB16
F_{ST}
1.0
0.8
0.6
0.4
0.2
0.0
41500000
41600000
41700000
41800000
41900000
42000000
chr9:41439536-42039254
Seizure
Depth of Coverage
d21
Depth of Coverage
d22
Depth of Coverage
d31
Depth of Coverage
d32
Depth of Coverage

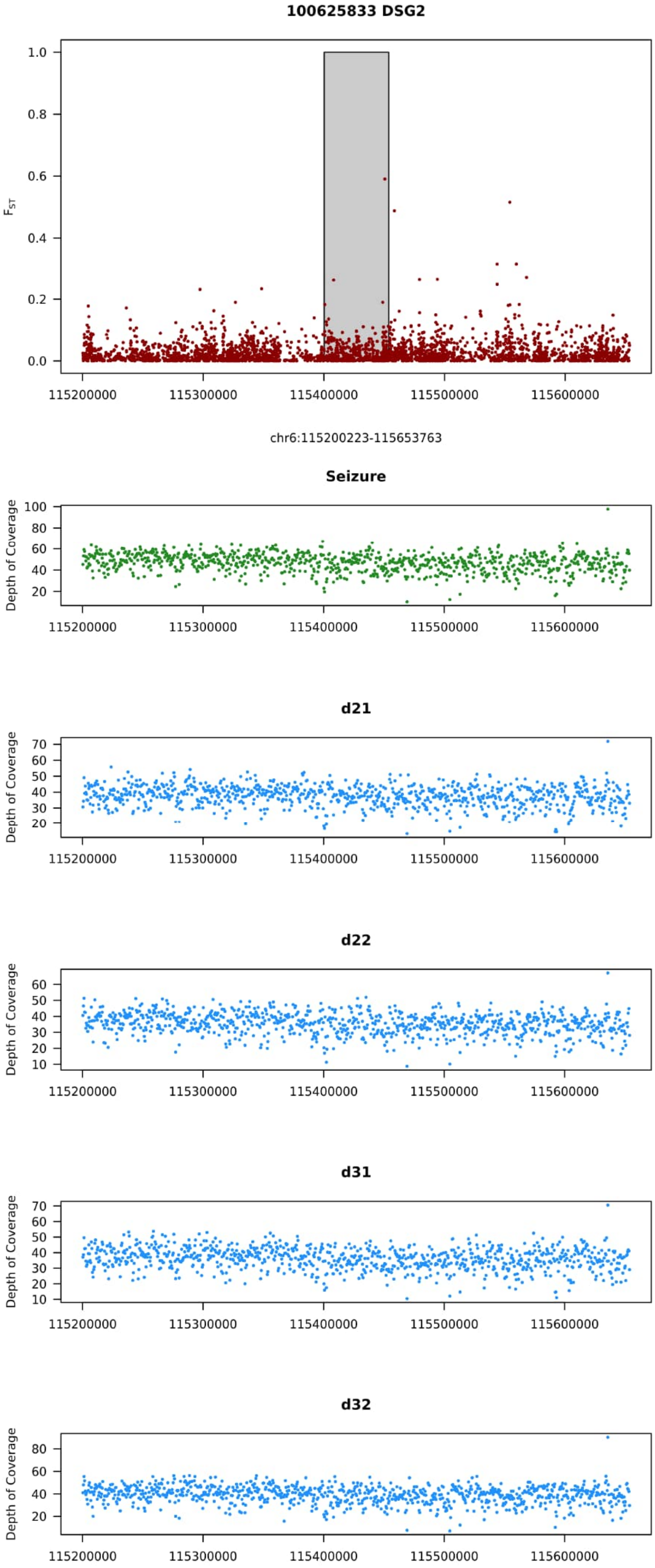
100625833 DSG2
F_ST
chr6:115200223-115653763
Seizure
d21
d22
d31
d32
Depth of Coverage
115200000
115300000
115400000
115500000
115600000

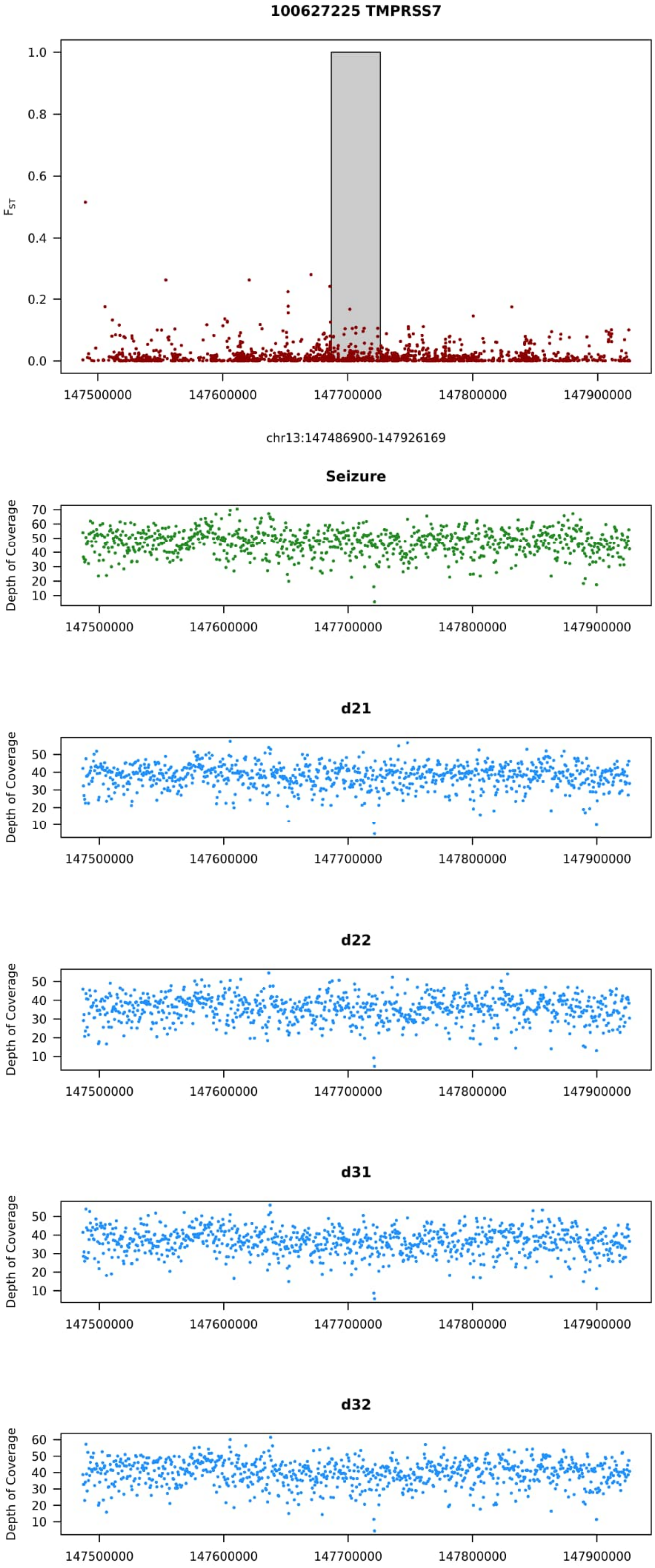
100627225 TMPRSS7
F_ST
chr13:147486900-147926169
Seizure
d21
d22
d31
d32
Depth of Coverage
147500000
147600000
147700000
147800000
147900000

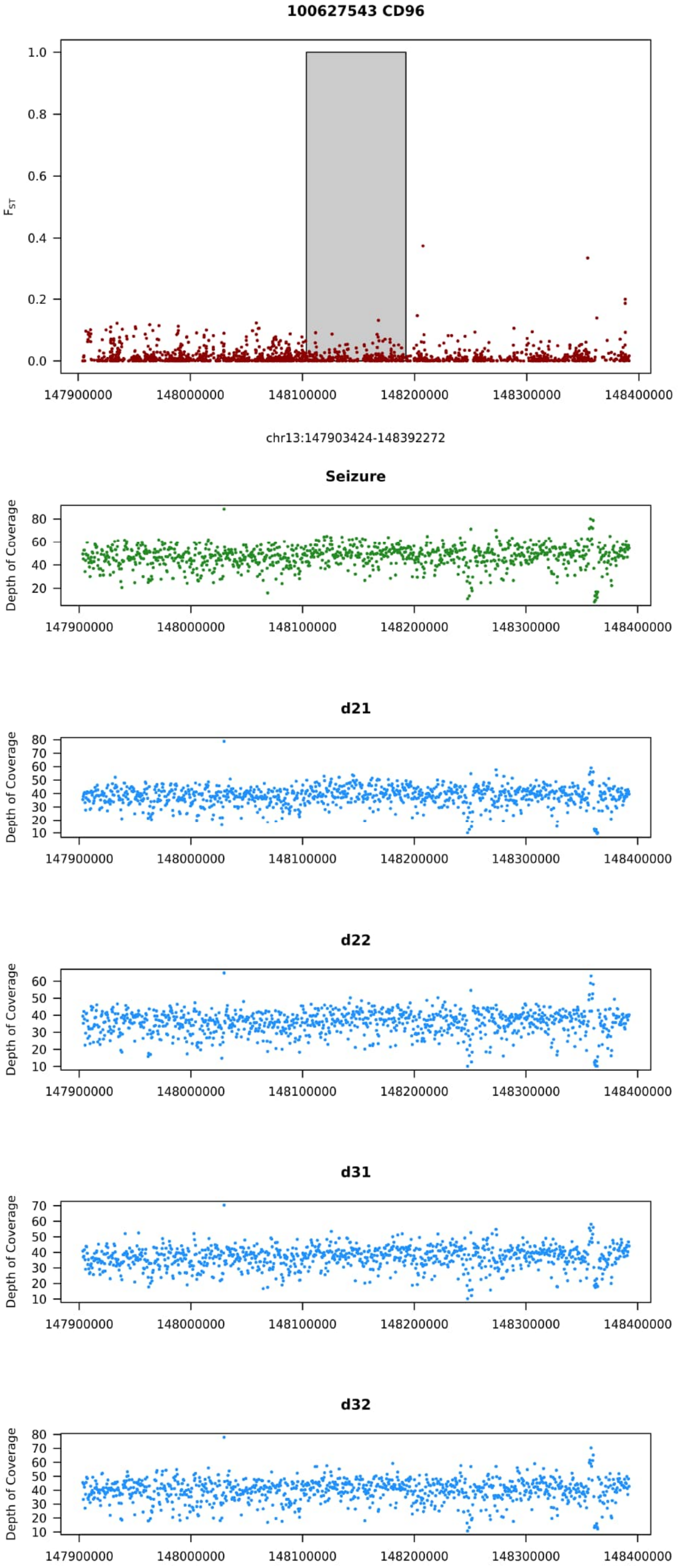
100627543 CD96
F_ST
chr13:147903424-148392272
Seizure
d21
d22
d31
d32
Depth of Coverage
147900000
148000000
148100000
148200000
148300000
148400000

100627849 HOXB6

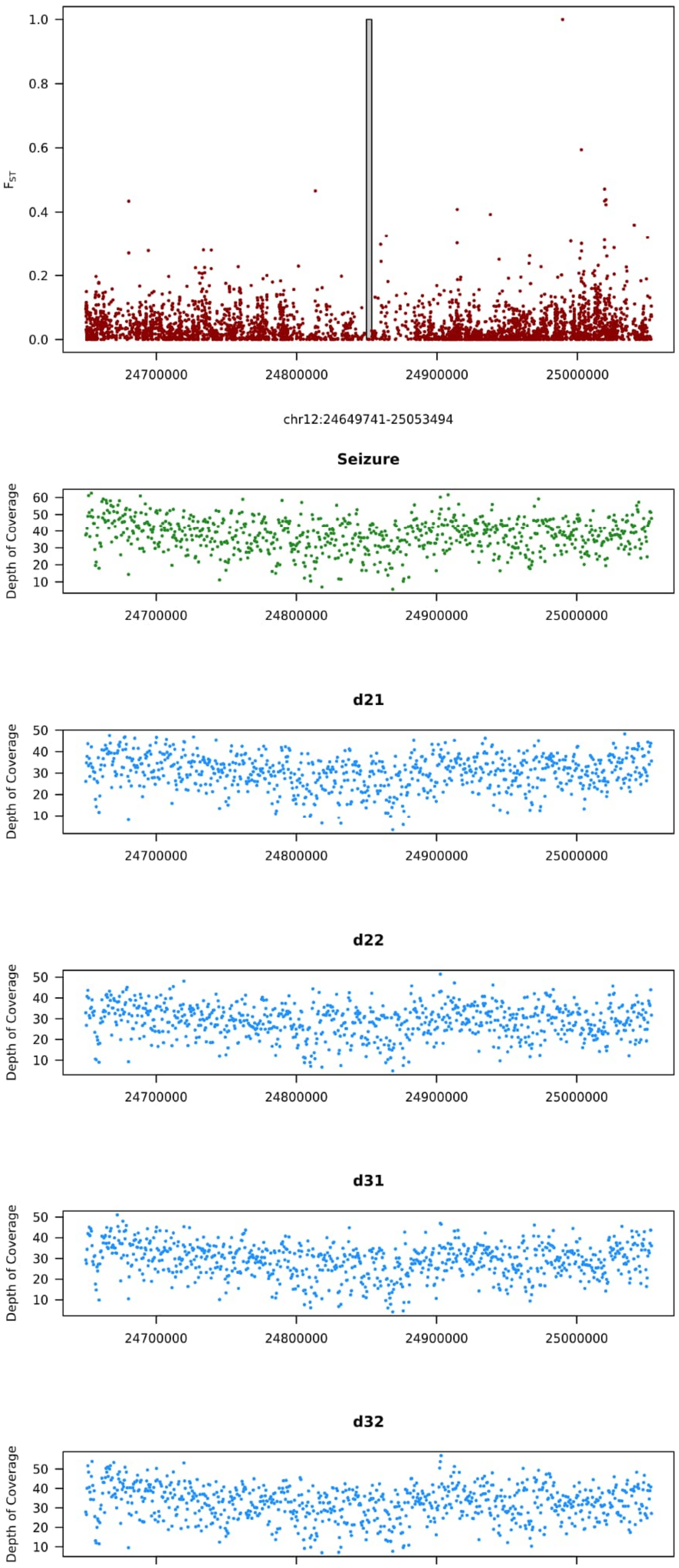

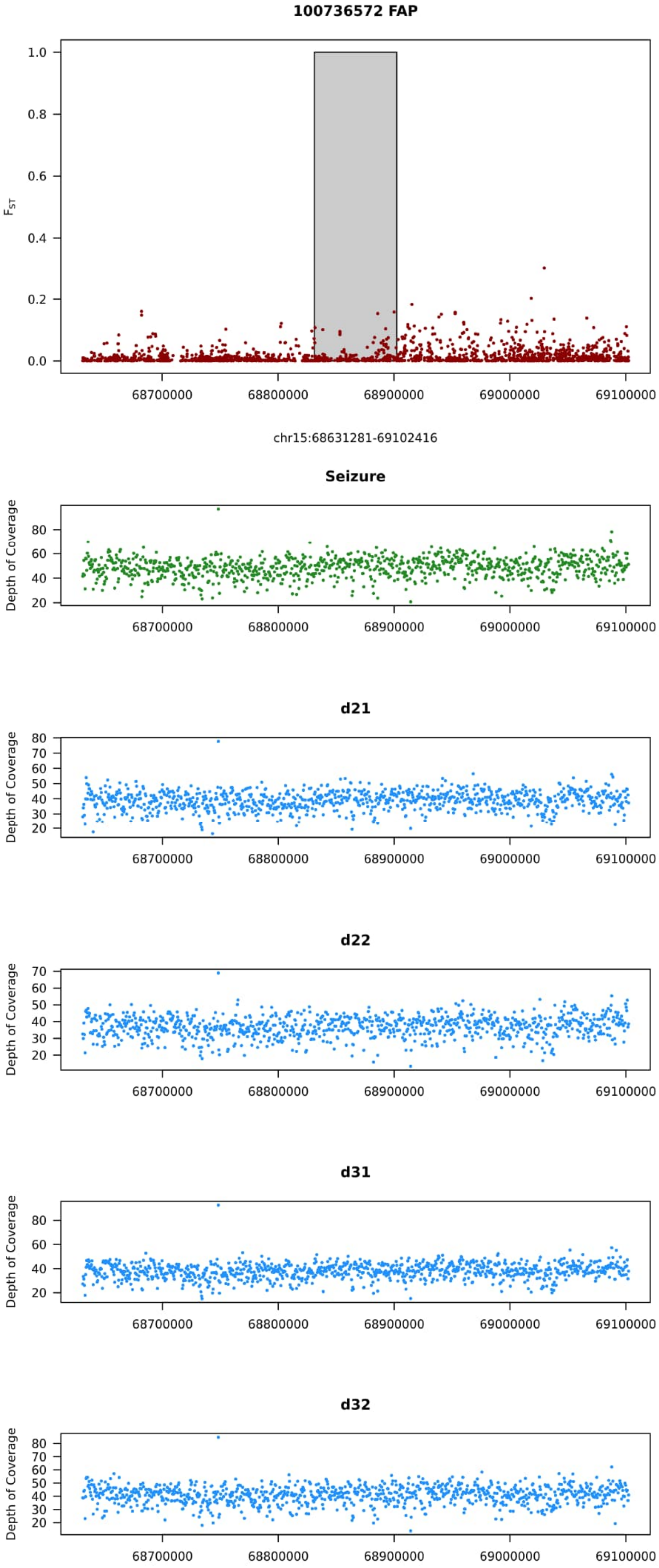

100736572 FAP
F_{ST}
0.0
0.2
0.4
0.6
0.8
1.0
68700000
68800000
68900000
69000000
69100000
chr15:68631281-69102416
Seizure
Depth of Coverage
d21
Depth of Coverage
d22
Depth of Coverage
d31
Depth of Coverage
d32
Depth of Coverage

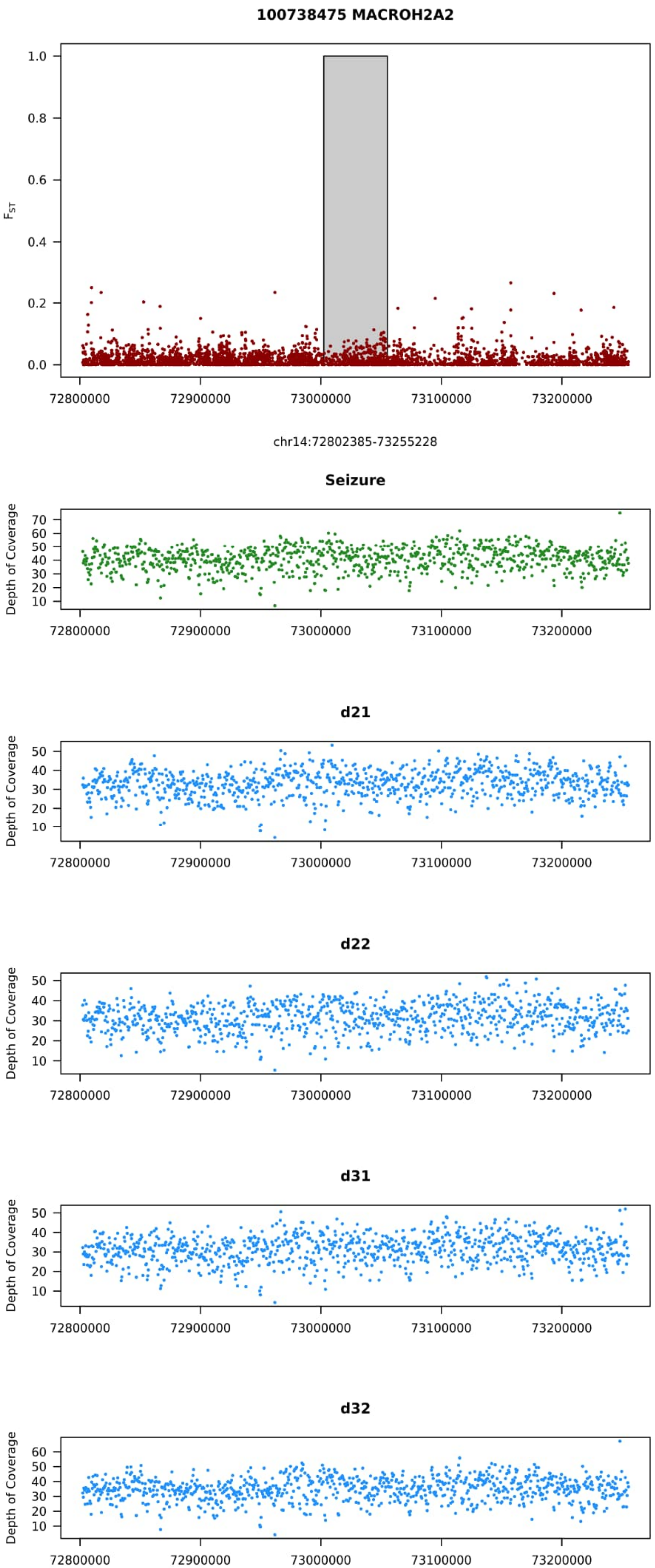

100738475 MACROH2A2
F_ST
1.0
0.8
0.6
0.4
0.2
0.0
72800000
72900000
73000000
73100000
73200000
chr14:72802385-73255228
Seizure
Depth of Coverage
d21
Depth of Coverage
d22
Depth of Coverage
d31
Depth of Coverage
d32
Depth of Coverage

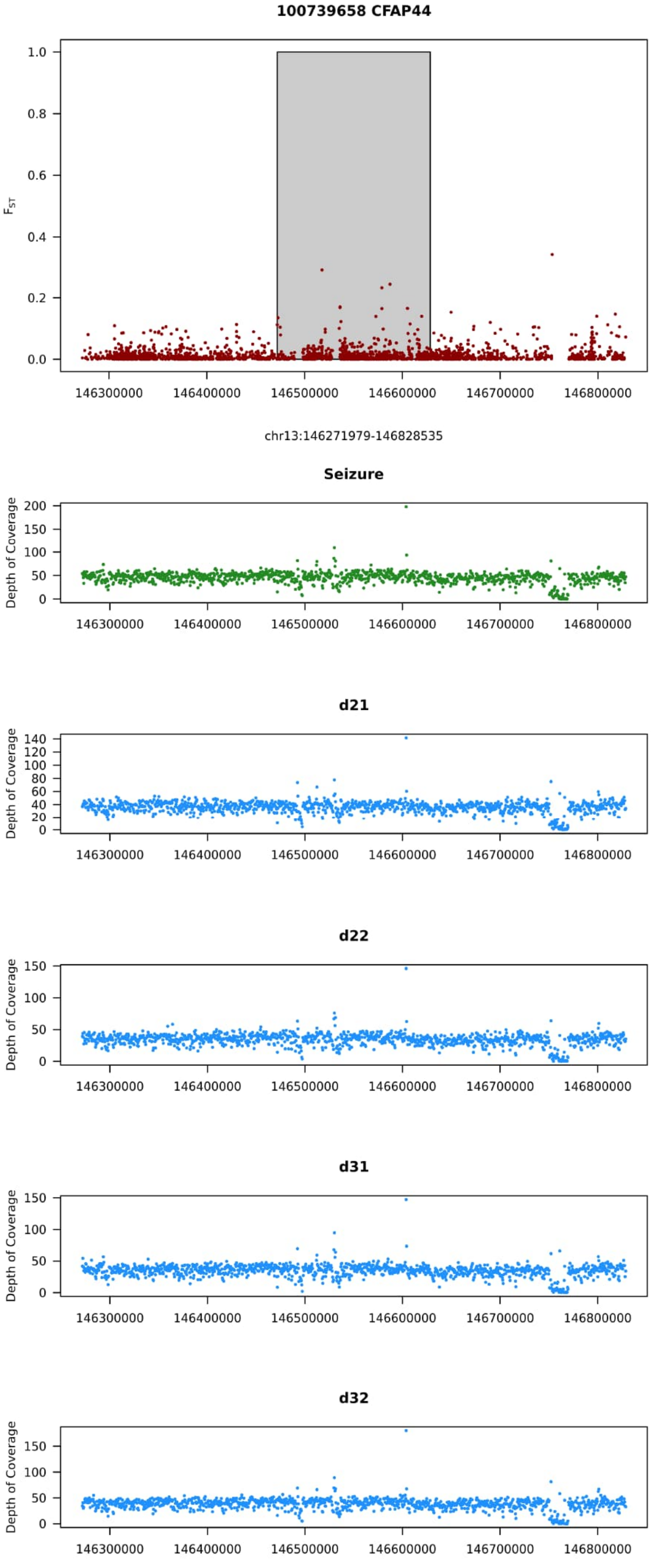

100739658 CFAP44
F_{ST}
1.0
0.8
0.6
0.4
0.2
0.0
146300000
146400000
146500000
146600000
146700000
146800000
chr13:146271979-146828535
Seizure
Depth of Coverage
d21
d22
d31
d32

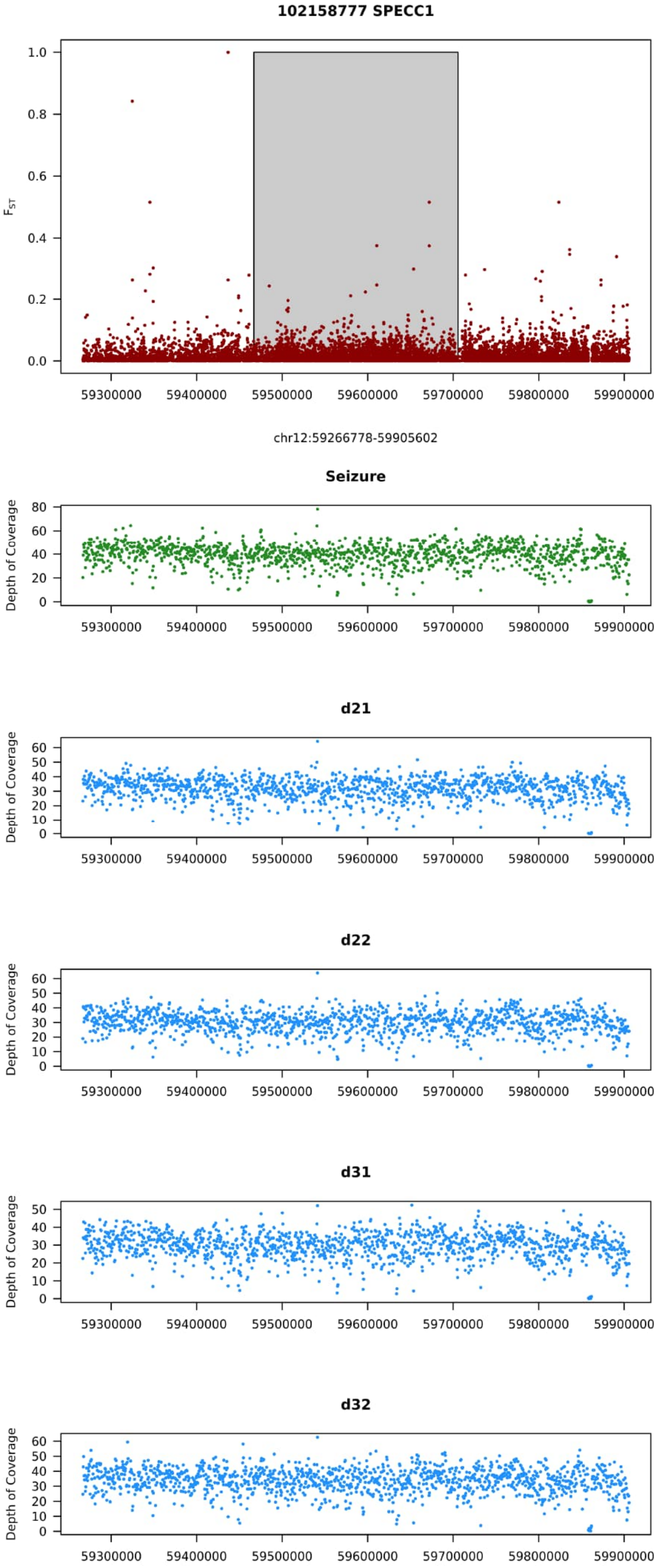

102158777 SPECC1
F_{ST}
1.0
0.8
0.6
0.4
0.2
0.0
59300000
59400000
59500000
59600000
59700000
59800000
59900000
chr12:59266778-59905602
Seizure
Depth of Coverage
80
60
40
20
0
d21
Depth of Coverage
d22
Depth of Coverage
d31
Depth of Coverage
d32
Depth of Coverage

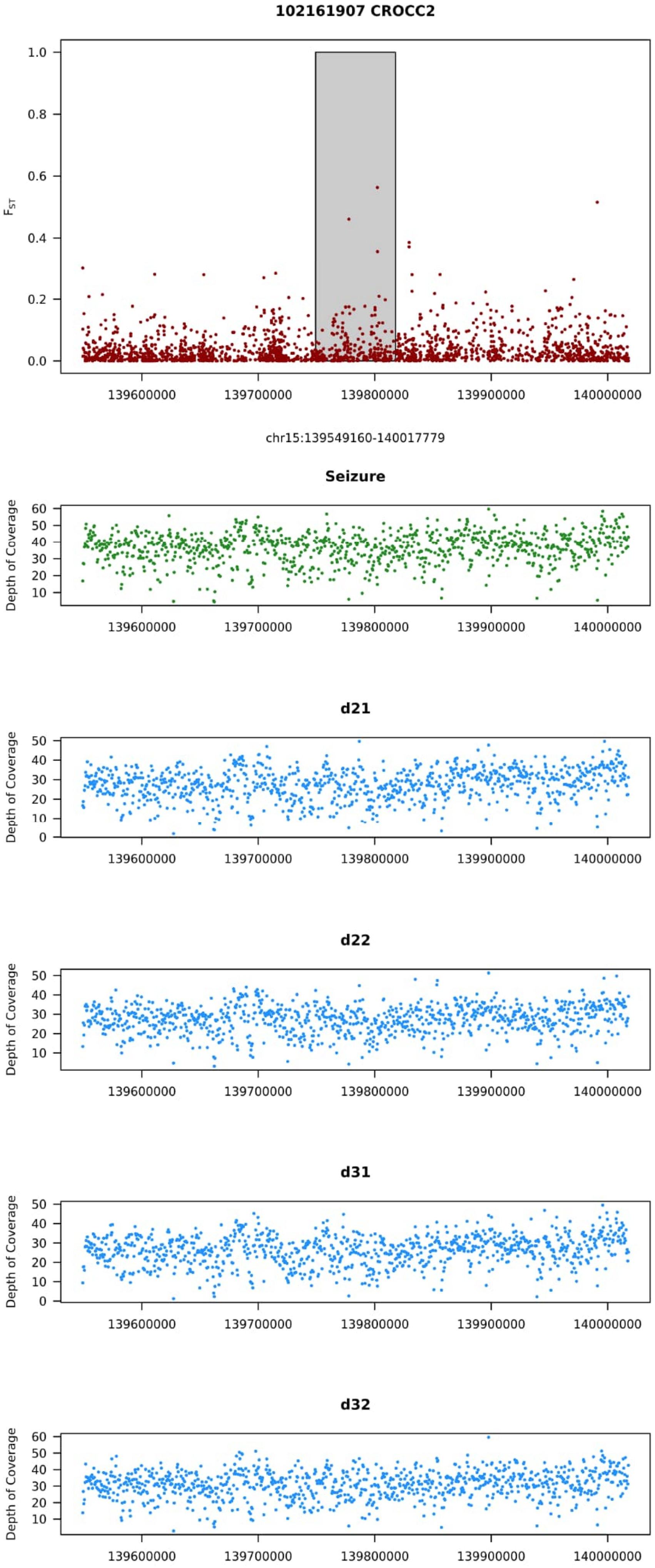
102161907 CROCC2
F_ST
chr15:139549160-140017779
Seizure
d21
d22
d31
d32
Depth of Coverage
139600000
139700000
139800000
139900000
140000000

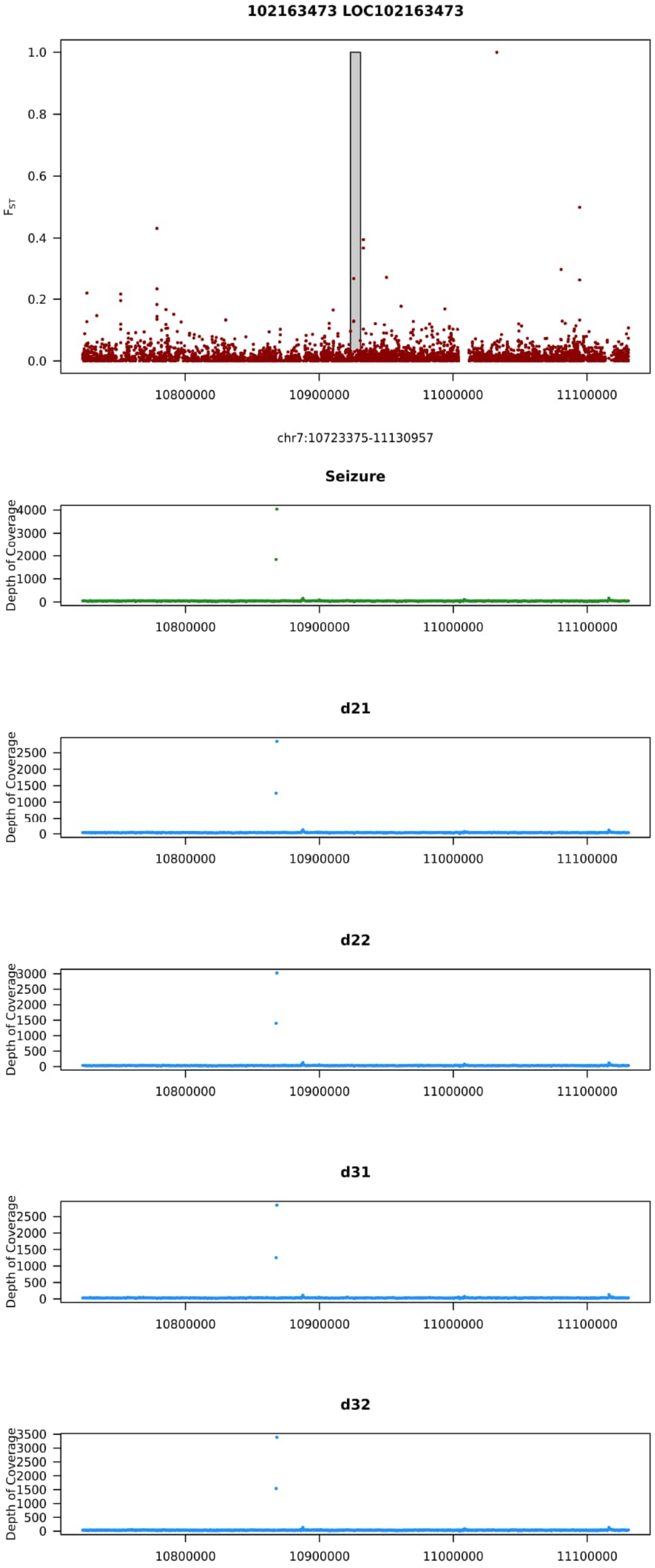

102163473 LOC102163473
F_{ST}
1.0
0.8
0.6
0.4
0.2
0.0
10800000
10900000
11000000
11100000
chr7:10723375-11130957
Seizure
Depth of Coverage
4000
3000
2000
1000
0
d21
2500
2000
1500
1000
500
d22
3000
d31
d32
3500

102165730 LOC102165730

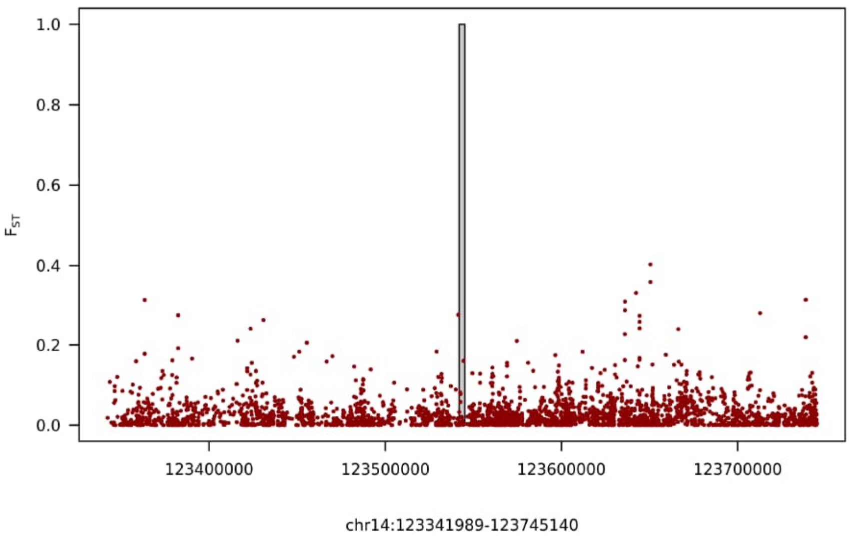

Seizure

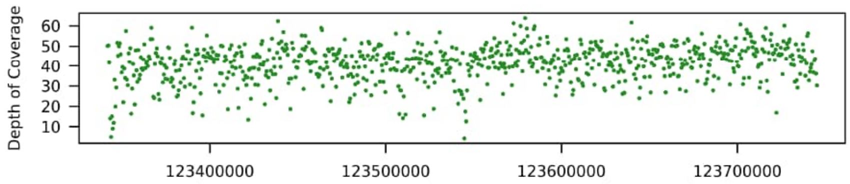

d21

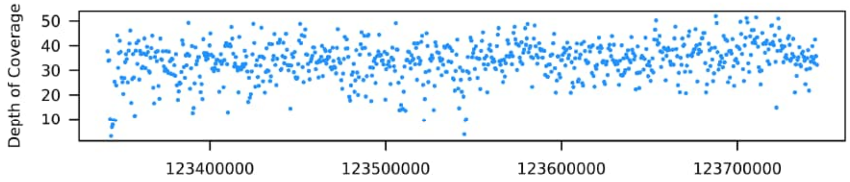

d22

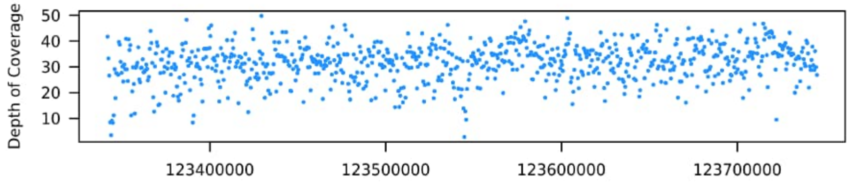

d31

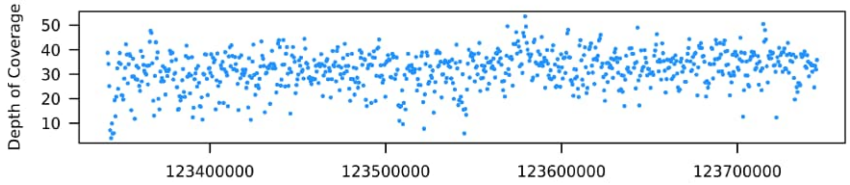

d32

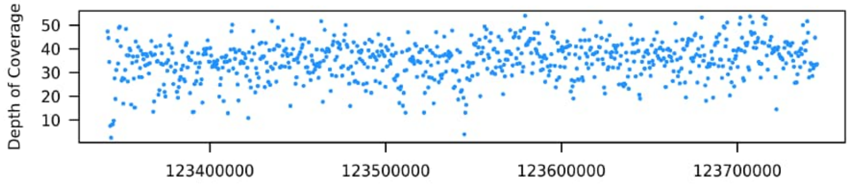

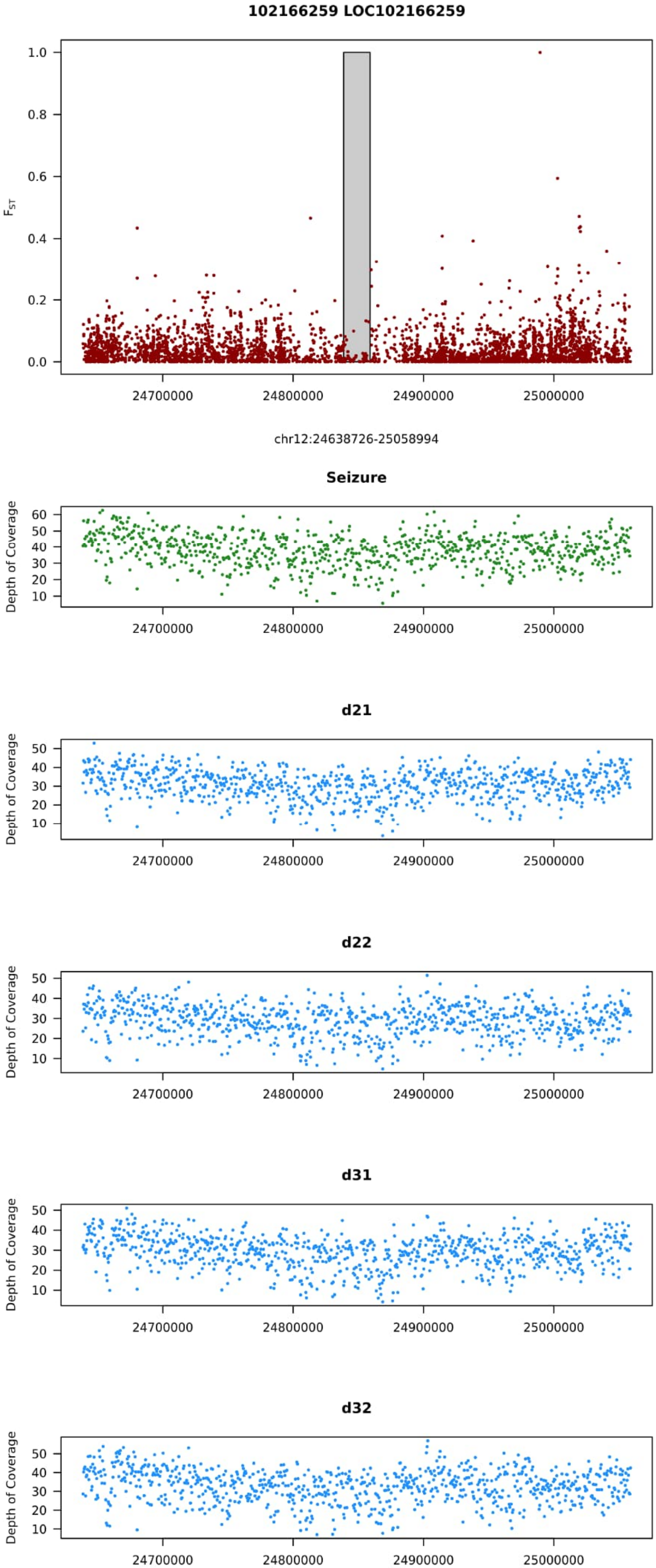

102166259 LOC102166259
F_ST
1.0
0.8
0.6
0.4
0.2
0.0
24700000
24800000
24900000
25000000
chr12:24638726-25058994
Seizure
Depth of Coverage
d21
Depth of Coverage
d22
Depth of Coverage
d31
Depth of Coverage
d32
Depth of Coverage

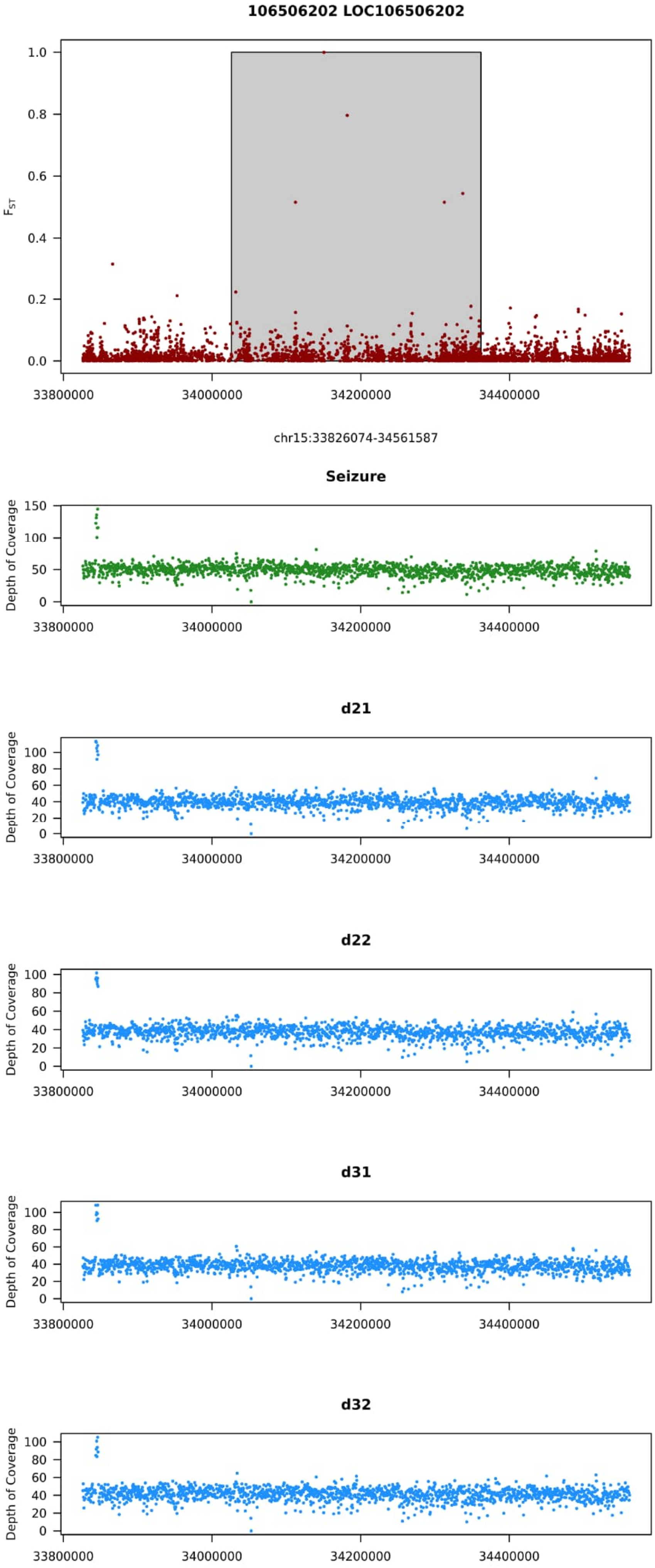
106506202 LOC106506202
F_ST
1.0
0.8
0.6
0.4
0.2
0.0
33800000
34000000
34200000
34400000
chr15:33826074-34561587
Seizure
Depth of Coverage
150
100
50
0
d21
Depth of Coverage
100
80
60
40
20
0
d22
Depth of Coverage
d31
Depth of Coverage
d32
Depth of Coverage

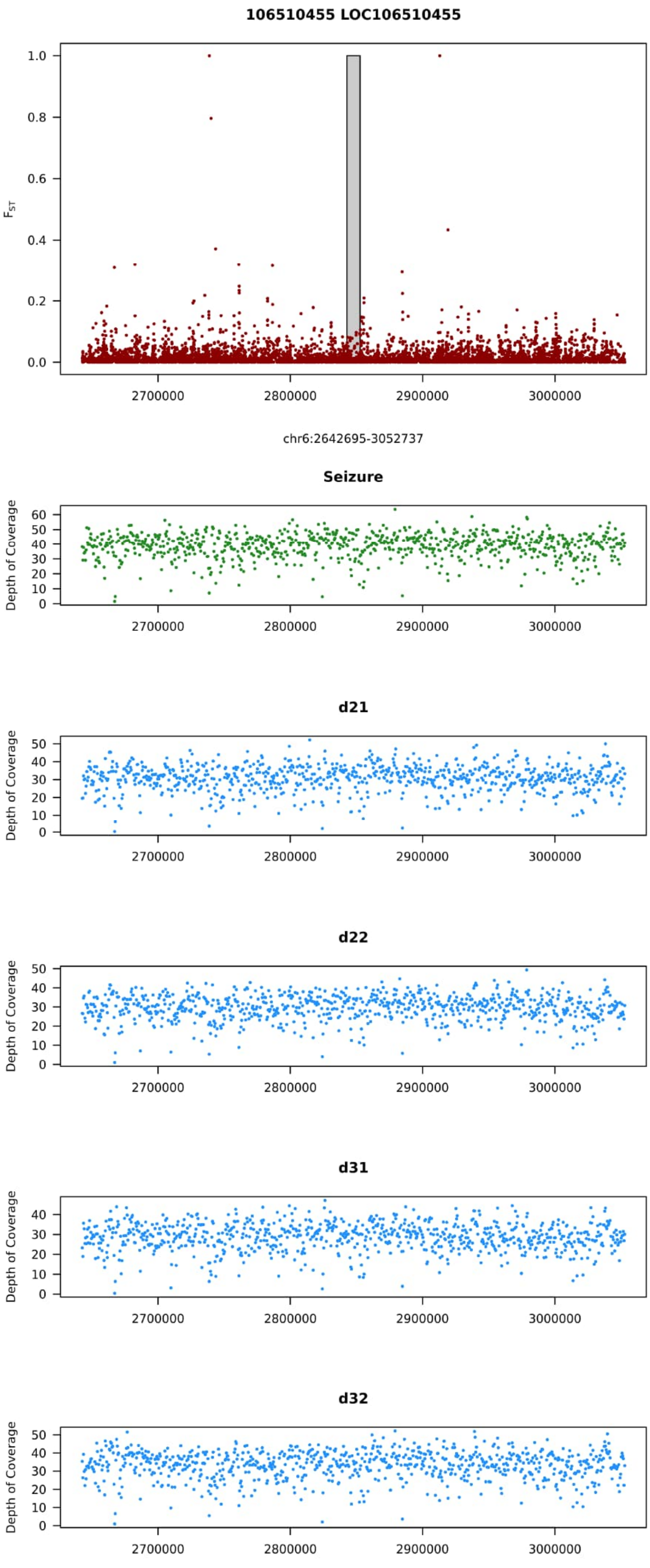

106510455 LOC106510455
F_ST
1.0
0.8
0.6
0.4
0.2
0.0
2700000
2800000
2900000
3000000
chr6:2642695-3052737
Seizure
Depth of Coverage
d21
d22
d31
d32

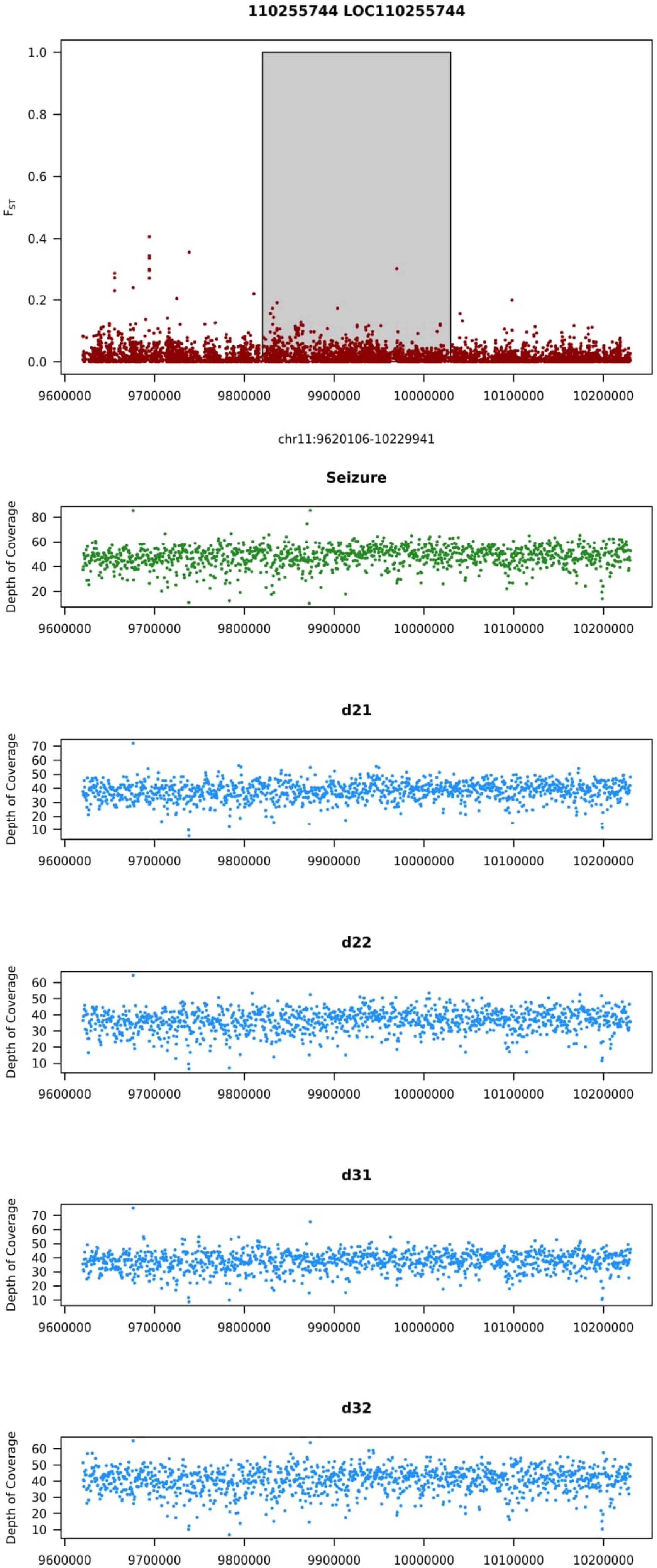
110255744 LOC110255744
F_ST
chr11:9620106-10229941
Seizure
d21
d22
d31
d32
Depth of Coverage
9600000
9700000
9800000
9900000
10000000
10100000
10200000

110256621 TBX1

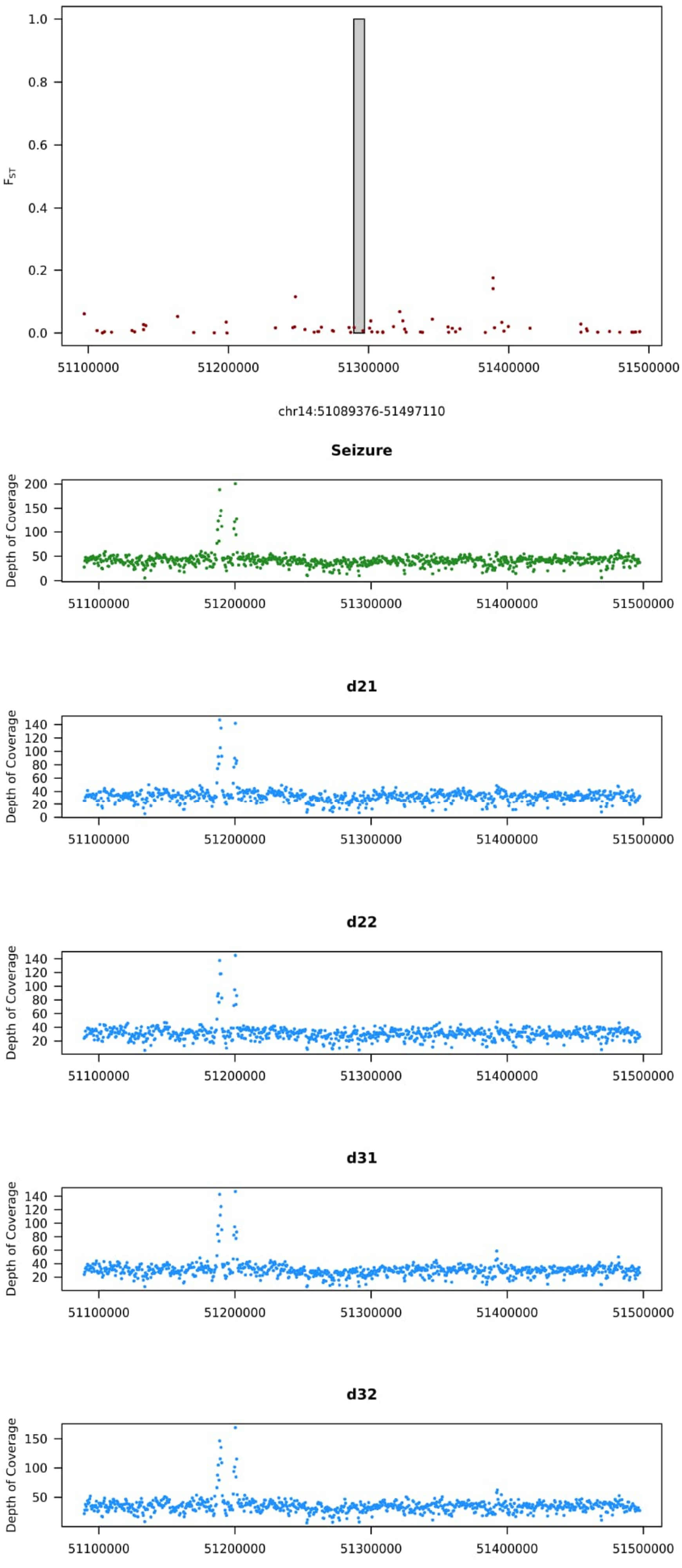

110256817 LOC110256817

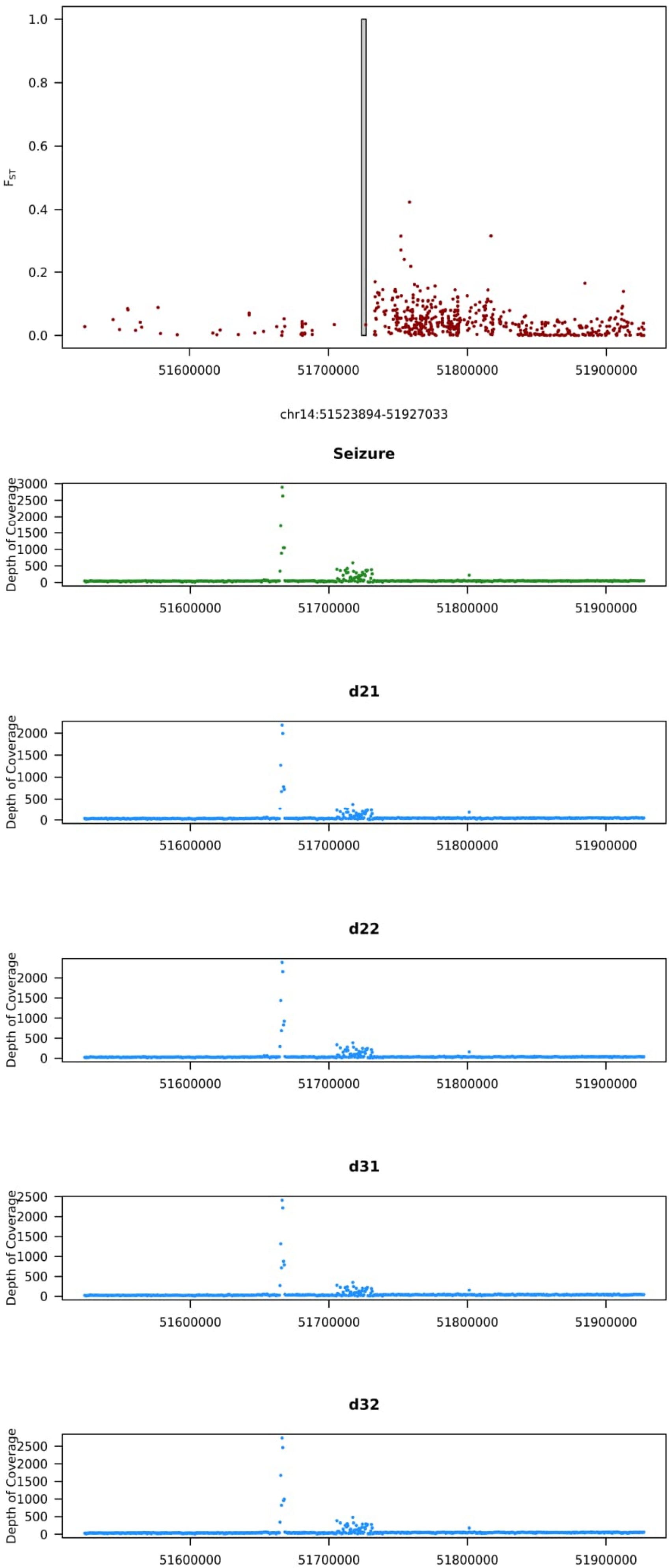

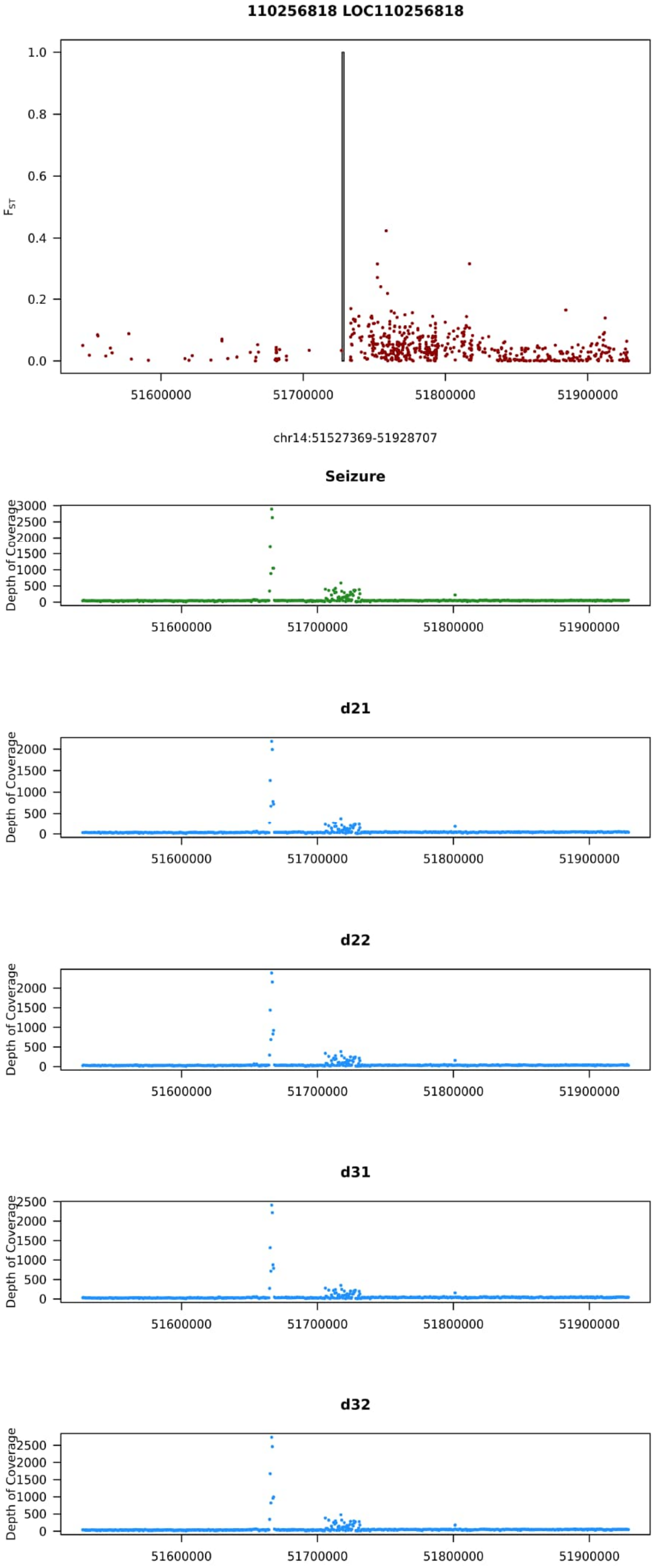
110256818 LOC110256818
F_ST
1.0
0.8
0.6
0.4
0.2
0.0
51600000
51700000
51800000
51900000
chr14:51527369-51928707
Seizure
Depth of Coverage
3000
2500
2000
1500
1000
500
0
d21
d22
d31
d32

110259223 LOC110259223

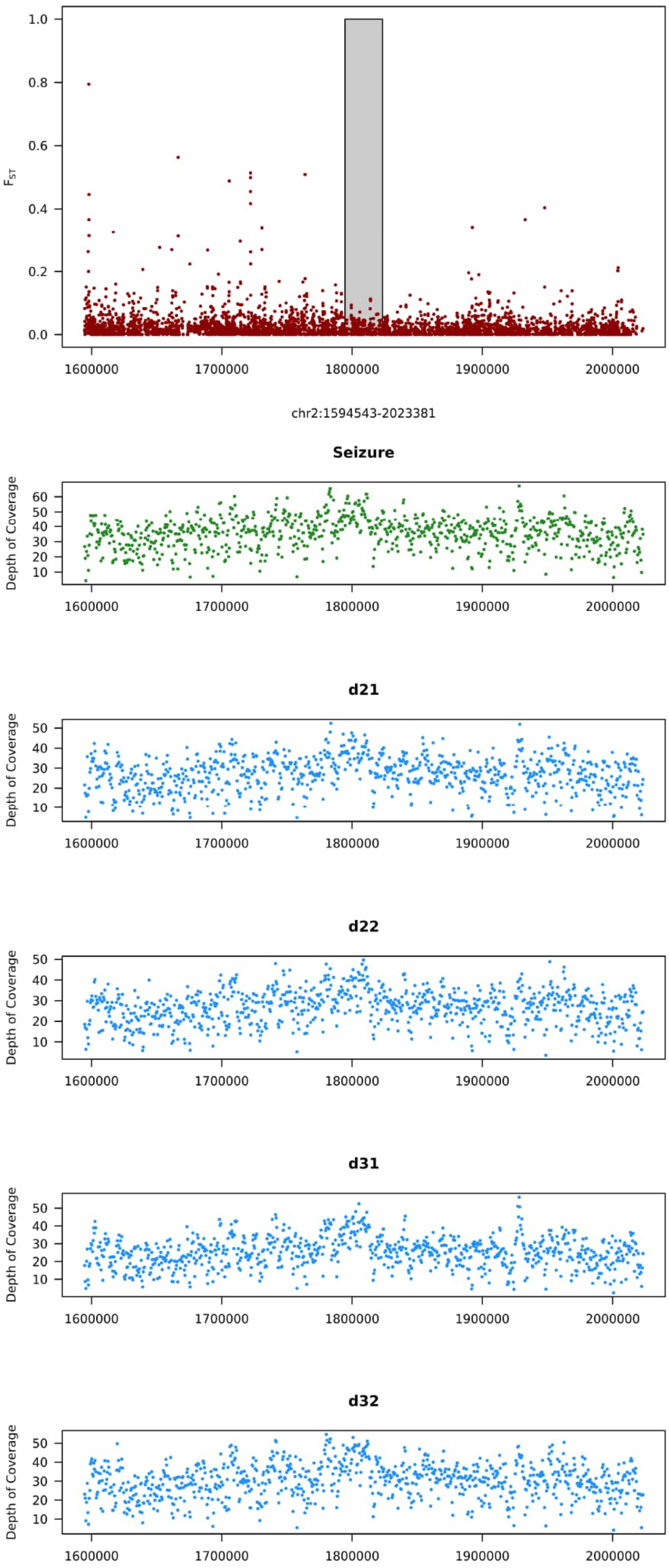

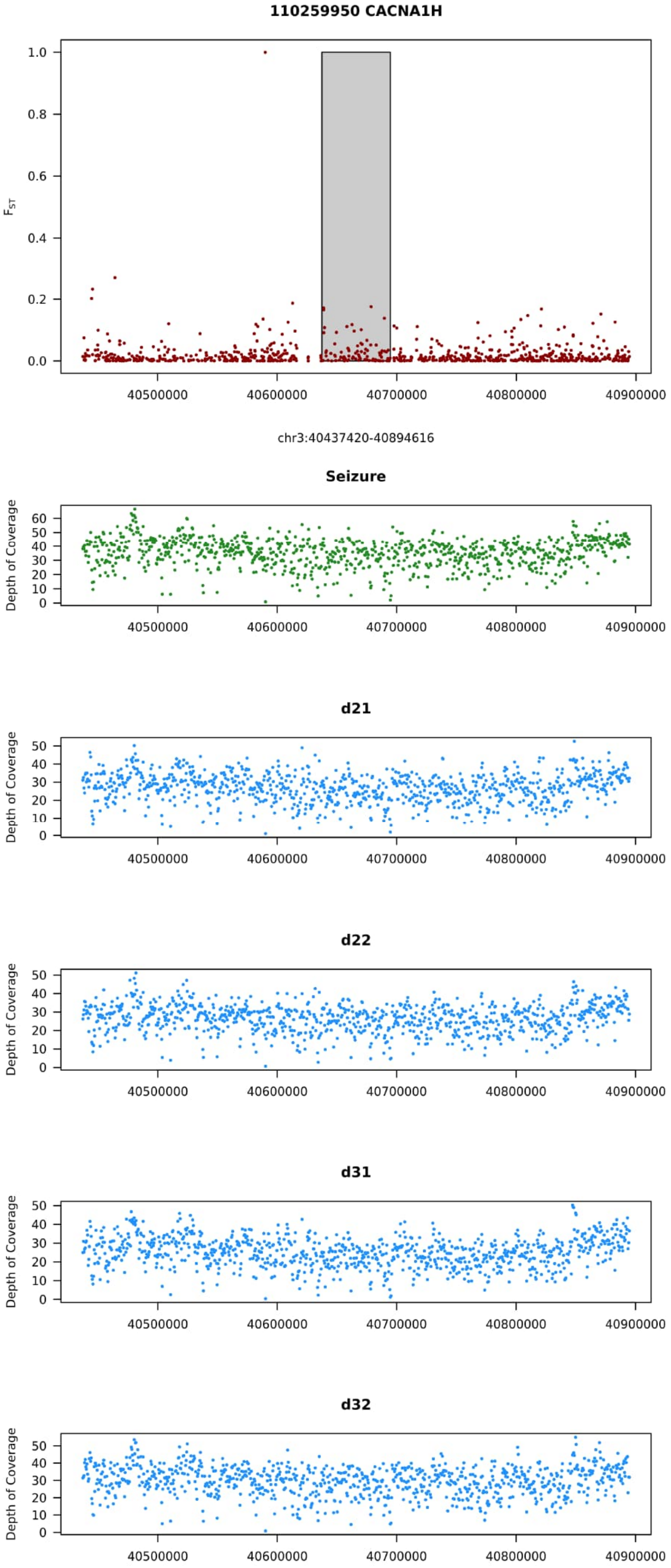

110259950 CACNA1H
FST
chr3:40437420-40894616
Seizure
d21
d22
d31
d32
Depth of Coverage
40500000
40600000
40700000
40800000
40900000

110260602 MAPK8IP2

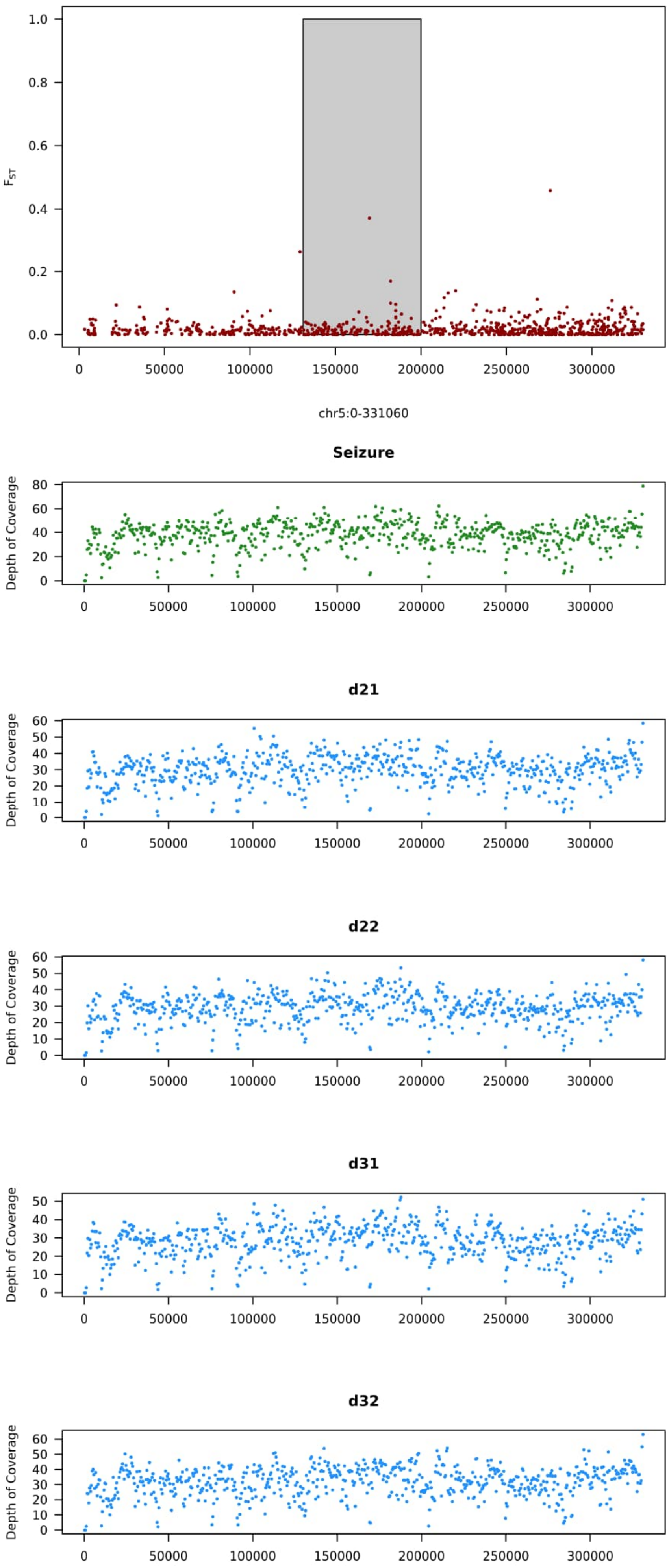

Supplement: Supplementary file 6 — Supplementary file6 (PDF 8068 KB) [file 10048_2024_750_MOESM6_ESM.pdf]
